# Binding of Steroid Hormones to the FA1 and FA6 Sites of Human Serum Albumin through Computational Biology and Quantum Biochemistry

**DOI:** 10.1021/acsomega.5c11421

**Published:** 2026-03-24

**Authors:** Victor L. B. França, André Hadad, Jackson L. Amaral, Francisco R. X. Vieira, Hernandes F. Carvalho, Valder N. Freire

**Affiliations:** † Department of Physics, Federal University of Ceará, 60440-900 Fortaleza, Ceará, Brazil; ‡ Department of Physiology and Pharmacology, Faculty of Medicine, Federal University of Ceará, 60430-160 Fortaleza, Ceará, Brazil; § Department of Biochemistry and Pharmacology, Federal University of Piaui, CEP 64049-55 Teresina, Piaui, Brazil; ∥ Department of Structural and Functional Biology, Institute of Biology, State University of Campinas, 13083-864 Campinas, São Paulo, Brazil

## Abstract

Human serum albumin (HSA) plays a significant role in the transportation of steroid hormones through noncovalent interactions of low affinity. The binding between HSA and estradiol and testosterone has been a subject of investigation through experimental tools. While some studies suggest that HSA carries sex steroid hormones through a unique binding site, others propose that this interaction occurs through two or three binding sites, indicating a lack of consensus regarding the mechanisms underlying these interactions. In view of this, the present study used molecular docking, molecular dynamics, and quantum biochemistry to obtain more insights into the binding of estradiol, dihydrotestosterone, and testosterone to HSA. Molecular docking indicated that fatty acid binding sites 1 (FA1) and 6 (FA6), located respectively in subdomain IB and between subdomains IIA and IIB, are particularly promising targets for more robust investigations. The hormones exhibited considerable flexibility within subdomain IB, with dihydrotestosterone showing the greatest structural stability. This hormone also demonstrated the highest stability within FA6, which was markedly greater than that observed at FA1. Quantum mechanics calculations suggested that the three hormones exhibit similar interaction energies for the FA1 binding site, with estradiol predicting a marginally lower energy of interaction. Dihydrotestosterone was the only hormone that exhibited both the highest structural stability and the lowest energy of interaction when bound to FA6. Overall, the results suggest that the FA1 and FA6 binding sites generally do not favor the formation of strong interactions, except in the HSA-FA6:Dihydrotestosterone complex, where the hydrogen bond LEU481­(HN-main chain):DHT­(O17) played a crucial role in stabilizing conformations of both high and low theoretical energy of interaction. This observation aligns with the established fact that the interaction between HSA and sex steroid hormones is weak. Moreover, the present study found that dihydrotestosterone exhibits a heightened tendency to bind to FA6 in comparison to estradiol and testosterone. This tendency may critically regulate DHT serum transport, bioavailability, and half-life, while also creating a pharmacologically relevant hotspot for competition with fatty acids and FA6-targeting drugs, with potential implications for hormonal homeostasis and drug-hormone interactions in physiological and pathological conditions.

## Graphical abstract

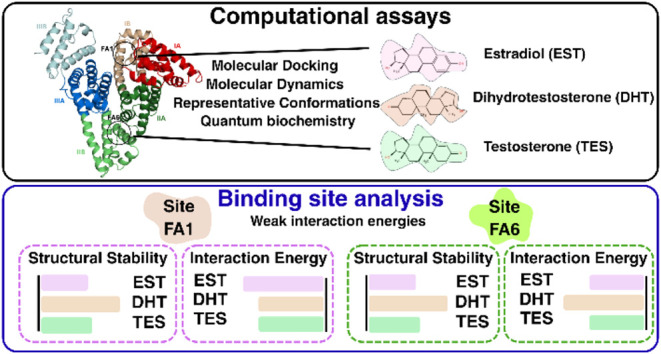

## 1 Introduction

Human serum albumin (HSA) is an essential plasma protein that exerts a notable impact on the availability and dispersion of various bioactive substances, including sex hormones like estradiol (EST), dihydrotestosterone (DHT), and testosterone (TES). The reversible attachment of HSA to a broad array of molecules plays a crucial role in regulating the movement of these hormones within the body.^1–3^ The critical role of HSA as a carrier for drugs has been extensively explored, particularly focusing on its involvement in drug transportation systems, therapeutic uses, and investigations into drug-albumin connections.^1^

HSA consists of three homologous domains (I–III), each comprising two subdomains (A and B), with a total of 585 amino acids.^4^ This is illustrated in Figure 1A. The structural flexibility of HSA enables it to adapt to a range of ligands, with nine distinct binding sites in its domains that are specifically adapted to accommodate long- and medium-chain fatty acids (FA).^5^ The IIA subdomain of HSA is a critical binding site for drugs and displays hydrophobic characteristics that regulate its behavior in the presence of specific ligands.^6^ The intricate structural organization and binding capacity of HSA make it a crucial player in processes such as absorption, distribution, metabolism, and excretion of small molecules, highlighting its indispensable role in physiological functions.

**Figure 1:**
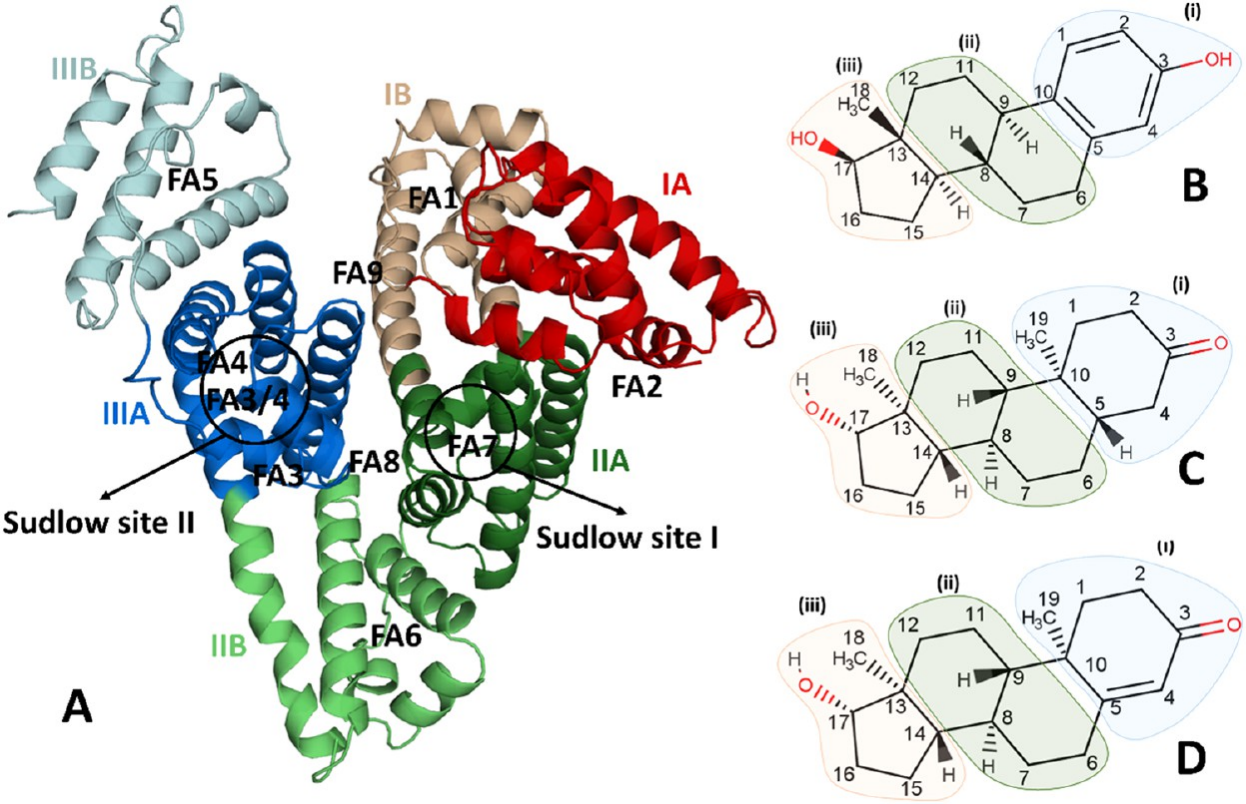
Structures of human serum albumin (HSA) and sex hormones. (A) Overall structure of HSA with its subdomains (IA–IIIB) identified by different colors. Chemical structure for (B) estradiol (EST), (C) dihydrotestosterone (DHT), and (D) testosterone (TES).

EST, DHT, and TES play crucial roles in various physiological processes, including the development and maintenance of sexual characteristics, the regulation of bone and muscle, and the maintenance of bodily balance. These hormones interact with HSA in complex ways, significantly impacting hormone biology and clinical practices. EST and TES influence brain tissue, neuronal myelination, cognition, and neuroprotection.^7^ Furthermore, these hormones have been demonstrated to exert organizational and activational effects on the brain, thereby potentially influencing the development of depressive disorders.^8^

Steroid hormones are transported by HSA. Some data suggest that these hormones may be anchored to fatty acid binding sites 1 (FA1), 2 (FA2), 3 (FA3), and 6 (FA6).^9–11^ FA1, located in the center of the IB subdomain, is principally composed of amino acid residues ARG114, TYR138, HIS146, TYR161, and LYS190. FA2 is a modulatory site located between subdomains IA, IB, and IIA, which have TYR150, ARG257, and SER287 as critical hydrogen bond mediators responsible for stabilizing the head-groups of FA. FA3, usually coupled to FA4, is located within the IIIA subdomain and is responsible for anchoring many aromatic carboxylates. FA6 is a cleft positioned between subdomains IIA and IIB and is recognized to be significantly different from FA1-FA5 because it does not have specific amino acid residues that stabilize the FA chains.

HSA plays a significant role in hormone transport due to its high plasma concentration. Reports suggest that 33–54% of TES circulating in plasma is carried by HSA.^11^ However, it exhibits a lower affinity for steroid sex hormones, including EST, DHT and, TES, compared to Sex Hormone Binding Globulin (SHBG).^4,12^ While SHBG and other carrier proteins exhibit greater specificity and affinity for these hormones, the high concentration of HSA in the blood helps to mitigate fluctuations in hormone levels and maintain their fractions in a free state. A prior study, supported by affinity data, has indicated that HSA functions as a transient reservoir, thereby modulating local concentrations of hormones with considerable swiftness.^9^ This makes HSA a pivotal participant in the overall regulation of circulating steroid hormones.^12^ The essential participation of HSA in steroid hormone functions in distinct tissues can be affected by specific conditions, such as diabetes mellitus,^13^ liver diseases,^14,15^ administration of drugs,^16^ and changes in physiological concentrations of citrate.^9^ In view of this, comprehending the relationships between HSA and sex hormones is essential for understanding their physiological impacts and medical consequences, highlighting the significance of HSA in the conveyance and breakdown of these vital bioactive compounds.

The binding of EST to HSA has been extensively investigated using a range of techniques, including fluorescence spectroscopy, isothermal titration calorimetry, and molecular modeling. Significant conformational and fluorescence quenching changes were identified, with van der Waals forces and hydrogen bonds playing a predominant role in the binding process. Molecular modeling indicated that EST is located in the IB subdomain of HSA and contributes to a comprehensive understanding of the binding mechanisms of EST and other ligands to plasma proteins.^10^ Despite studies investigating the binding of steroid hormones to HSA and other serum albumins, there is a lack of comparative analysis to elucidate the structural differences and similarities of the binding modes between HSA and essential hormone steroids, such as EST, DHT, and TES. The chemical structures of EST, DHT, and TES are shown in Figure 1B–D, with the compounds divided into three regions (i, ii, and iii) for the purpose of analyzing interactions.

Molecular modeling techniques, such as molecular dynamics (MD) and molecular docking, are highly significant in elucidating protein–ligand interactions, providing detailed insights into binding mechanisms at the atomic level.^17,18^ These simulations have been extensively utilized to examine protein systems, such as food proteins, drug targets, and protein–protein interactions. These simulations have yielded valuable insights into binding conformations, hotspots, affinity, and key residues, which are often challenging to ascertain through conventional experimental methods.^17,19,20,20^ The integration of MD simulations with sophisticated computational techniques and machine learning algorithms empowers researchers to enhance the precision and efficiency of protein–ligand interaction studies. This, in turn, may accelerate the process of drug discovery and understanding of biological functions at the molecular level.^18,21,22,22^

In the present study, a research approach was implemented to outline the noncovalent interactions between HSA and EST, TES, and DHT. This research was based on a well-established approach that combines molecular docking and dynamics simulations, conformational ensembles, molecular fractionation with conjugate caps (MFCC), and quantum biochemistry analysis.

## 2 Material and Methods

### 2.1 Obtaining and Preparing the Structures of HSA, Estradiol (EST), Dihydrotestosterone (DHT) and Testosterone (TES)

The three-dimensional conformation of Human Serum Albumin (HSA) was retrieved from the Protein Data Bank (PDB) under the code 4L9K, exhibiting a resolution of 2.40 Å.^23^ This HSA was chosen because it is complexed with camptothecin, a polycyclic molecule with a similar width to that of steroid hormones. The molecular structures of EST, DHT, and TES were retrieved from PubChem, with corresponding Compound Identifiers (CIDs) of 5757, 10635, and 6013, respectively. Discovery Studio 2016 was employed to eliminate water molecules and minor ligands from the crystal structure of HSA. The ProteinPrepare web server was then used to ascertain the protonation states of the HSA residues and to incorporate hydrogen atoms at a physiological pH of 7.4.^24^ A charge analysis at pH 7.4 for the three-dimensional structures of EST, DHT, and TES was conducted using MarvinSketch 18.24 (Marvin Beans Suite, ChemAxon), followed by geometric optimization within Discovery Studio 2016.

### 2.2 Molecular Docking

The molecular structures of EST/DHT/TES and HSA were initially prepared employing AutoDockTools version 1.5.6, developed by The Scripps Research Institute. Subsequently, the Autodock Vina version 1.1.2 was employed to execute the blind docking calculations, also produced by The Scripps Research Institute,^25^ which incorporates the quasi-Newton and Broyden-Fletcher-Goldfarb-Shanno (BFGS) algorithms.^26^ AutoDockTools facilitated the retention of the polar hydrogen atoms in HSA, the selection of the EST/DHT/TES torsion tree, and the incorporation of partial charges for EST/DHT/TES and HSA through the application of unified Kollman charges.^27^ While maintaining the rigidity of the receptor (HSA), the ligand (EST/DHT/TES) was permitted to exhibit flexibility. The dimensions of the grid box were established at 92 Å × 76 Å × 62 Å for HSA:EST, HSA:DHT, and HSA:TES, with HSA positioned at the center. The exhaustiveness parameter was set to 16, while all other parameters were left at their default values. Subsequently, the analysis and classification of the top 20 poses were conducted, with the classification based on the binding site, docking score, and docking frequency. The binding mode of the lowest energy of interaction in each of the two binding sites with the highest docking frequencies in each complex was simulated by using molecular dynamics.

### 2.3 Molecular Dynamics (MD)

GROMACS version 2023.1 was utilized to integrate the ligand with HSA within an aqueous environment, thereby conducting a series of simulations, including energy minimization, equilibration phase (NVT and NPT), and MD simulations. The CHARMM36 force field was selected to direct the interatomic potentials.^28^ The parameters corresponding to the atomic coordinates of the binding sites for EST, DHT, and TES were derived from CgenFF^29^ and applied in GROMACS.

Six distinct complexes were constructed: HSA:EST-FA1, HSA:EST-FA6, HSA:DHT-FA1, HSA:DHT-FA6, HSA:TES-FA1, and HSA:TES-FA6. Each complex was positioned within an individual water box (utilizing the TIP3P model) with an ionic concentration of 0.15 M (comprising Na^+^ and Cl^–^ ions). The generated complexes underwent energy minimization utilizing the steepest descent algorithm until the maximal force within the system fell below 1,000 kJ·mol^–1^·nm^–1^. Subsequently, 500,000 steps of NVT simulations were performed employing a velocity-rescaling thermostat (V-rescale thermostat) to achieve thermal equilibrium at approximately 300 K. Subsequent to this, an additional 500,000 steps of NPT simulations were conducted employing Berendsen’s isotropic pressure coupling as a sequel to the NVT phase, wherein the Berendsen barostat was utilized in conjunction with the V-rescale thermostat to regulate both the temperature and pressure of the system throughout the NPT equilibration phase.^30^ The temporal integration of the NVT and NPT simulations was executed through a leapfrog algorithm with a time increment of 2 fs, facilitated by the application of the LINCS algorithm and the distribution of hydrogen mass. The preference for the leapfrog algorithm over alternative methodologies is driven by its exceptional stability in energy preservation, ease of implementation, and efficacy in modeling intricate systems. The Verlet cutoff method was employed to assess nonbonded interactions, with a distance threshold of 1.0 nm for both van der Waals and Coulomb interactions in real space. Periodic boundary conditions coupled with the particle mesh Ewald method were employed to address the long-range electrostatic components of the Coulomb interactions. Ultimately, MD simulations spanning 100 ns were conducted for each complex.

The root-mean-square deviation (RMSD) pertaining to the atomic positions of HSA, EST, DHT, and TES was computed for all molecular dynamics conformations present in their respective computational samples by employing the *gmx rms* module within the GROMACS software framework. In addition, data of root-mean-square fluctuation (RMSF) of HSA residues were extracted by using the *gmx rmsf* GROMACS package. The atomic coordinates obtained through this methodology were subsequently employed for all calculations concerning nonbonded interactions.

### 2.4 Nonbonded Interactions Description

The investigation of noncovalent interaction employed the Discovery Studio Receptor–Ligand Interactions package to generate two-dimensional representations of the interactions occurring between HSA residues and EST/DHT/TES from a static viewpoint. Utilizing a ligand of medium quality while retaining hydrogen interactions, the nature of interactions occurring within a distance of 4 Å from the molecule was systematically examined. The parameters that governed the definition of hydrogen bonds included distances and angles between designated atoms, adhering to a maximum donor–acceptor (D–A) distance of 3.4 Å. The angular configurations were constrained to values ranging between 90 and 180 deg for various combinations of atoms. Hydrogen bonds involving water molecules were acknowledged when a water molecule acted as a bridge between an HSA atom and a hormone atom that conformed to the established criteria. Additionally, version 1.9.4 of Visual Molecular Dynamics (University of Illinois at Urbana–Champaign) was used to calculate the hydrogen bond occupancy – the percentage of time that a specific hydrogen bond exists throughout each MD simulation-.

### 2.5 Generation of Representative Protein Conformational Clusters

The EnGens (an abbreviation for ensemble generation) methodology for the derivation of representative protein conformations from molecular dynamics simulations is systematically categorized into three principal phases: the initial phase involves the importation of molecular dynamics results, which are assimilated in the format of trajectory files encapsulating the spatial coordinates of the protein atoms at discrete time intervals; the subsequent phase consists of the filtration of molecular dynamics results, entailing the exclusion of incomplete trajectories or those exhibiting errors; and the final phase encompasses the reconstruction of conformational structures, which is executed through the application of an averaging algorithm that computes the mean of the spatial coordinates of the atoms at each discrete time interval.^31^

Following the reconstruction of the conformational structures, the Uniform Manifold Approximation and Projection (UMAP) algorithm reduces the dimensionality of these structures. UMAP is characterized as a nonlinear algorithm that effectively preserves the intrinsic structural relationships among the conformational structures.^32^ Through the dimensionality reduction process, the conformational structures can be represented in a low-dimensional space, thereby enhancing the analysis and interpretation of the resultant data.

The UMAP algorithm successfully delineated four distinct groups of conformational structures for HSA:EST, three for HSA:DHT, and five for HSA:TES, with each group exhibiting significant differentiation from the others. These groups of conformational structures were subsequently analyzed using the Gaussian Mixture Models (GMM) algorithm,^33^ which constitutes a probabilistic machine learning approach predicated on the assumption that the underlying data originates from a mixture of normal distributions. The GMM algorithm was thereafter employed to enhance the initial categorizations made by UMAP. The integration of these algorithms facilitates the identification of conformations that aptly represent the protein’s inherent flexibility.

### 2.6 Molecular Fractionation with Conjugate Caps (MFCC) and Quantum Biochemistry

The investigation proceeded with MFCC calculations pertaining to the representative conformations produced by EnGens, alongside the examination of the final conformation discerned through molecular dynamics. This methodological approach facilitated the assessment of interaction energies among the most significant molecules within the analyzed complexes employing density functional theory (DFT).^34^

The MFCC methodology facilitates an in-depth examination of numerous residues within a protein framework, achieving a reduction in computational cost while preserving precision.^35–37^ In order to confine the analysis to a manageable number of residues without omitting essential interactions, noncovalent interactions within a proximity of 10 Å to the hormone molecules and water molecules within 2.5 Å of each HSA residue and hormone were meticulously evaluated. It is significant to highlight that, owing to the considerable computational expense, only the final conformation of the MD simulations incorporated explicit water molecules. Furthermore, the MFCC and DFT computations conducted for the representative conformations provided by EnGens do not consider explicit solvation effects, but COSMO solvation.^38^ This methodology optimized computational use during the investigation, allowing the execution of calculations within a feasible time frame.

The interaction energy between the HSA residue of interest (*R*_*i*_) and EST/DHT/TES was calculated as follows. First, four auxiliary system were defined: (S1) Ri plus its respective conjugate caps *R*_*i*–1_ and *R*_*i*+1_ (the caps are attached to the amine and carboxyl moieties of *R*_*i*_ and reflect the electronic environment of its neighborhood) and the hormone (EST, DHT, or TES); (S2) *R*_*i*_ plus its conjugate caps only; (S3) the conjugate caps of *R*_*i*_ and EST/DHT/TES; (S4) the conjugate caps of *R*_*i*_ only. For each system, the total energy *E*_Si_ was evaluated using the DFT formalism. All dangling bonds were passivated with hydrogen atoms, and the caps were formed from the immediately adjacent residues of *R*_*i*_ in the protein chain. The interaction energy between EST/DHT/TES and each amino acid residue was given by

1 $$E\left(\mathrm{EST}/\mathrm{DHT}/\mathrm{TES}-R_{i}\right)=E_{S1}-E_{S2}-E_{S3}+E_{S4}$$

DFT computations were conducted to meticulously analyze the components of eq 1 and to elucidate the interaction energies between ligands and proteins. This analysis was executed utilizing the DMol3 computational code^39^ from the Biovia Accelrys Materials Studio software suite. A Double Numerical plus Polarization (DNP) basis set was selected for the expansion of the Kohn–Sham orbitals corresponding to all electrons. The Perdew–Burke–Ernzerhof (PBE) exchange-correlation functional, as delineated within the Generalized Gradient Approximation (GGA) framework,^40^ along with the Tkatchenko-Scheffler dispersion correction methodology (GGA + TS),^41^ were employed to assess the electronic energies. The orbital cutoff radius was established at 4 Å, while the self-consistent field (SCF) convergence criterion was adjusted to a threshold of 10^–6^ Ha. The implementation of water solvation was achieved via the COSMO implicit solvation paradigm,^42^ utilizing a dielectric constant of 40. Ultimately, employing the previously discussed basis set, corrections, orbital cutoff parameters, and SCF modifications, electrostatic potential calculations (isosurfaces) were executed.

### 2.7 Computational Packages

All computational procedures employed in this study were conducted using a combination of established molecular modeling software, web-based platforms, Perl scripts, and a quantum chemistry package, selected to ensure methodological reliability, reproducibility, and compatibility across different stages of the workflow. All packages used in this work are widely used in computational studies and modeling of biological complexes.

Discovery Studio 2016 (Dassault Systèmes) was used to prepare the HSA structure, remove crystallographic water molecules and minor ligands, perform ligand geometry optimization, and analyze noncovalent protein–ligand interactions. The ProteinPrepare package (PlayMolecule AI) was employed to assign protonation states and add hydrogen atoms to HSA at physiological pH (7.4). MarvinSketch 18.24 (ChemAxon) was used to determine steroid’s protonation and charge states. AutoDockTools 1.5.6 was used for docking system preparation, including partial charge assignment and ligand flexibility definition, while AutoDock Vina 1.1.2 was employed to perform blind molecular docking simulations. GROMACS 2023.1 was employed to perform molecular dynamics simulations, including energy minimization, equilibration, and production runs, using the CHARMM36 force field, CGenFF ligand parameters, and the TIP3P water model. Trajectory analyses were performed on the GROMACS analysis packages. The EnGens methodology was used to generate representative protein conformations from molecular dynamics trajectories. UMAP was employed for dimensionality reduction and posterior generation of conformational ensembles, and GMM was used for cluster refinement. DMOL3 package of Materials Studio 8 (Dassault Systèmes) was employed to perform DFT calculations after the schematizing of Ri:Hormone contacts by MFCC, using the PBE exchange–correlation functional with GGA and Tkatchenko–Scheffler dispersion corrections. Both the MFCC and DFT steps were made automatic using Perl scripts.

## 3 Results and Discussion

### 3.1 Molecular Docking Analysis

The sex hormone transportation by HSA is clinically relevant. In this context, studies have demonstrated the efficacy of molecular docking in elucidating the binding mechanisms of various compounds, including hormones, to HSA.^10,11,16^ Additionally, structural studies on steroid hormones have provided insights into how the androgen receptor binds to TES and DHT.^43^ The application of molecular docking extends beyond the scope of HSA, hormones, and androgen receptor studies. This computational technique is widely employed to model the interactions between relevant biomolecules. This method typically consists of two steps: prediction of the binding modes between two molecules and the ranking of these binding modes by using scoring functions.^44,45^ Molecular docking was used to predict the potential binding sites through which EST, DHT, and TES could be transported within HSA.

As depicted in Figure 2, similar docking scores were observed for the binding modes predicted for HSA complexed with the different hormones under study, with the following observations: EST shows a range from −7.0 to −9.2 kcal·mol^–1^, with a mean of −8.3 kcal·mol^–1^ (Figure 2A); DHT shows docking scores ranging from −7.0 to −9.4 kcal·mol^–1^, with a mean of −8.5 kcal·mol^–1^ (Figure 2B); and TES shows docking scores ranging from −7.2 to −9.5 kcal·mol^–1^, with a mean of −8.5 kcal·mol^–1^ (Figure 2C). The observed similarity between the docking scores is consistent with the high structural similarity between these hormones (Figure 1B–1D).

**Figure 2:**
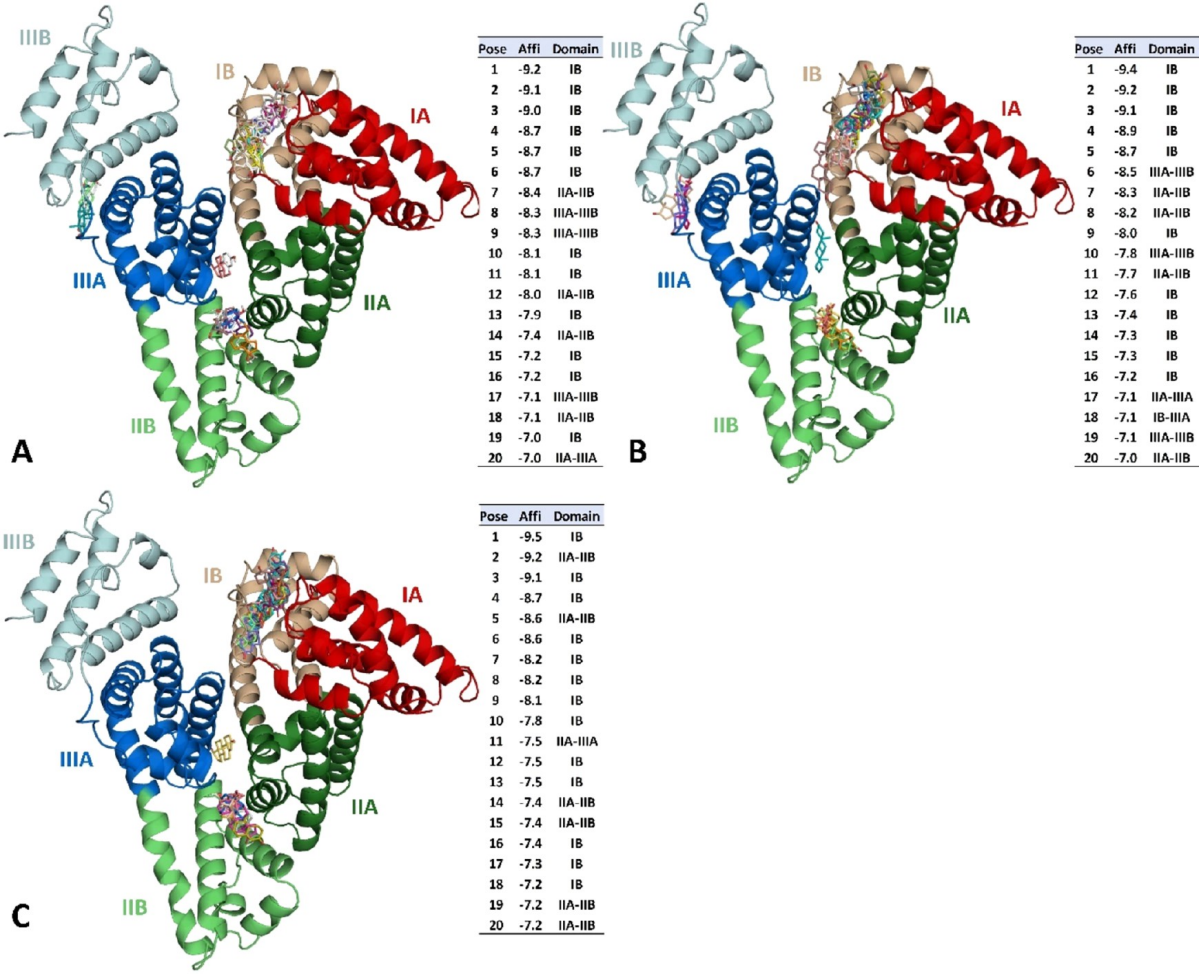
Molecular docking between HSA and EST, DHT, and TES listing the best 20 poses with the lowest docking score (kcal·mol^–1^). The binding profiles of (A) EST, (B) DHT, and (C) TES in HSA are shown in the 3D illustrations and in the tables on the right.

The binding modes of steroid hormones with HSA indicate a binding preference for the binding site located within the IB subdomain, as evidenced by the successful fitting of multiple poses within this site. Specifically, of the 20 poses found in each complex, 12 EST poses (Figure 2A), 11 of DHT (Figure 2B), and 13 of TES (Figure 2C) occupied this site, arising IB subdomain as a possible carrying region for these hormones. A secondary hotspot with four EST poses, four DHT poses, and six TES poses is located between the IIA and IIB subdomains (Figure 2).

Distinct binding sites have been previously proposed as potential carriers of TES, such as TBS1 (located between subdomains IIA and IIB), TBS2 (located between subdomains IA and IB),^9^ FA3, and FA6.^11^ In this context, the molecular docking data obtained in this study prompted a more focused investigation into the behavior of EST/DHT/TES on IB subdomain (FA1) and between IIA and IIB subdomains (FA6). The molecular docking results indicated the IB subdomain as potentially critical for interaction with steroid hormones. It is in total agreement with the molecular model proposed by Danesh and co-workers, which suggests that FA1 serves as the EST carrier. Notably, the second identified hotspot, TBS1 (parallel to FA6), and TBS2 were previously determined to be critical in mediating the binding between equine serum albumin and TES. The amino acids that comprise these binding sites are found to be highly conserved in both Equine serum albumin (ESA) and HSA, including residues that facilitate equivalent interactions. This finding suggests the potential for identical occupation of TES and potentially other hormones in both albumins.^9^ According to Czub et al., TBS1 is more structurally conserved than TBS2 when compared to their equivalent binding pockets in Human Serum Albumin (HSA). This higher conservation of TBS1 may explain why a homologous binding pocket for TBS2 was not identified in the potential binding sites obtained from blind molecular docking assays on HSA.

### 3.2 Molecular Dynamics

Molecular dynamics simulations have been successfully employed in the field of drug discovery, as they facilitate a deeper comprehension of protein flexibility and a temporal evolution of protein–ligand complexes by using Newton’s equations of motion to calculate the movement of their atoms along a simulated time.^46,47^ In view of this, MDs were applied to assess the behavior of EST/DHT/TES in the main prospective sites suggested by molecular docking assays (Figure 2).

The RMSD of the HSA protein without considering ligands fluctuates within a range of approximately 2 to 5 Å during the simulations, except for HSA bound to EST-FA6, which exceeded 6 Å even close to the simulation end. This suggests that HSA maintains its structural stability when bound to DHT and TES occupying FA1 and FA6 binding sites, while presenting higher structural instability when EST is bound to FA6 (Figure 3). Moreover, the structural analysis indicates a transition from the initial binding mode proposed by molecular docking. Despite this initial repositioning, steroid hormones do not fully escape the binding pockets during the molecular dynamics (Supporting Figures S1, S2, and S3). It is worth noting that the distances presented in Supporting Figures S2 and S3 are greater than those reported in our tables, because, for the calculation of distances throughout the simulations, the center of mass of the ligands is considered, whereas in the interaction energy calculation tables, the distances correspond to those between the closest atoms of the ligands and HSA.

**Figure 3:**
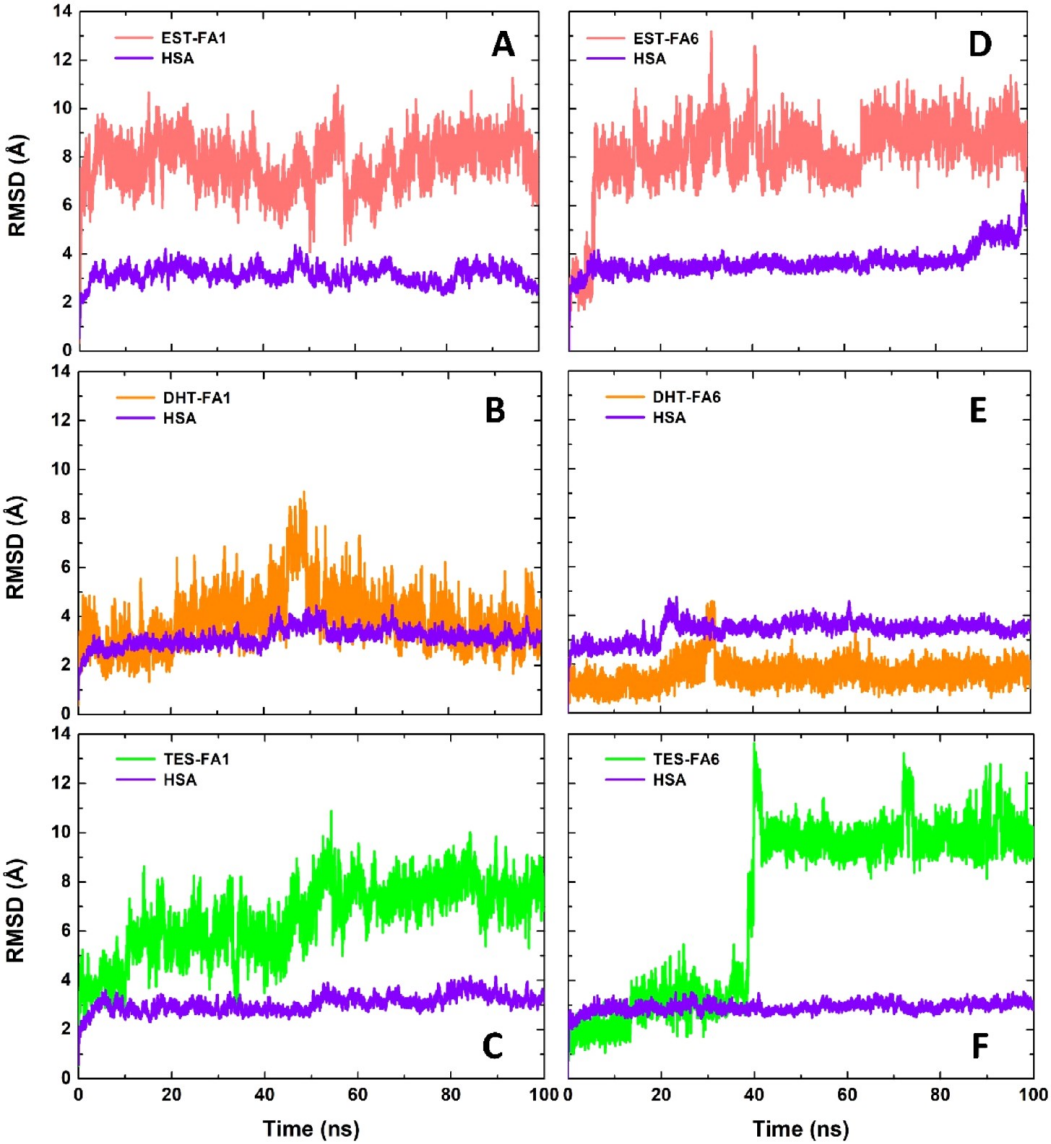
All heavy-atom Root-mean-square deviation as a function of time of the complexes HSA:Hormone. Outcomes of the complexes (A) HSA:EST-FA1, (B) HSA:DHT-FA1, (C) HSA:TES-FA1, (D) HSA:EST-FA6, (E) HSA:DHT-FA6, and (F) HSA:TES-FA6 are presented herein. RMSD data referring to HSA and steroid hormones are shown in purple and salmon/orange/green, respectively.

Except DHT bound to FA6, the hormones exhibited high RMSD variations, with values greater than 8.0 Å (Figure 3), suggesting that these molecules are capable of substantial displacement from their original positions obtained by molecular docking. While DHT demonstrated RMSD values lower than 6 Å in both binding sites, EST and TES exhibited values higher than 6 Å. This finding suggests that the initial conformations predicted by molecular docking to EST and TES were less favorable than those obtained for DHT (Figure 3). These high RMSD values can be explained by the absence of strong and conserved contacts, such as hydrogen bonds, during most of the simulations. The most conserved hydrogen bond interaction (LEU481-FA6:DHT) did not occur for even a quarter of the simulation time (Supporting Figure S4). Although the RMSD and hydrogen bond occupancy data suggests that DHT has a greater propensity to bind to both HSA binding sites than EST and TES, its ΔRMSD (the difference between the highest and lowest RMSD value during a time interval) remains elevated when was bound to FA1, exceeding 4 Å during the final 10 ns of the MD simulation. The DHT complexed in the FA6 binding site showed the lowest ΔRMSD, usually less than 2.0 Å after 40 ns, suggesting a better fit for DHT in this pocket.

The HSA subdomains exhibited similar fluctuations when hormones bound to both FA1 and FA6. Regarding complexes based on FA1 binding, portions of subdomains IB, IIB, and IIIB showed higher fluctuations when HSA bound to TES than to DHT and EST. For HSA complexes based on FA6 anchorage, DHT binding resulted in higher fluctuations in the initial segment of IA and the initial and final segments of IIIB, while TES binding promoted higher fluctuations in the final segment of IA and increased rigidity in IIIB (Supporting Figure S5).

These observations suggest that the complex formed between HSA-FA6 and DHT is the most stable, which is in line with the fact that FA1 is more accessible to solvent.^48^ Although FA6 exhibits an opened cleft that performs transient binds,^48^ which is in agreement with the high fluctuations of EST and TES at this site, DHT presented a good fit in this site. Furthermore, experimental reports that demonstrated the low affinity of HSA for sex hormones are in complete agreement with this high fluctuation,^10,11^ which may also suggest a poor fit between protein and ligand.

### 3.3 Conformational Ensembles of HSA:Hormone Complexes

It should be noted that the biological function of proteins is not fully explained by their three-dimensional structures in a static mode but rather is directly correlated with their dynamic behavior. Therefore, the use of statistical methods to extract representative information about this behavior is critical to understand the stability and binding features of biological complexes,^31,49^ like the HSA:EST/DHT/TES complexes under investigation. As detailed in Section 2.5, multiple conformations of each HSA:Hormone complex were extracted from molecular dynamics trajectories by employing dimensionality reduction and clustering.

The representative conformations of the complexes are depicted in Figure 4 to provide a more visual representation of the differences in hormone occupation across the representative conformations. DHT demonstrated a more compact and rigid binding pattern in both FA1 and FA6 when compared with EST and TES (Figure 4). The binding modes identified on FA1 exhibited greater variability compared to those observed on FA6 (Figure 4). However, the RMSD and the structural evolution of the hormones within FA1 and FA6 reveal that all hormones appear to stabilize after 40 ns of molecular simulations in both pockets (Figures 3 and S1). This finding indicates that most of the binding modes observed after 40 ns are potentially definitive.

**Figure 4:**
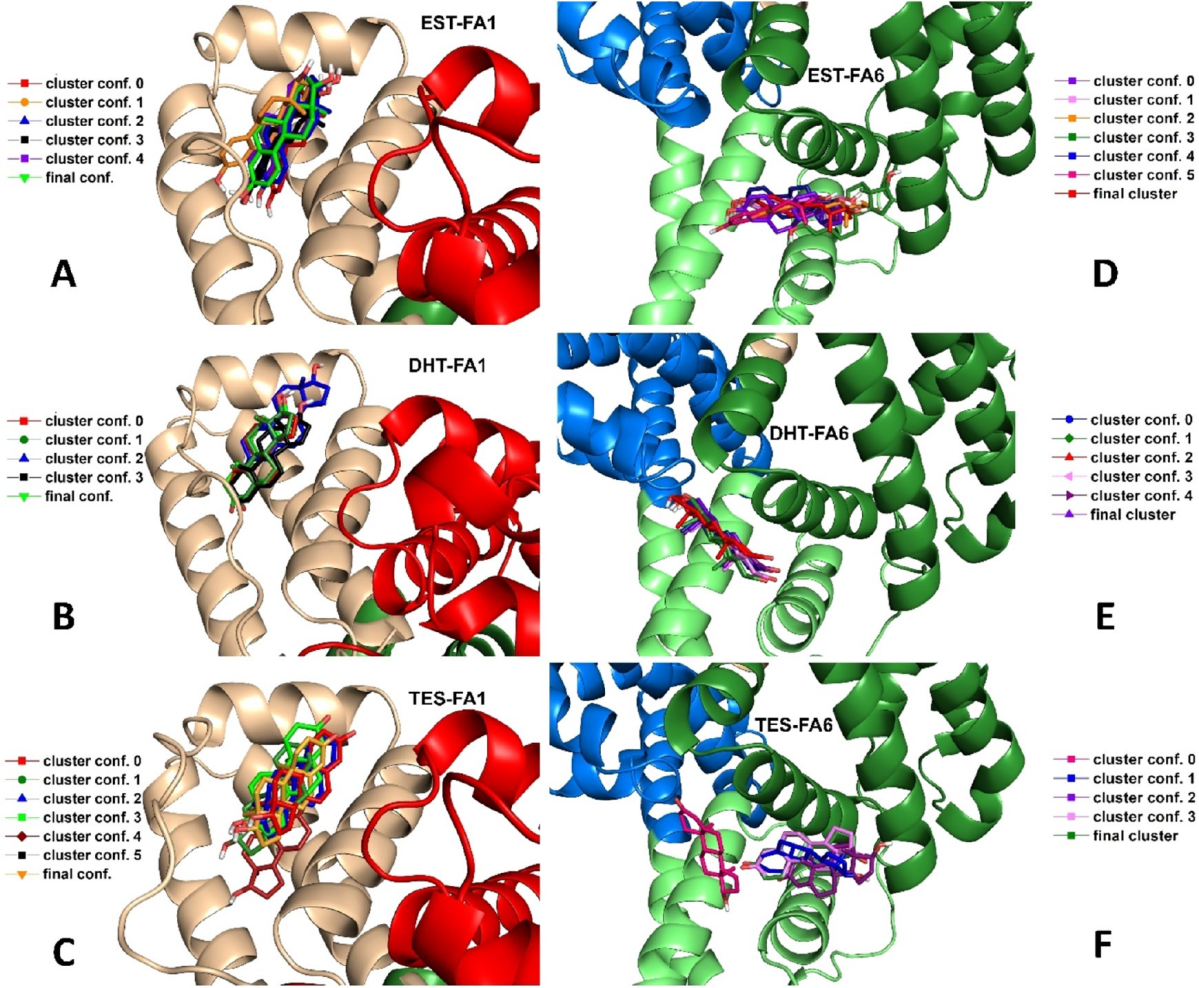
Structural alignment of representative conformations of HSA:Hormone complexes. Multiple conformations of (A) Estradiol (EST), (B) dihydrotestosterone (DHT) and (C) testosterone (TES) bound at FA1 binding site. Conformational states of (D) EST, (E) DHT, and (F) TES interacting with the FA6.

### 3.4 Analysis of Quantum Biochemistry and Atomic Details of the Binding

HSA plays an essential role in binding sex hormones such as EST, DHT, and TES in the blood. Historically, the differential affinity of HSA for these hormones was not entirely understood. The historical use of the MFCC-DFT framework to describe interfacial contacts in protein:protein and protein:ligand complexes, including some HSA:ligand complexes.^51,21,50,51^ establishes the basis for its use to obtain new insights that could help understand HSA:Hormones complexation. The MFCC method, developed by Zhang et al., involves decomposing the protein into individual amino acid residues to calculate their interaction energies with a ligand. This uses molecular caps to reproduce the local atomic environment. The sum of these residue:ligand interactions provides an accurate estimate of the total protein:ligand interaction energy.

In light of this scenario, we employed DFT-based calculations in conjunction with the MFCC scheme to conduct investigation of the nonbonded interactions between these hormones and HSA residues, taking into account a set of representative conformations resulting from molecular dynamics. Although its affinity for these hormones is relatively low, HSA plays an important role in maintaining a fraction of these hormones in free form, thus influencing their bioavailability and physiological activity.^52^

Figures 5 to 11 show a detailed analysis of the interaction energy, binding modes and noncovalent interactions of EST, DHT and TES with HSA in the binding sites FA1 and FA6. The quantum biochemistry demonstrated that residues located more than 5 Å from EST/DHT/TES did not contribute significantly to the stability of these systems, as the convergence of the interaction energy occurred around a radius of 5 Å (Figure 5). Contributions from more distant residues decay rapidly and have little impact on relative energy trends, making this cutoff reliable for qualitative convergence, though not for absolute binding energy estimates.

**Figure 5:**
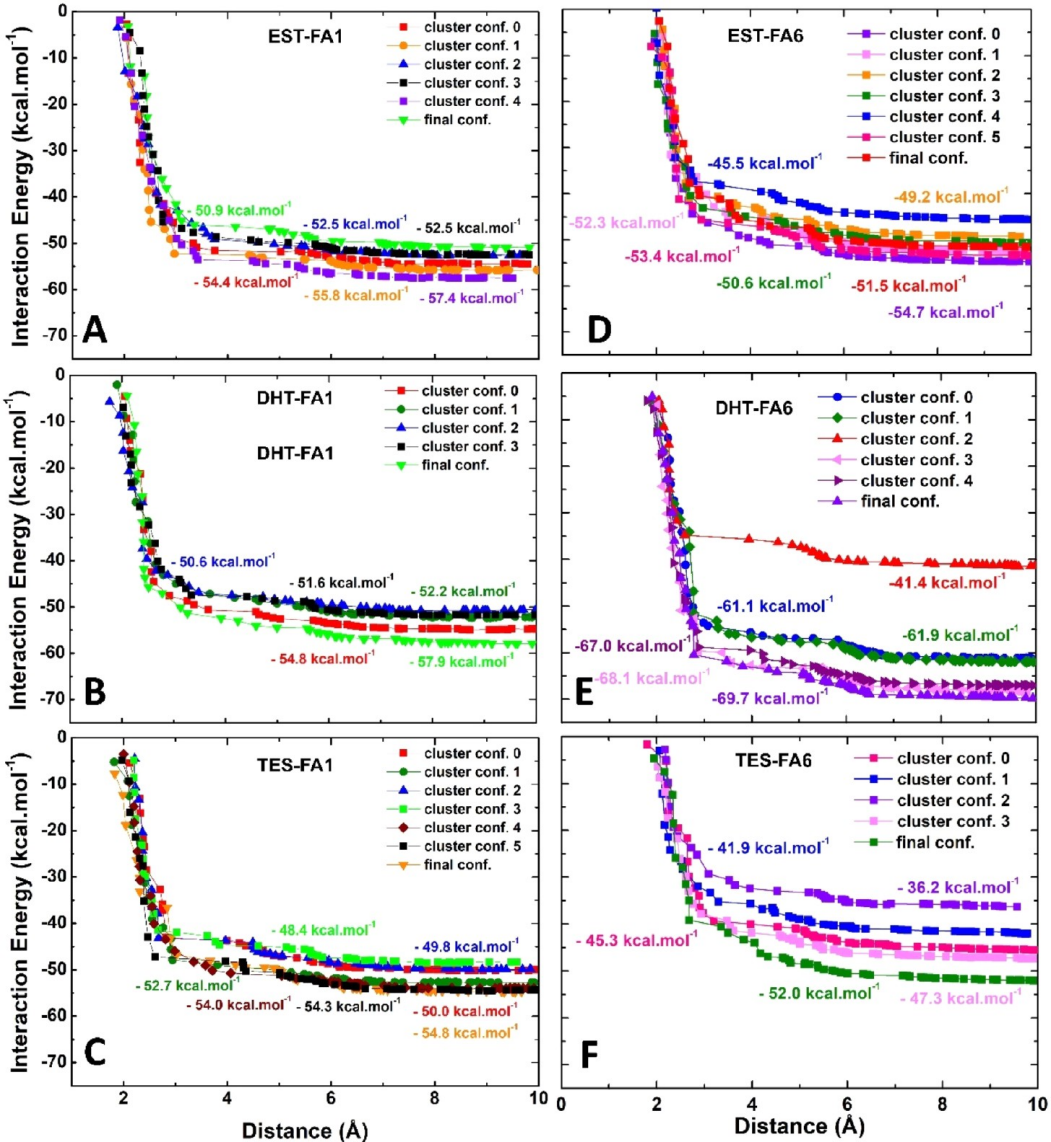
The total interaction energy between steroid hormones and the HSA residues as a function of the binding site radius. Interaction energies of (A) Estradiol (EST), (B) dihydrotestosterone (DHT) and (C) testosterone (TES) complexed at FA1. Graphs (D–F) show the corresponding interaction energy distributions for the same hormones bound at the FA6 site.

Despite the considerable variation in the hormone RMSD values, the total interaction energies of the hormone conformations binding to FA1 are comparable, as evidenced by the low standard deviation and coefficients of variation (Table 1). The interaction energies found in the representative conformations of DHT and TES binding to FA6 have higher standard deviations and coefficients of variation (Table 1). The mean interaction energies between HSA-FA1 (HSA-FA6) and EST, DHT, and TES were −53.9 (−43.4), −53.4 (−61.5), and −52.0 (−44.5) kcal·mol^–1^, respectively (Table 1). Supporting Tables 1–35 provide details on the local interaction energy contributions between individual HSA residues and the steroid hormones, whose total values are summarized in Table 1. While this integrative computational strategy provided a detailed molecular understanding of HSA:Hormone interactions, certain limitations highlight valuable avenues for future research. For example, it was not possible to use rigorous free energy perturbation or thermodynamic integration methods to calculate the absolute or relative binding free energies (Δ*G*) of the hormones binding to HSA, which could indicate favorable binding reactions.

**Table 1:** Interaction Energy of All Simulations, with Mean Value, Standard Deviation (σ) and Coefficient of Variation (C_V_)

	interaction energy kcal·mol^–1^							mean kcal·mol^–1^	σ kcal·mol^–1^	C_V_
EST ^FA1^	–50.9	–52.5	–52.5	–54.4	–55.8	–57.5		–53.9	2.23	4.1%
DHT ^FA1^	–50.6	–51.6	–52.2	–54.8	–57.9			–53.4	2.64	4.9%
TES ^FA1^	–48.4	–49.8	–50.0	–52.7	–54.0	–54.3	–54.8	–52.0	2.37	4.6%
EST ^FA6^	–45.5	–49.2	–52.3	–53.4	–50.6	–51.5	–54.7	–43.4	2.80	5.5%
DHT ^FA6^	–41.4	–61.1	–61.9	–67.0	–68.1	–69.7		–61.5	10.44	16.9%
TES F^A6^	–36.2	–41.9	–45.3	–47.3	–52.0			–44.5	5.92	13.30%

While these results suggest that the IB subdomain exhibits comparable binding capacities for the three hormones studied, EST demonstrates a slightly stronger interaction with HSA-FA1 than HSA-FA6 (Table 1). This finding lends further credence to the hypothesis that EST exhibits a more stable and predictable interaction with HSA-IB subdomain. This finding is consistent with data previously reported, which indicated that EST exhibits a binding percentage of 78% to HSA, while TES demonstrates a binding percentage of only 50%.^52^ Steroids hormones are characterized by a core composed of four aromatic rings. Therefore, it was also hypothesized that EST, DHT, and TES would bind to the FA1 site, as other aromatic molecules, such as indomethacin and triiodo benzoic acid, have demonstrated affinity for this binding site and for FA7.^48^ This may have substantial consequences for the bioavailability and physiological efficacy of the hormone in circulation, as well as other exogenous compounds that bind to FA1, such as fusidic acid,^53^ salicylic acid, zidovudine,^54^ and neonicotinoids.^55^

Quantum calculations indicate that, in contrast to the theoretically observed interaction energy at the FA1 site, FA6 exhibits a stronger propensity to bind DHT (Table 1). These data suggest that this site can be suitable for carrying hormones and is consistent with a crystallographic structure of equine serum albumin bound to TES through a conserved binding site located in a homologous position of FA6, named TBS1.^9^ It was determined that EST and DHT exhibited comparable and elevated theoretical affinities for HSA-FA6 in comparison to TES. Consequently, it is hypothesized that FA6 occupation by these compounds is deemed to be significant. Halothane, diflunisal, and ibuprofen are examples of FA6 binders that have the potential to result in competition/displacement and to affect hormone bioavailability, with unknown consequences. A previous study, which employed a remarkably similar approach to MFCC and DFT integration, revealed the HSA-FA6:Ibuprofen crystallographic structure has a total interaction energy of −52.2 kcal·mol^–1^,^51^ which is less attractive than the HSA-FA1:DHT here calculated. This finding serves to reinforce the hypothesis that the FA6 may exhibit a predilection for DHT, which has a mean interaction energy of −61.5 kcal·mol^–1^.

Compared to the SHBG:EST/DHT/TES complexes analyzed using an identical protocol,^56^ the HSA complexes exhibit notably weaker theoretical affinities. Depending on the hormone and binding pocket, the interaction energy gap ranges from ∼11 to 27 kcal_·_mol^–1^. This finding aligns with prior experimental studies demonstrating that HSA has a lower binding affinity for steroid hormones than SHBG.^57^ However, the possibility of steroid hormones occupying multiple HSA binding sites, such as FA1, FA6, and others previously proposed^9–11^ , combined with the significantly higher amount of HSA in serum when compared to SHBG, can explain why up to 54% of TES is carried by this protein in the circulating plasma.^11^

#### 3.4.1 Interactions HSA-FA1:EST

Figure 6 illustrates the interaction between EST and HSA-FA1 residues in two different conformations: “EST final conf.” and “EST cluster conf. 4”, corresponding the conformation with the highest interaction energy with a total energy of −50.9 kcal·mol^–1^ (Figure 6A,5B) and the lowest interaction energy with −57.4 kcal·mol^–1^ (Figure 6C,6D). Furthermore, these interactions occur in different regions of EST, identified as regions I, II, and III (Figure 1B). While region I of EST does not appear to be critical to the binding mode represented in the final conformation (Figure 6A), this portion mediates key contacts in conf. Four (Figure 6C).

**Figure 6:**
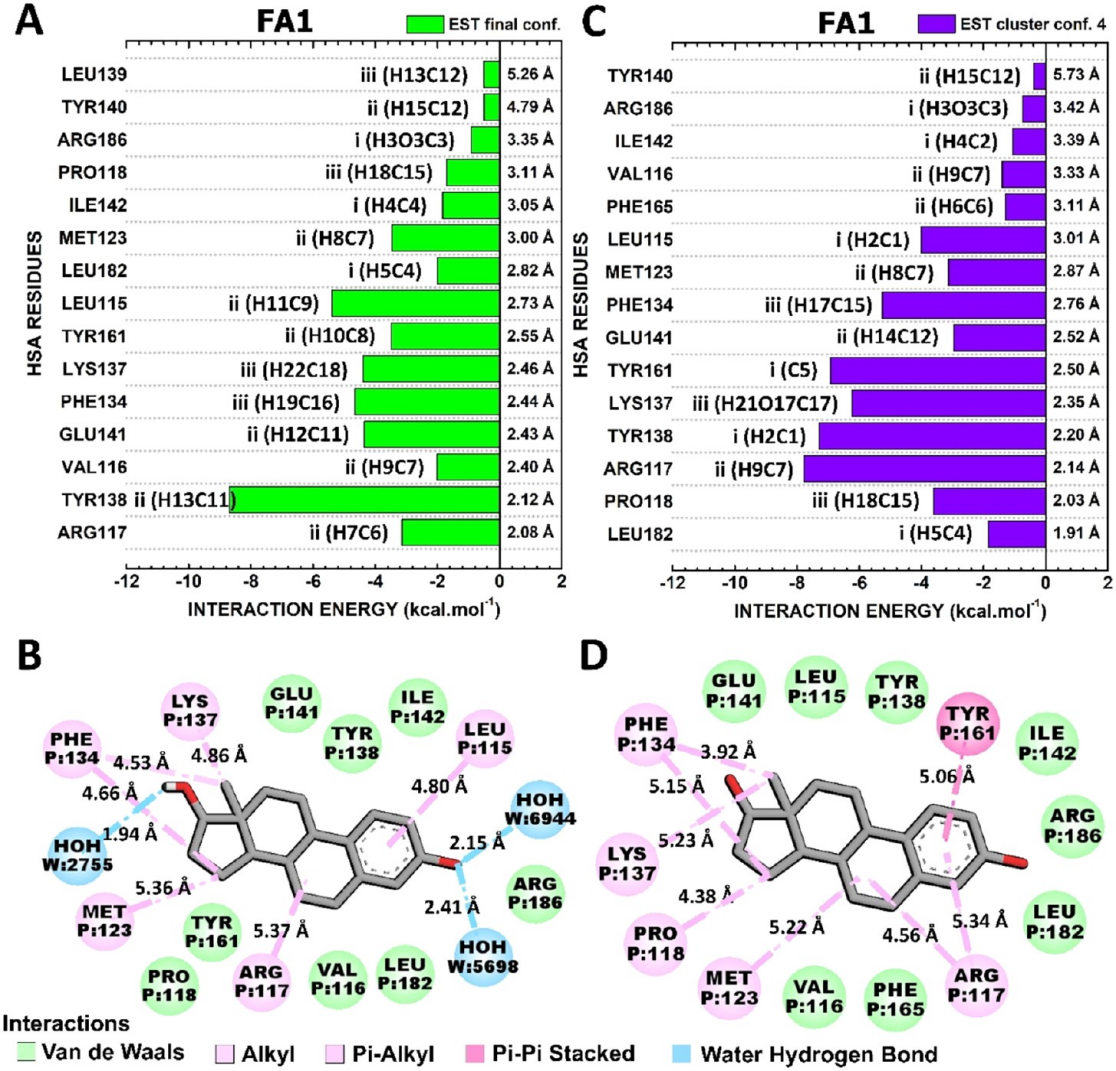
Quantum biochemistry results for the HSA-FA1:EST complex. Binding site, Interaction energy, and Residues Domain (BIRD) panel shows the interaction energy of each residue with the regions of the molecule for the (A) highest and (C) lowest energy of interaction clusters. On the left of each bar, the interactive regions of the EST are labeled with Roman numerals (i–iii), which were previously identified in Figure 1. Noncovalent interactions between EST and HSA-FA1 in (B) EST final conf. and (D) EST cluster conf. Four are illustrated.

The residues with the highest energetic contributions in “EST final conf.” include TYR138 (−8.69 kcal·mol^–1^), LEU115 (−5.38 kcal·mol^–1^), PHE134 (−4.65 kcal·mol^–1^), LYS137 (−4.38 kcal·mol^–1^), MET123 (−3.47 kcal·mol^–1^), and TYR161 (−3.49 kcal·mol^–1^) (Figure 6A). While TYR138 and TYR161 mediate these contacts through van der Waals interactions, LEU115, MET123, PHE134, and LYS137 established hydrophobic contacts (Figure 6B).

The conformation that exhibited the lowest interaction energy shares numerous contacts with the previously described final conformation. However, with notable variations in the value of the interaction energy: ARG117 (−7.78 kcal·mol^–1^), TYR138 (−7.28 kcal·mol^–1^), LYS137 (−6.24 kcal·mol^–1^), TYR161 (−6.92 kcal·mol^–1^), PHE134 (−5.25 kcal·mol^–1^), and LEU115 (−4.00 kcal·mol^–1^) (Figure 6C). LEU115 and TYR138 established van der Waals interactions. Additionally, ARG117, PHE134, LYS137, and TYR161 were found to be involved in hydrophobic interactions (Figure 6D).

Comparing the two conformations, the ″EST cluster conf. 4″ involves stronger interactions with critical residues such as ARG117, LYS137, TYR138, and TYR161. These stronger contacts, in particular the hydrophobic alkyl and π-alkyl interactions, are probably responsible for the greater stability observed in this conformation, reflected in the interaction energy of −57.4 kcal·mol^–1^. On the other hand, the ″EST final conf.″ conformation has more distributed interactions and lower energies, especially with residues such as LEU115 and PHE134. Although this configuration is energetically less favorable, it still maintains significant interactions, especially with hydrophobic residues. These conformations above exhibit notable parallels with the model proposed by Danesh and co-workers through molecular docking, including the residues LEU115, VAL116, ARG117, and VAL182, which are identified as being critical for the attachment of EST in both models. Furthermore, quantum biochemistry indicates new key residues responsible for maintaining EST in this cleft, such as PHE134, LYS137, TYR138, GLU141, and TYR161.

The conformation with lowest interaction energy is favored by stronger interactions with specific residues, which provides additional stability to the bond between EST and HSA. The importance of hydrophobic interactions detected in this conformation is in line with the positive changes in binding entropy of HSA:EST which suggested hydrophobic interactions as the most critical for this complex.^10^

#### 3.4.2 Interactions HSA-FA6:EST

The interactions of the HSA-FA6:EST conformations of highest and lowest energy of interaction, “EST cluster conf. 4” and “EST cluster conf. 0”, with total interaction energies of −45.5 and −54.7 kcal·mol^–1^, respectively, are deeply explored in Figure 7. Interestingly, portions II and III of EST are responsible for most of the critical contacts in both conformations, except for the conserved interaction with ALA213, which is mediated for the C1 contained in portion I (Figure 7).

**Figure 7:**
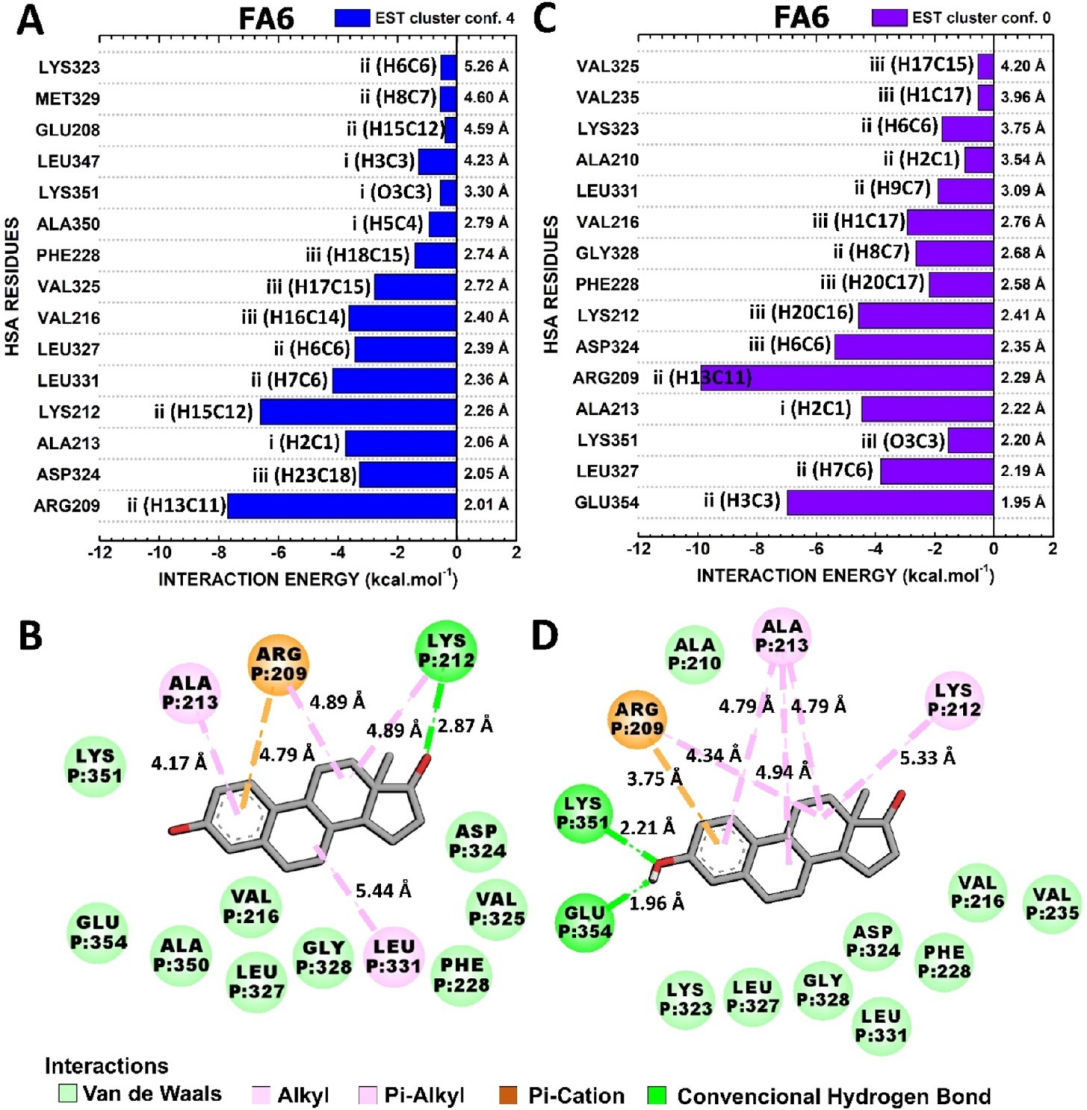
Quantum biochemistry results for the HSA-FA6:EST complex. BIRD panel shows the interaction energy of each residue with the regions of the molecule for the (A) lowest (EST cluster conf. 4) and (C) highest (EST cluster conf. 0) theoretical energy of interaction clusters. On the left of each bar, the interactive regions of the EST are labeled with Roman numerals (i–iii), which were previously identified in Figure 1. Noncovalent interactions between EST and HSA-FA6 in (B) EST cluster conf. Four and (D) EST cluster conf. 0 are shown.

The residues with the strongest interactions with the hormone in the EST cluster conf. Four were ARG209, LYS212, LEU331, ALA213, VAL216, and LEU327, which had interaction energies of −7.69, −6.60, −4.18, −3.74, −3.63, and −3.44 kcal·mol^–1^ (Figure 7A). VAL216 and LEU327 were responsible for van der Waals interactions. ARG209, LYS212, ALA213, and LEU331 established hydrophobic interactions. ARG209 and LYS212 also performed a π-cation contact and a hydrogen bond with EST, respectively (Figure 7B).

The critical residues responsible for the stabilization of EST in the conformation of lowest interaction energy were ARG209, GLU354, ASP324, LYS212, ALA213, and LEU327 with energies of −9.90, −6.97, −5.38, −4.58, −4.45, and −3.82 kcal·mol^–1^ (Figure 7C). These strong contacts are principally mediated by hydrophobic contacts (ARG209, LYS212, and ALA213), van der Waals interactions (ASP324 and LEU327), a π-cation interaction (ARG209) and a hydrogen bond (GLU354) (Figure 7D).

Previous experiments have demonstrated that hydrogen bonds and van der Waals forces are the predominant forces responsible for maintaining HSA bound to EST.^10^ Consistent with these findings, our observations revealed hydrogen bonds facilitating the interaction of EST at FA6, whereas no hydrogen bonds were detected at FA1. This finding supports the hypothesis that FA6 may serve as a specific binding site for EST, in agreement with previous fluorescence spectroscopic analyses that identified FA6 as the binding site of EST in HSA.^10^

Despite the lower energy of interaction of EST for FA1, its binding specificities to FA6 are analogous to those facilitated by compounds that are known to occupy FA6. These interactions include those with LYS351 side chains and residues located at the entrance of drug site I,^58^ exemplified by ARG209, which showed conserved contacts and the major energetic contribution in both representative conformations scrutinized. Furthermore, it is hypothesized that the pharmacokinetics of drugs which bind to FA1, FA6, and FA7 (drug site I) may be particularly influenced during periods of heightened EST levels in women, such as adolescence, pregnancy, and perimenopause.^59^

#### 3.4.3 Interactions HSA-FA1:DHT

Similarly to the interaction between the EST and HSA binding sites, the conformations ″DHT cluster conf. 2″ and ″DHT final conf.″, which exhibit the highest and lowest total interaction energies of −50.6 and −57.9 kcal·mol^–1^ (Figure 5), were scrutinized. Notably, DHT’s portion III established two critical contacts with ASN130 and PHE134 in ″DHT cluster conf. 2″ that were not conserved in ″DHT final conf.″ (Figure 8). Despite the binding site occupied by DHT being nearly identical in both conformations, with 13 residues in common, the way these contacts occur is not conserved. This finding aligns with the high fluctuation of DHT during molecular dynamics (Figure 3B).

**Figure 8:**
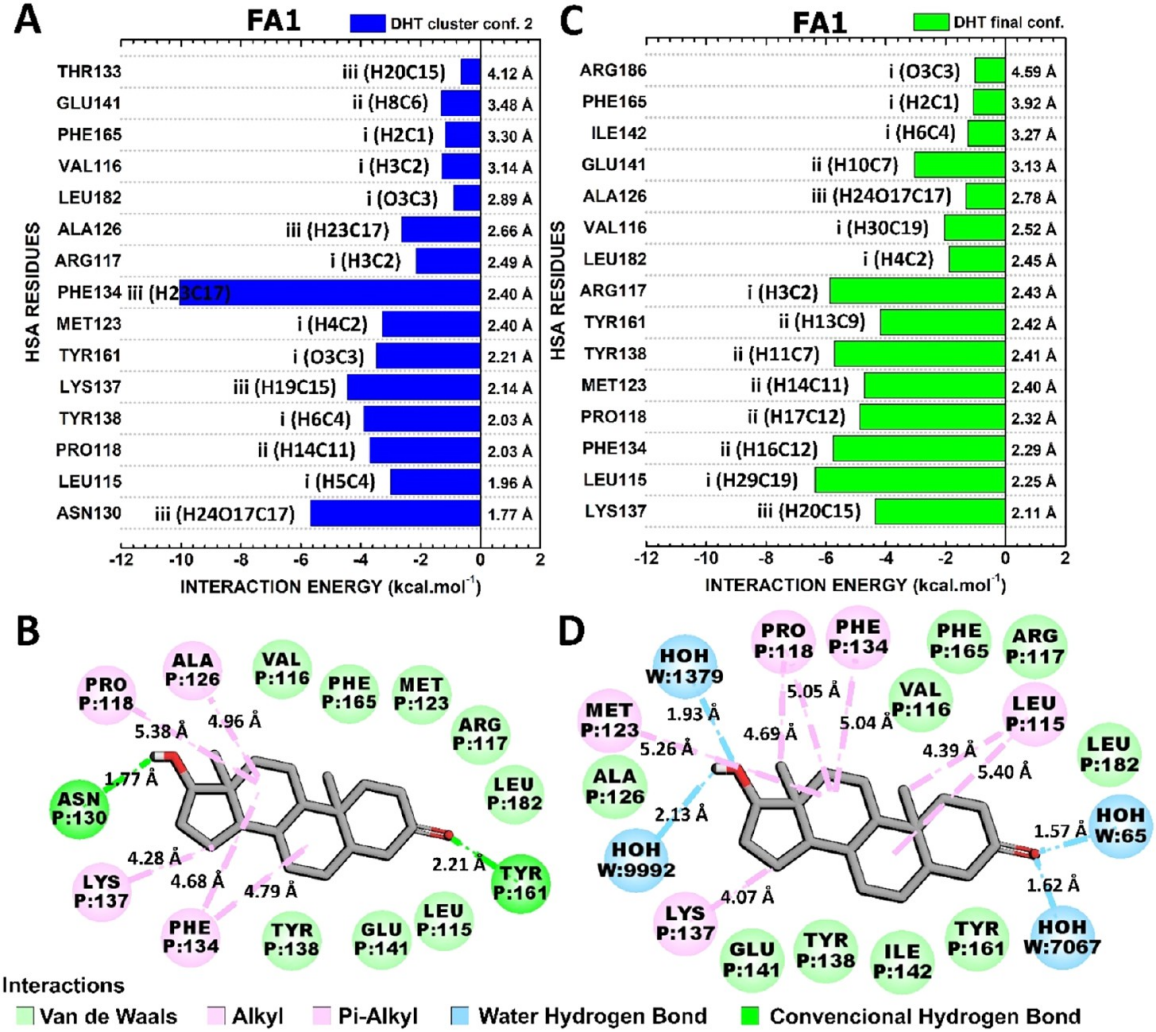
Quantum biochemistry results for the HSA-FA1:DHT complex. BIRD panel shows the interaction energy of each residue with the regions of the molecule for the (A) lowest and (C) highest theoretical energy of interaction clusters. On the left of each bar, the interactive regions of the DHT are labeled with Roman numerals (i–iii), which were previously identified in Figure 1. Noncovalent interactions between DHT and HSA-FA1 in (B) DHT cluster conf. Two and (D) DHT final conf. are shown.

The residues with the highest energetic contributions to the “DHT cluster conf. 2” were PHE134 (−10.03 kcal·mol^–1^), ASN130 (−5.68 kcal·mol^–1^), LYS137 (−4.43 kcal·mol^–1^), TYR138 (−3.88 kcal·mol^–1^), PRO118 (−3.70 kcal·mol^–1^), and TYR161 (−3.48 kcal·mol^–1^) (Figure 8A). While PRO118, PHE134, and LYS137 mediated their contacts mainly through hydrophobic interactions; ASN130 and TYR161 established hydrogen bonds with portions III and I of DHT, and TYR138 performed its strong contact through van der Waals interactions (Figure 8B).

With regard to the ″DHT final conf.″, LEU115, ARG117, PHE134, TYR138, PRO118, and MET123 were identified as the most critical amino acids for the HSA:DHT complex. These HSA:DHT contacts exhibited interaction energies of −6.36, −5.86, −5.76, −5.71, −4.87, and −4.70 kcal mol^–1^, respectively (Figure 8C). LEU115, PRO118, MET123, and PHE134 mediated these energetic contacts through hydrophobic interactions. Additionally, ARG117 and TYR138 established van der Waals interactions (Figure 8D).

The binding pocket of FA1 bound to DHT exhibits a high degree of similarity to that of EST and TES, which include amino acid residues that mediate numerous identical interactions in distinct representative conformations. However, concerning the FA1-based complexes, hydrogen bonds were found exclusively in the DHT ″cluster conf. 2.″ This observation can be associated with the lower level of structural variation observed in the HSA-FA1:DHT complex when compared to HSA-FA1:EST and HSA-FA1:TES (Figure 3).

#### 3.4.4 Interactions HSA-FA6:DHT

The residue:hormone contacts which mediated the interactions of the representative conformations of highest (“DHT cluster conf. 2”) and lowest (“DHT final conf.”) total interaction energies are illustrated in Figure 9. In both conformations, the DHT portions II and III usually mediated more attractive contacts, while potion I contributed in a lower level to the binding at the FA6 binding site.

**Figure 9:**
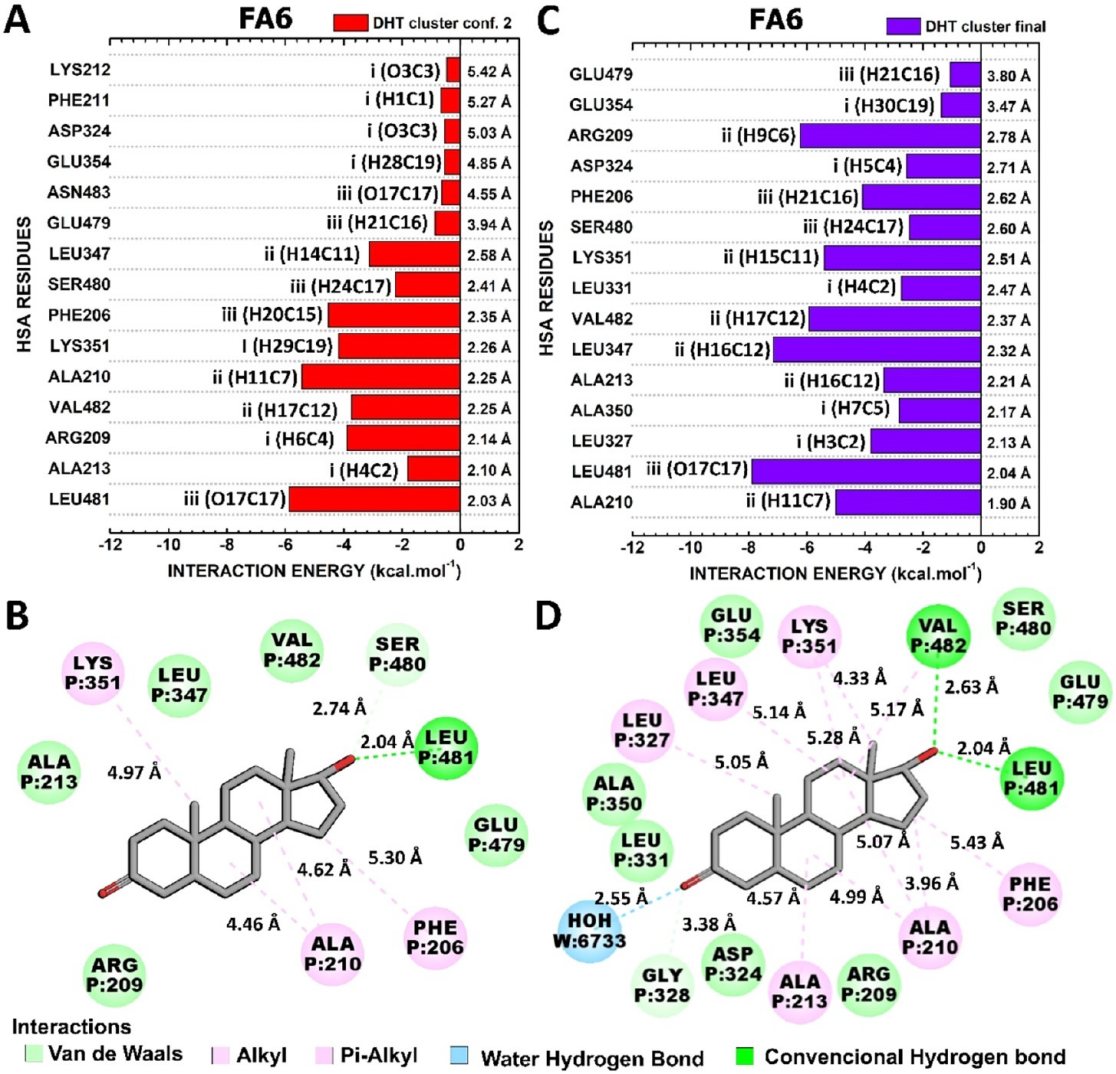
Quantum biochemistry results for the HSA-FA6:DHT complex. BIRD panel shows the interaction energy of each residue with the regions of the molecule for the (A) lowest and (C) highest theoretical interaction energy clusters. On the left of each bar, the interactive regions of the DHT are labeled with Roman numerals (i–iii), which were previously identified in Figure 1. Noncovalent interactions between DHT and HSA-FA6 in (B) DHT cluster conf. Two and (D) DHT final conf. are shown.

LEU481, ALA210, PHE206, LYS351, ARG209, and VAL482 were identified as the residues with the strongest energetic contributions in the HSA-FA6:DHT conformation, which exhibited the lowest total interaction energy (Figure 9A). The interaction energies of these residues are −5.88, −5.43, −4.52, −4.18, −3.90, and −3.73 kcal mol^–1^, respectively. PHE206, ALA210, and LYS351 mediated hydrophobic interactions. Furthermore, ARG209 and VAL482 established van der Waals contacts. Additionally, LEU481 was found to establish a hydrogen bond with DHT (Figure 9B).

The most attractive contacts in the conformation of lowest interaction energy were established by LEU481, LEU347, ARG209, VAL482, LYS351, and ALA210. They were responsible for interaction energies of −7.89, −7.15, −6.24, −5.92, −5.39, and −5.01 kcal·mol^–1^ (Figure 9C). Regarding these main contacts, LEU481 and VAL482 established hydrogen bonds with DHT. ALA210, LEU347, and LYS351 performed hydrophobic interactions. In addition, ARG209 established a van der Waals contact with EST (Figure 9D).

The comparison between these conformations revealed the unique hydrogen bond conserved in both the conformations of lowest and highest theoretical affinities: LEU481­(HN-main chain):DHT­(O17). This hydrogen bond had an occupancy of 24.33% (Supporting Figure S4). This occurrence was not observed in the other complexes, and it is in complete agreement with the molecular dynamics data, which indicated HSA-FA6:DHT as the most stable complex. Although the levels of DHT circulating in the blood are less expressive than those of TES, they are nevertheless clinically significant.^60^ The present study hypothesizes that the levels of endogenous and exogenous compounds carried by FA1 and FA6 can be affected by abnormal steroid hormone levels, which are common in cases of metabolic syndrome, type 2 diabetes mellitus, hypogonadism, 5-α reductase deficiency, and in individuals taking T replacement therapy.^60^ Since quantum biochemistry data suggested that HSA-FA6:DHT occupation is more probable than HSA-FA1:DHT (Figure 5B,E), the molecules carried in this binding site should be more affected than those carried by FA1.

Despite the absence of experimental data concerning the binding between HSA and DHT, the presence of DHT in serum,^49^ and its structural similarity to steroid hormones (Figure 1B–1D), which have been proven to bind to HSA, corroborates the HSA:DHT complexation. It is noteworthy that HSA-FA6:DHT was the complex with the lowest mean interaction energy, indicating a potentially superior theoretical binding affinity in comparison to HSA-FA1:DHT. In addition to corroborating the interaction between DHT and HSA, this theoretical result suggests that DHT has the potential to occupy FA6 with a lower interaction energy than EST and TES. This may result in a higher degree of changes in the pharmacokinetics of compounds bound to FA6, including diflunisal, ibuprofen and myristic acid.^48^ The modeling suggesting that FA6 is a stable and specific binding site for DHT may reveals a novel point of potential competition with FA6’s binders.

#### 3.4.5 Interactions HSA-FA1:TES

Exploring the conformations of highest and lowest energy of interaction of HSA:TES, “TES cluster conf. 3” and “TES final conf.”, were observed total interaction energies of −48.4 and −54.8 kcal·mol^–1^ (Figure 5). The binding site occupied by TES is very similar in both conformations with at least 13 residues in common between them (Figure 10).

**Figure 10:**
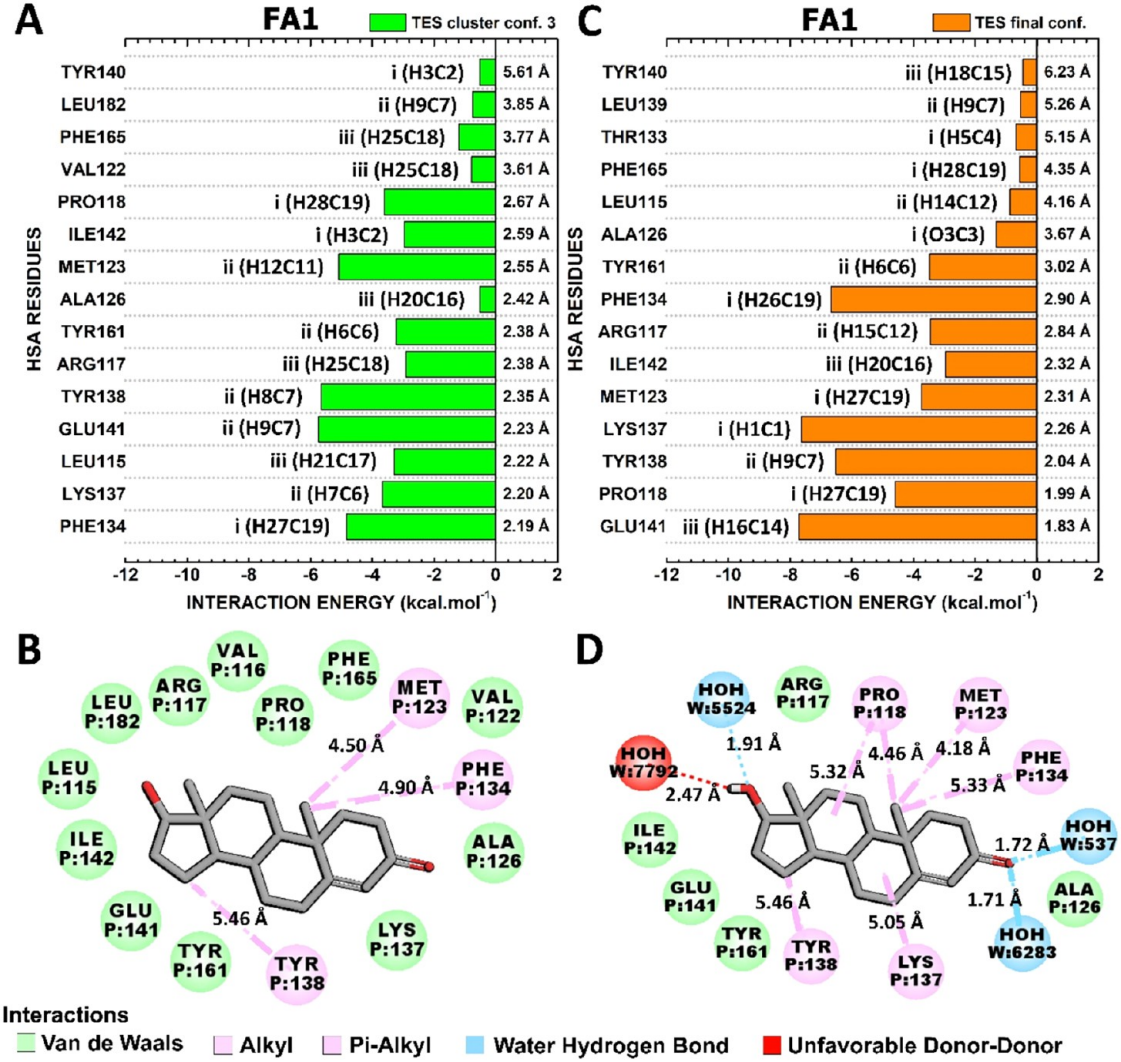
Quantum biochemistry results for the HSA-FA1:TES complex. BIRD panel shows the interaction energy of each residue with the regions of the molecule for the (A) lowest and (C) highest theoretical energy of interaction clusters. On the left of each bar, the interactive regions of the TES are labeled with Roman numerals (i–iii), which were previously identified in Figure 1. Noncovalent interactions between TES and HSA-FA1 in (B) TES cluster conf. Three and (D) TES final conf. are shown.

The main residues responsible for critical interactions with TES in “TES cluster conf. 3” were GLU141 (5.73 kcal·mol^–1^), TYR138 (−5.65 kcal·mol^–1^), MET123 (−5.08 kcal·mol^–1^), PHE134 (−4.84 kcal·mol^–1^), LYS137 (−3.66 kcal·mol^–1^), and PRO118 (−3.61 kcal·mol^–1^) (Figure 10A). The predominant noncovalent interactions established were van der Waals interactions involving PRO118, LYS137, and GLU141, as well as hydrophobic contacts involving MET123, PHE134, and TYR138 (Figure 10B).

The critical residues that stabilize the conformation ″TES final conf.″ are GLU141, LYS137, PHE134, TYR138, PRO118, and MET123, with interaction energies of −7.72, −7.63, −6.68, −6.50, −4.58, and −3.75 kcal mol^–1^, respectively (Figure 10C). Except for GLU141, which established van der Waals interactions with TES, the critical residues performed hydrophobic interactions (Figure 10D).

The crystallographic structure of equine serum albumin binding to TES was determined to reveal two distinct binding sites: one located between subdomains IIA and IIB and the other between subdomains IA and IB. Since these binding sites are conserved in HSA, it was hypothesized that TES attaches these binding sites.^9^ However, data derived from two-dimensional nuclear magnetic resonance spectroscopy has indicated that FA3 and FA6 are the binding sites for TES in HSA.^11^ Consequently, it was hypothesized that TES can bind to distinct regions of HSA, including the FA1 binding site, which is proposed to be a binding site of high degree of energy of interaction for TES.

#### 3.4.6 Interactions HSA-FA6:TES

“TES cluster conf. 2” and “TES final conf” were the conformations with lowest and highest theoretical interaction energies within the representative conformations of HSA:TES-FA6 complex and the molecular description of its critical contacts responsible for the TES occupation in FA6 are illustrated in Figure 11.

The residues of FA6 with highest contribution to the interaction with TES in the conf. Two were ARG209, LYS212, PHE228, ASP324, VAL325, and VAL216, which presented interaction energies of −4.92, −4.32, −4.08, −2.82, −2.56, and −2.54 kcal·mol^–1^ (Figure 11A). Except for LYS212, which mediated hydrophobic contact, these critical residues were involved in van der Waals (Figure 11B).

**Figure 11:**
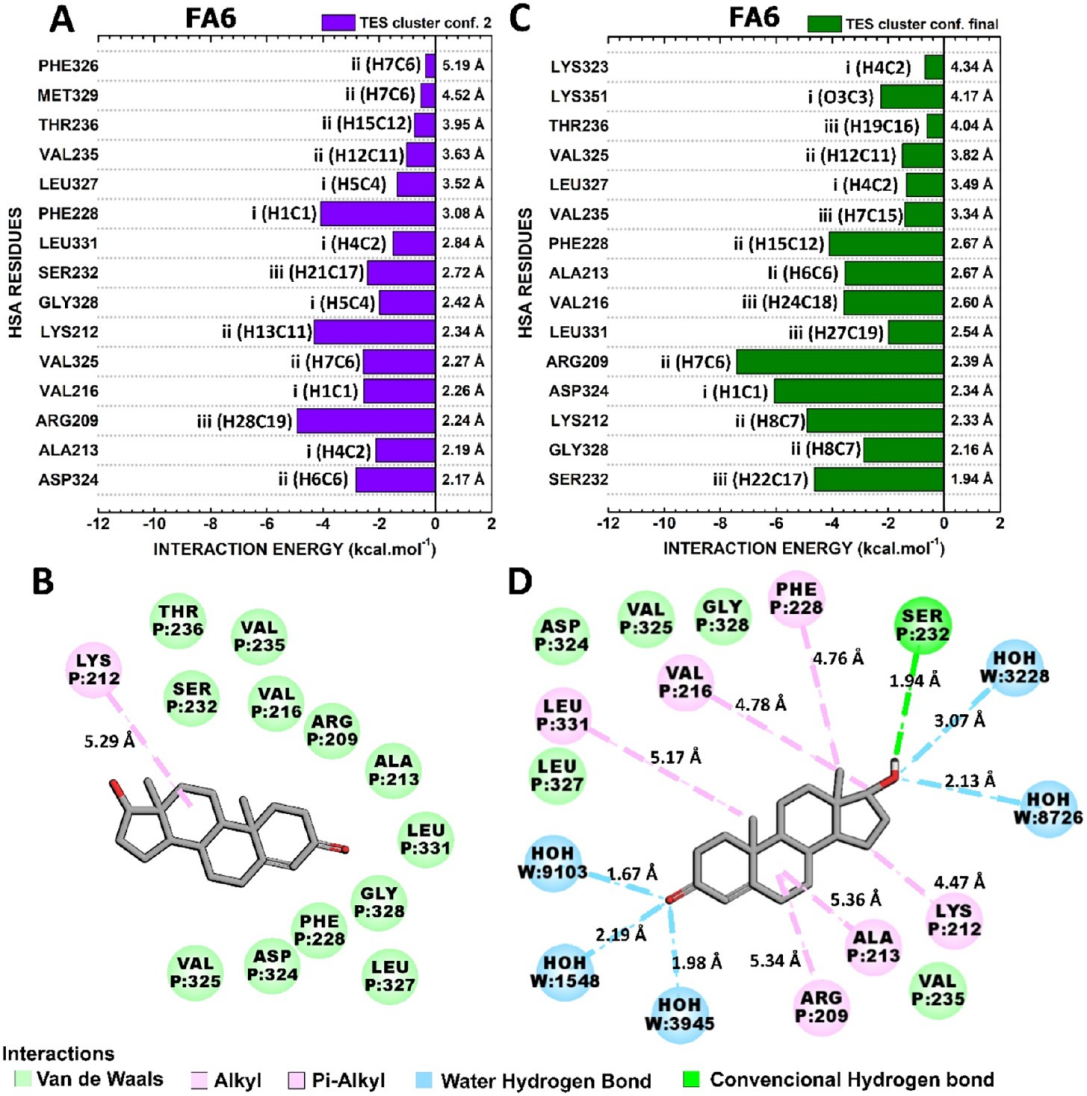
Quantum biochemistry results for the HSA-FA6:TES complex. BIRD panel shows the interaction energy of each residue with the regions of the molecule for the (A) lowest and (C) highest theoretical interaction energy clusters. On the left of each bar, the interactive regions of the TES are labeled with Roman numerals (i–iii), which were previously identified in Figure 1. Noncovalent interactions between TES and HSA-FA6 in (B) TES cluster conf. Two and (D) TES final conf. are shown.

ARG209, ASP324, LYS212, SER232, PHE228, and VAL216 were the residues more responsible for the interaction in the HSA-FA6:TES final confirmation of this complex with interaction energies of −7.41, −6.07, −4.91, −4.63, −4.11, and −3.58 kcal·mol^–1^ (Figure 11C). While ARG209, LYS212, VAL216, and PHE228 mediated hydrophobic contacts, SER232 and ASP324 established a hydrogen bond and a van der Waals contact, respectively (Figure 11D).

The theoretical data on the binding of TES to HSA are consistent with a previous report based on competitive assays. That study indicated that TES displaces oleic acid from HSA binding sites FA6 and FA3, but no displacement was observed at FA1. Moreover, it was reported that HSA possesses both a high- and a low-affinity binding site for TES.^11^ Although this experimental study did not identify FA1 as a TES binding site, our analysis revealed two potential sites: FA1 (with lower interaction energy) and FA6 (with higher interaction energy).

The mean interaction energies of HSA-FA1:TES and HSA-FA6:TES were very similar to those of HSA-FA1:EST and HSA-FA6:EST, respectively. This suggests that these steroid hormones can compete for these binding sites in similar ways. Although previous reports did not explore HSA-FA1:TES complexation, the conservation between ESA and HSA corroborates the occupation of TES within HSA-FA6 because this hormone was found to interact at the FA6 analogous binding site in the ESA.^9^ Furthermore, the variation (the highest value subtracted from the lowest value in module) of mean interaction energies of each binding site (FA1 and FA6) were found to be 10.5, 8.1, and 7.5 kcal·mol^–1^ for EST, DHT, and TES, respectively. This finding suggests a minor variation in the interaction energy of steroid hormones within these binding sites.

##### 3.5 Experimental Data Parallels

Previous experimental reports have reported values of association constants for HSA binding to steroid hormones. Södergard et al. employed equilibrium partition and obtained association constants of 4.21·10^4^ M^–1^, 6.61·10^4^ M_,_^–1^ and 4.06·10^4^ M^–1^ to complexes HSA:EST, HSA:DHT, and HSA:TES at 37 °C, respectively.^61^ Burke and Anderson employed gel filtration to investigate the binding affinity between HSA and EST, yielding association constants of 5.1–6.0·10^4^ M^–1^ and 3.6–3.7·10^4^ M^–1^.^62^ Despite the inherent challenges in comparing association constants calculated based on data obtained through different experimental methods, these data suggest the following decreasing order of affinity for HSA: DHT > EST > TES. It is notable that the existing literature offers a more extensive array of associations constants for these complexes. However, these constants differ from the previously cited ones, as they are described in separate works.

The quantum calculations employed in this study yielded the following increasing orders of energies of interaction for these steroid hormones, respectively, for FA1 and FA6 of HSA: EST > DHT > TES and DHT > TES > EST (Table 1). While these interaction energies do not fully align with the aforementioned experimental reports, further exploration of the theoretical affinities analysis in a global context, encompassing the sum of the interaction energy means of these hormones with FA1 and FA6, yields the following means for HSA:EST, HSA:DHT, and HSA:TES: The values obtained were 48.65, 57.45, and 48.25 kcal mol^–1^, respectively. When FA1 and FA6 HSA are considered together, the order of interaction energy for HSA is as follows: TES > EST > DHT. This order of interaction energy is in accordance with the previously obtained association constants through the utilization of equilibration dialysis and gel filtration,^61,62^ as above-mentioned. This interesting agreement suggests a direct correlation, which has the potential to further help develop a correlation model between energy of interaction and association constants for HSA:Steroid systems. However, it is important to note that the values and impact of computationally obtained interaction energies differ greatly from those of experimental binding affinity parameters.

A comparison between the canonical occupation of the FA1 binding site by ligands such as bilirubin, heme, porphyrin, and fatty acids^48^ and the binding models proposed here for the HSA-FA1 complex with EST, DHT, and TES reveals notable similarities. Published structural data show that (i) heme interacts with FA1 via contacts with ARG114, TYR138, ILE142, HIS146, TYR161, and LYS190; (ii) bilirubin is anchored in FA1 by salt bridges (hydrogen bonds) with ARG117 and ARG186 (TYR138); and (iii) fatty acids are stabilized within FA1 by contacts with ARG117, HIS146, TYR161, and LEU182.^48,63^ In this same site, our models show that EST establishes hydrophobic interactions with ARG117, TYR138, ILE152, TYR161, LEU182, ARG186, and LYS190; DHT makes contacts with ARG117, TYR138, TYR161, and LEU182; and TES interacts with ARG117, TYR138, ILE142, TYR161, and LEU182. Furthermore, residues ARG117, TYR138, and TYR161 showed weaker interaction energy for these hormones.

The similarity between the binding modes we obtained for HSA:hormone complexes and those determined experimentally for canonical ligands indicates potential competition between these steroid hormones and the canonical binders. Furthermore, the similarity observed between these binding modes reinforces the robustness of the final models proposed here. Jayaraj et al. reported that TES displaces oleic acid from at least three known fatty acid sites on HSA, revealing that multiple sites are important for steroid transport in blood serum by HSA. Consequently, we posit that the observed similarities have given rise to the hypothesis that FA1 may function as a potential binding pocket for steroid hormones. However, further investigations employing experimental approaches such as competitive binders and crystallography are necessary to confirm the HSA-FA1:EST/DHT/TES binding.

Regarding FA6, the pattern of residues involved in anchoring steroid hormones in the models proposed here suggests potential competition with fatty acids at this site. Distinct moieties of fatty acids are stabilized within FA6 by ARG209, ASP324, LYS351, GLU354, and SER480.^63^ Our models show that in FA6, EST makes contacts with ARG209, ASP324, LYS351, and GLU354; DHT establishes strong contacts with ARG209, ASP324, LYS351, GLU354, and SER480; and TES engages in hydrophobic contacts with ARG209 and ASP324. Furthermore, the modeling of the HSA-FA6:EST/DHT/TES complexes aligns with the structural data previously obtained for ESA:TES in the homologous binding pocket TBS1.^9^ Several contacts detected in our models are conserved between the HSA-FA1:EST/DHT/TES and ESA-TBS1:TES complexes. For example, the hydrogen bond for EST:GLU354_HSA_ corresponds to TES:GLU353_ESA_ in TBS1. Other conserved hydrophobic contacts were detected: EST/DHT/TES:ALA213_HSA_ (corresponding to TES:ALA212_ESA_), EST/DHT/TES:LEU327_HSA_ (TES:LEU326_ESA_), EST/DHT/TES:LEU331_HSA_ (TES:LEU330_ESA_), DHT/TES:LEU347_HSA_ (TES:LEU346_ESA_), and EST/DHT:ALA350_HSA_ (TES:ALA349_ESA_). These similarities with the ESA-TBS1:TES structure obtained through experimental procedures support the HSA-FA6:Hormone models here obtained employing computational techniques.

HSA plays a critical role in modulating steroid hormone functions in tissues,^11,64^ likely via a capillary-driven delivery system where hormones anchored to HSA are transferred to target tissues.^11^ The binding of EST, DHT, and TES within HSA-FA1 and HSA-FA6, as outlined here through computational modeling, has implications for the bioavailability of sex hormones and the pharmacokinetics of compounds that share these binding pockets, such as fatty acids, bilirubin, heme, and ibuprofen. Consequently, conditions associated with fluctuations in steroid hormone levels (e.g., polycystic ovary syndrome, sepsis, HIV, pregnancy, obesity, metabolic syndrome, and type 2 diabetes mellitus)^65–67^ could directly affect the pharmacokinetics of endogenous or exogenous FA1/FA6 ligands. Conversely, designing compounds that intentionally displace EST, DHT, and TES from HSA’s FA1 and FA6 sites could represent a novel strategy to modulate free hormone levels.

Glycation, a nonenzymatic reaction between a monosaccharide and a protein that results in a covalent bond, affects the secondary and tertiary structures of HSA and has the potential to alter its physiological functions.^48^ Furthermore, molecular dynamics simulations have revealed that glycation of HSA – forming either Schiff base or Amadori adducts – affects the solvent-accessible surface areas of the FA1 and FA6 binding pockets.^26^ Since hyperglycemic patients exhibit increased levels of glycated HSA,^68^ the bioavailability of steroid hormones and their delivery to tissues are likely to be profoundly affected. Notably, LYS137 and LYS351, identified here as critical residues for stabilizing the HSA-FA1:EST/DHT/TES and HSA-FA6:DHT complexes, respectively, are among the glycation sites found in HSA extracted from diabetic patients.^69^ This finding reinforces the significant potential of glycation to disrupt steroid hormone functions in vivo. Beyond glycation, extrinsic factors like temperature and pH are known from spectroscopic and computational studies to modulate HSA′s structure and ligand-binding capacity. It is hypothesized that special concern is necessary to mediate the use of FA1 and FA6’s competitive drugs, especially in conditions associated with fluctuations in HSA levels. Such conditions include hypoalbuminemia, analbuminemia, and hyperalbuminemia, which may affect steroid transport profoundly.^70–72^ Consequently, these factors may also critically influence HSA-mediated steroid hormone transport and bioavailability in disturbance conditions.^73–76^

##### 3.6 Investigation Limitations

The estimation of interaction energy was derived from quantum calculations using the MFCC-DFT approach. Although this method offers valuable per-residue energy decomposition, it does not equate to a thermodynamic binding free energy (Δ*G*). In addition, computational alchemical methods, such as free energy perturbation (FEP) or thermodynamic integration (TI), were not employed. They could provide more data about absolute or relative binding free energies and would offer a more direct, quantitative measure of binding propensity.

MD simulations, while informative for analyzing conformational stability, were conducted as single and short 100 ns trajectories for each complex without independent replicas. This limits the statistical robustness of dynamical observations and the convergence of sampling, potentially affecting the reliability of the stability metrics reported.

The present work relies on computational models and their alignment with previously published experimental data rather than including direct experimental validation. While the observed parallels with earlier reports on association constants and crystallographic binding modes are encouraging and support the plausibility of models here obtained, they do not constitute de novo experimental confirmation of the proposed binding poses or affinities for the specific HSA-FA1/FA6:Hormone complexes.

Furthermore, quantum biochemistry calculations, though insightful, inherit the approximations of the MFCC scheme and the chosen DFT functional/basis set (choice based on previous studies of V. N. Freire research group). These approximations can affect the absolute values of interaction energies, and some calculations on snapshots neglect explicit solvent contributions to interaction energy. Finally, the study focused exclusively on two primary binding sites (FA1 and FA6), guided by initial docking. This targeted approach, while efficient, means that the possibility of other lower-affinity or transient binding regions on HSA was not systematically explored. The transport mechanism of steroid hormones by HSA may involve a more complex equilibrium that involves multiple binding sites.

Despite these limitations, the consistency of the distinct computational results with each other and with established experimental trends lends credibility to the main conclusions. The identified limitations provide a clear direction for future work, emphasizing the necessity of multireplica MD simulations, explicit free energy calculations, and, ultimately, experimental structural biology studies to validate and refine the proposed binding models.

#### 4 Conclusion

This study employed an integrative computational strategy, encompassing molecular docking, molecular dynamics, and quantum biochemistry (MFCC/DFT analysis), to elucidate the noncovalent interactions between HSA and three steroid hormones: EST, DHT, and TES. The docking results indicated that all three hormones exhibited similar binding affinities for HSA, with a preferential interaction at the IB subdomain (FA1) and a secondary hotspot between subdomains IIA and IIB (FA6). The proposed binding modes reveal significant structural similarities between hormone binding at FA1 and the binding of canonical ligands (e.g., heme, bilirubin, fatty acids), suggesting FA1 as a potential competitive binding pocket for steroid hormones. Similarly, the interactions modeled at FA6 show conserved features with experimentally determined structures in homologous proteins, supporting the plausibility of FA6 as an additional steroid-binding site.

MD simulations revealed conformational stability of HSA-DHT complexes at FA6, while EST and TES exhibited substantial fluctuations, suggesting lower compatibility with this site. Quantum biochemistry analysis corroborated these findings, showing that EST forms slightly more stable interactions with FA1, whereas DHT exhibits stronger and more sustained binding to FA6. The study emphasizes that binding stability is not solely determined by affinity but also by structural compatibility with a specific site, as evidenced by the enhanced MD stability of DHT at FA6 compared to EST/TES.

The total energy of interaction order derived from the combined analysis of HSA:Hormone, DHT > EST > TES, aligns with the affinity trends reported in previous experimental studies, thereby validating the robustness of the computational approach employed here.

In summary, these results collectively highlight the nuanced binding behavior of sex hormones with HSA, where site-specific interactions and structural compatibility govern complex stability. The identification of FA6 as a favorable site for DHT, particularly, offers novel insights into its transport dynamics and the potential for drug-hormone competition at this site. The binding of these hormones to FA1 and FA6 has direct implications for their serum transport, bioavailability, and pharmacokinetics. It suggests potential competition with endogenous compounds (fatty acids, bilirubin) and drugs (e.g., ibuprofen) that share these sites. This competition is crucial for understanding hormone dynamics in various physiological and pathological states. Identification of FA6 as a viable binding pocket expands the map of potential competition. Screening new drug candidates for affinity to FA6, in addition to traditional sites, could predict unexpected interactions with endogenous steroids like DHT, potentially explaining off-target hormonal side effects. Theoretical results obtained in this study suggest that FA6 can be used to extend the circulating half-life of a steroid drug. This could be achieved, for example, by introducing functional groups that stabilize interactions at this site.

Furthermore, the substantial impact of aromatic and hydrophobic interactions, particularly within the FA1 complexes, corroborates extant experimental findings and emphasizes the necessity for site-specific consideration in pharmacokinetic modeling and drug design. The integration of classical and quantum-level computational approaches was ultimately found to be effective in capturing the intricate details of HSA:Hormone interactions. This finding serves to support the reliability of the utilized modeling framework and provides a basis for the theoretical investigation of other bioactive compounds in future studies.

## Supplementary Material



## Notes

Additional figures illustrating hormone binding at the interaction site; hydrogen-bond interactions; ligand distances to key amino acid residues throughout the simulation; RMSF analyses; tables detailing interaction energy of each protein residue with the ligands (PDF)
