# Supplementary material for: Binding of Steroid Hormones to the FA1 and FA6 Sites of Human Serum Albumin through Computational Biology and Quantum Biochemistry
Source: ACS Omega. 2026 Mar 24;11(13):20380–99. doi: 10.1021/acsomega.5c11421 (PMC13063194; doi:10.1021/acsomega.5c11421)
Supplement: Supplementary file 1 [file ao5c11421_si_001.pdf]

The Binding of Steroid Hormones to the FA1 and FA6 Sites of Human Serum Albumin Through Computational Biology and Quantum Biochemistry

Victor L.B. França[a,b], André Hadad[a], Jackson L. Amaral[c*], Francisco R. X. Vieira[a], Hernandes F. Carvalho[d], Valder N. Freire[a]

[a] *Department of Physics, Federal University of Ceará, 60440-900 Fortaleza, Ceará, Brazil*

[b] *Department of Physiology and Pharmacology, Faculty of Medicine, Federal University of Ceará, 60430-160 Fortaleza, Ceará, Brazil*

[c] *Department of Biochemistry and Pharmacology, Federal University of Piaui, CEP 64049-55 Teresina, Piaui, Brazil.*

[d] *Department of Structural and Functional Biology, Institute of Biology, State University of Campinas, 13083-864 Campinas, São Paulo, Brazil.*

***Corresponding Author**

Federal University of Piaui. Department of Biochemistry and Pharmacology,
Av. Petronio Portella, Ininga, Teresina – PI, 64049-550, Brazil.
E-mail: jacksoncesarc@gmail.com
ORCID: 0009-0005-8483-732X (J. L. Amaral)

Supplementary Figure 1 – Evolution of steroid hormone occupancy in FA1 and FA6 of HSA during molecular dynamics simulations, analyzed in 20 ns intervals. The occupancy of FA1 by (A) estradiol (EST), (B) dihydrotestosterone (DHT), and (C) testosterone (TES) is shown. Progressive changes in the positioning of (D) EST, (E) DHT, and (F) TES within FA6 are also illustrated.

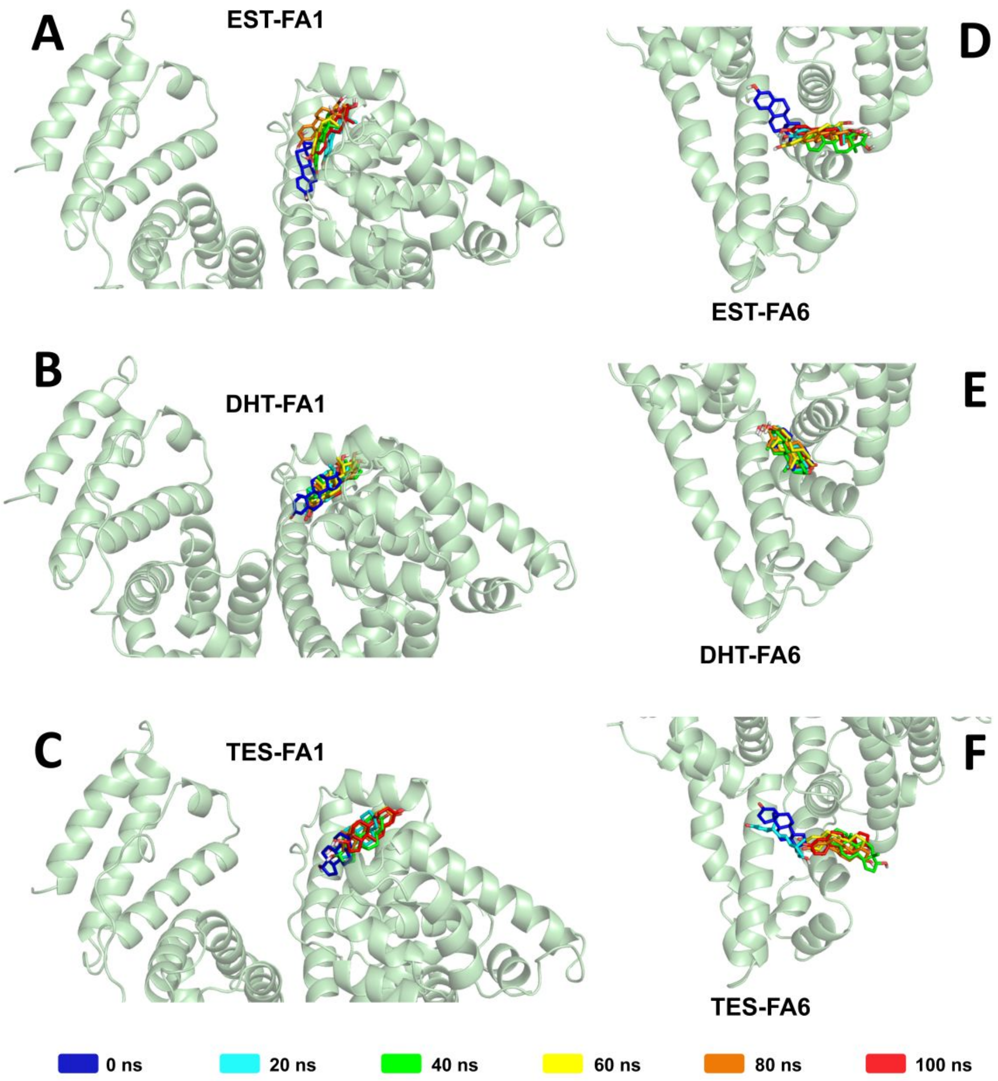

Supplementary Figure 2 – Time evolution of distances between steroid hormones and key FA1 residues. Distance between the center of mass of each steroid hormone and specific FA1 amino acid residues is plotted as a function of simulation time.

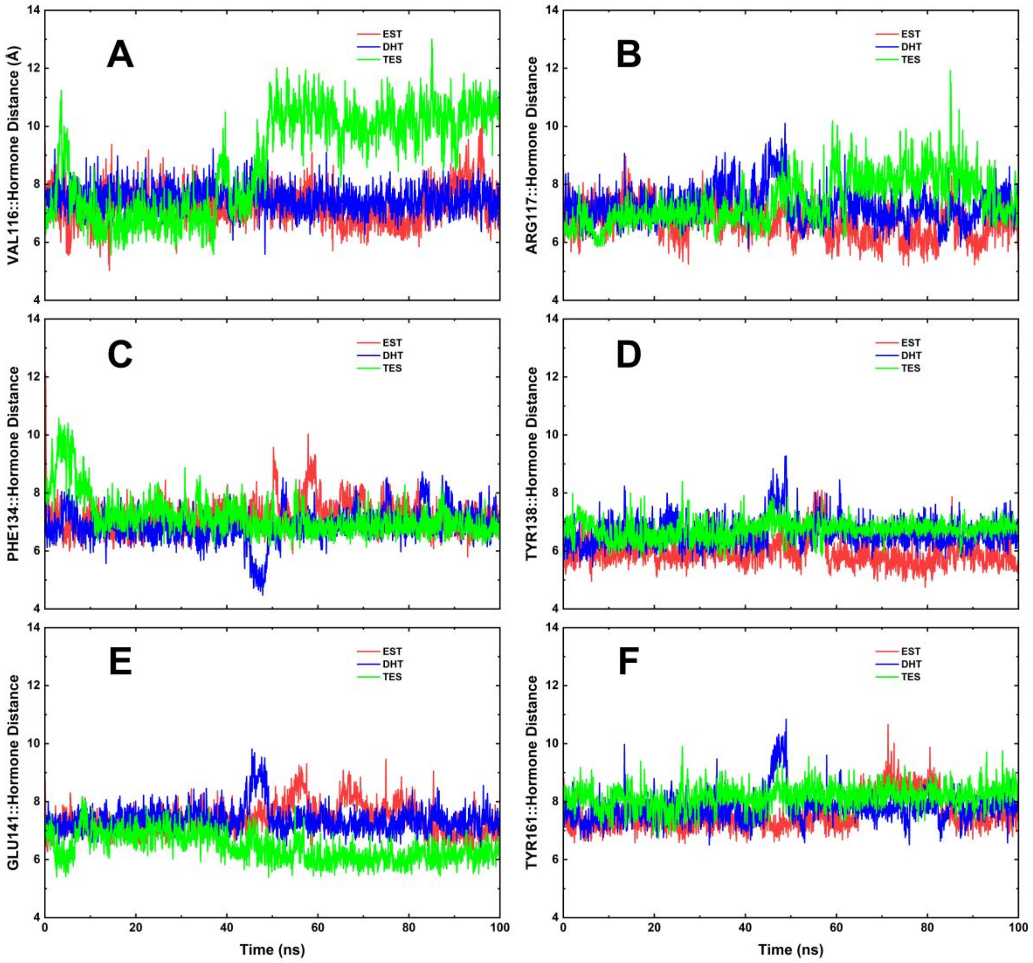

Supplementary Figure 3 – Time evolution of distances between steroid hormones and key FA6 residues. Distance between the center of mass of each steroid hormone and specific FA6 amino acid residues is plotted as a function of simulation time.

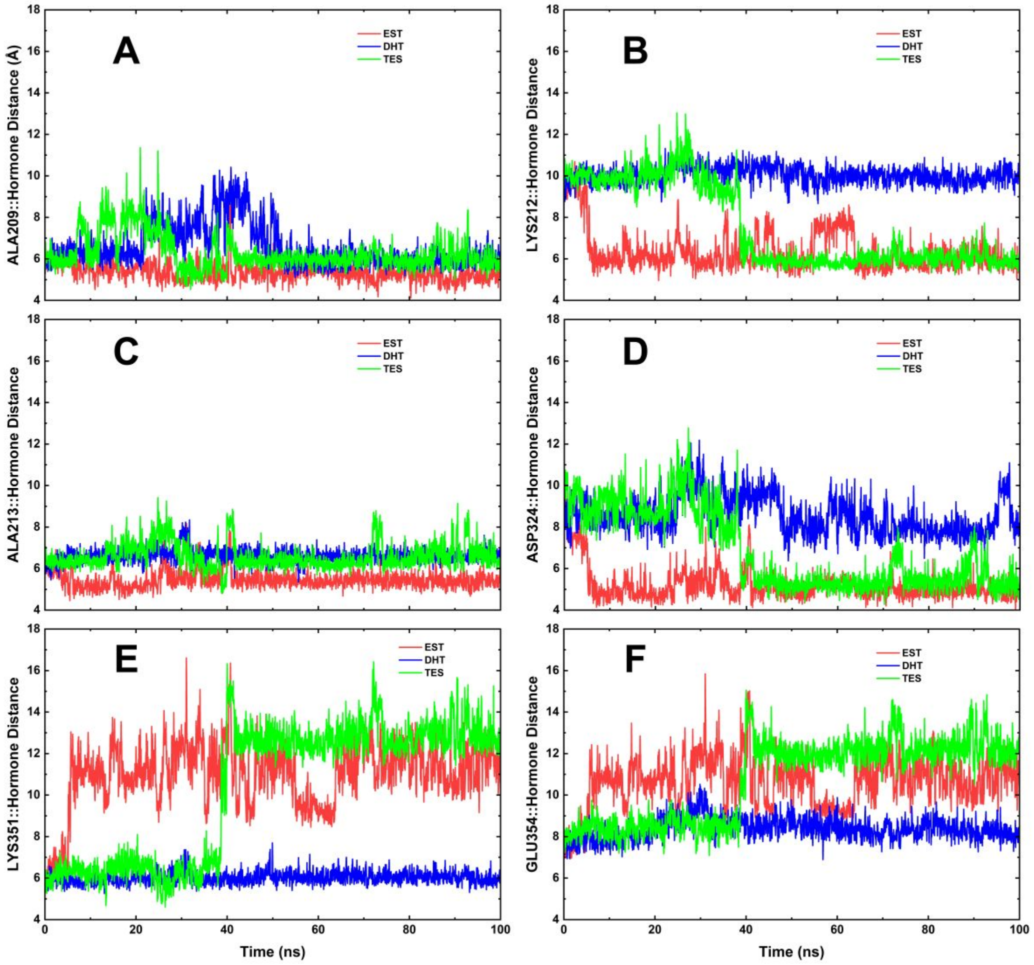

Supplementary Figure 4 – Characterization of hydrogen bonds between HSA and the steroid hormones (EST, DHT, TES) during molecular dynamics simulations. Time evolution of the number of hydrogen bonds with (A) HSA-FA1 and (B) HSA-FA6. Occupancy of specific hydrogen bond contacts for the (C) HSA-FA1::Hormone and (D) HSA-FA6::Hormone interfaces are illustrated on the charts.

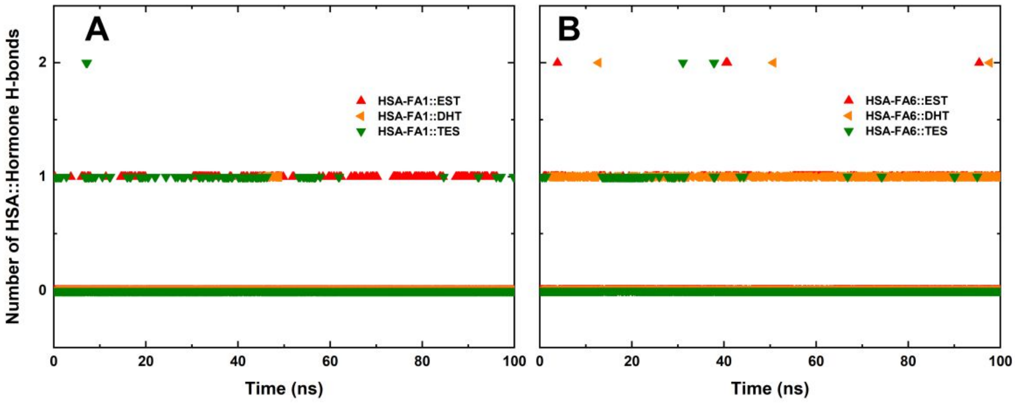

C

Binding Pocket	Interaction (Donor::Aceptor)	Occupancy (%)
	EST::VAL116	0.05
	EST::LEU182	0.30
	LYS137::EST	8.54
	ARG117::EST	2.55
FA1	DHT::ASN130	0.85
	TYR161::DHT	0.30
	ARG117::TES	7.94
	LYS137::TES	0.15
	TES::LEU115	0.10

D

Binding Pocket	Interaction (Donor::Aceptor)	Occupancy (%)
	EST::ASP324	1.85
	EST::LEU347	0.10
	EST::GLU354	15.38
	EST::SER232	3.00
	EST::GLU208	0.05
	EST::THR236	0.25
	EST::ASP324	0.05
FA6	LEU481::DHT	24.33
	VAL482::DHT	2.85
	ARG209::DHT	0.10
	LEU481::TES	0.85
	TES::ASP324	6.54
	ASN483::TES	0.15
	VAL482::TES	0.30
	TES::SER232	0.45

Supplementary Figure 5 – RMSF values calculated for HSA::Hormone complexes. Fluctuation data of the HSA binding to EST, DHT, and TES through (A) FA1 and (B) FA6 are represented. HSA's subdomains are indicated in grey letters.

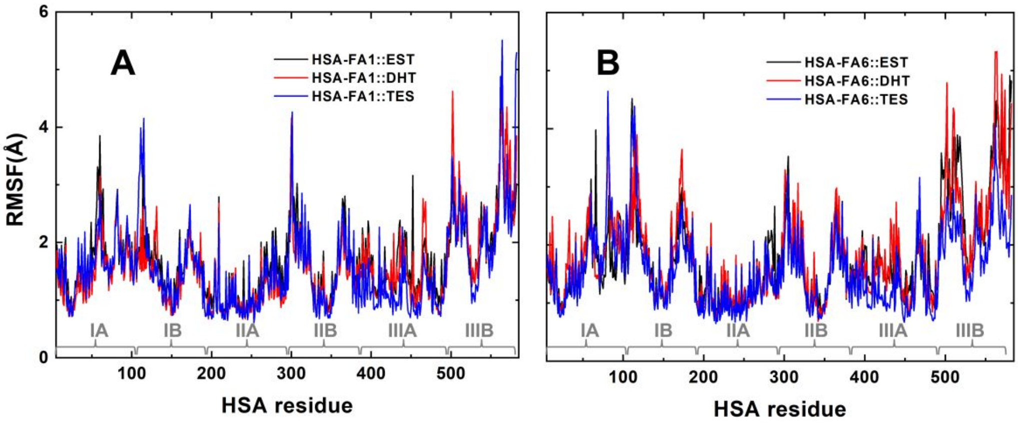

Supplementary Table 1 – Description of the quantum mechanics calculations of the conformation 0 of HSA-FA1::EST.

HSA Residue	Distance (Å)	Interaction Energy (kcal/mol)	Residue Charge	EST Charge	HSA Atom	EST Atom
LEU182	2.05	-2.74	0	0	HD12	H5
PRO118	2.08	-2.83	0	0	HD3	H18
LYS137	2.08	-7.71	1	0	HZ2	O17
LEU115	2.19	-6.11	0	0	HD13	H2
ARG117	2.28	-4.01	1	0	HG2	H7
TYR138	2.30	-4.98	0	0	HE1	H4
PHE134	2.30	-4.17	0	0	HE1	H24
TYR161	2.62	-6.74	0	0	HE1	C5
PHE165	2.77	-2.28	0	0	HE2	H6
GLU141	2.83	-1.79	-1	0	HG3	H15
ILE142	2.86	-1.06	0	0	HD11	H4
LEU185	2.95	-1.11	0	0	HB3	H3
MET123	3.00	-3.42	0	0	SD	H8
VAL116	3.33	-1.05	0	0	O	H9
ARG186	3.40	-0.91	1	0	HB3	H3
ALA126	3.75	-0.69	0	0	HB2	H19
LEU179	5.03	-0.26	0	0	HD22	H7
ASP183	5.37	0.23	-1	0	N	O3
THR133	5.44	-0.23	0	0	O	H23
LEU178	5.61	-0.23	0	0	HD22	H7
LEU135	5.65	-0.09	0	0	N	H23
LYS181	5.73	-0.27	1	0	O	O3
VAL122	5.90	-0.19	0	0	HG11	H20
PHE127	5.94	-0.22	0	0	HB3	H22
TYR140	6.17	-0.24	0	0	HD2	H15
ARG145	6.18	-0.06	1	0	HH11	H14
PHE157	6.22	-0.07	0	0	HB2	H3
LEU139	6.39	-0.23	0	0	N	H12
ARG114	6.42	-0.20	1	0	O	H11
ASP187	6.61	-0.08	-1	0	H	H3
GLU184	6.61	0.07	-1	0	C	H3
LYS136	6.66	-0.11	1	0	C	H23
ALA164	6.68	-0.06	0	0	HB1	H5
GLY189	6.86	-0.02	0	0	HA3	H3
PHE36	6.90	-0.20	0	0	HD2	H21
CYS124	7.09	-0.09	0	0	N	H17
GLU119	7.12	-0.11	-1	0	O	H8
VAL120	7.21	-0.14	0	0	HG23	H7
ALA158	7.28	-0.05	0	0	HA	H4
LEU154	7.48	-0.05	0	0	HD12	H4
ARG144	7.52	0.03	1	0	HH12	H14

LYS190	7.55	0.04	1	0	HG3	H3
GLU188	7.87	0.08	-1	0	H	H3
GLU131	7.93	0.12	-1	0	O	H23
PHE149	7.94	-0.02	0	0	HE1	H4
ASP129	7.98	-0.02	-1	0	HB2	H24
HIS146	8.05	0.00	0	0	HD2	H4
THR125	8.11	-0.04	0	0	C	H19
ASN130	8.18	-0.12	0	0	O	H24
PRO113	8.29	-0.03	0	0	O	H14
GLU132	8.29	0.11	-1	0	O	H23
ARG160	8.37	0.00	1	0	HB3	H3
ALA143	8.53	-0.02	0	0	H	H12
PRO180	8.54	-0.02	0	0	O	O3
LYS162	8.64	-0.01	1	0	HA	H6
HIS128	8.84	-0.07	0	0	H	H17
ASP121	9.33	0.02	-1	0	O	H17
CYS169	9.37	-0.02	0	0	SG	H6
GLU37	9.55	0.10	-1	0	HG3	H21
CYS168	9.67	-0.06	0	0	HB2	H5
LEU24	9.70	-0.01	0	0	HD22	H15
LEU112	9.79	-0.02	0	0	HD11	H14

Supplementary Table 2 – Description of the quantum mechanics calculations of the conformation 1 of HSA-FA1::EST.

HSA Residue	Distance (Å)	Interaction Energy (kcal/mol)	Residue Charge	EST Charge	HSA Atom	EST Atom
LEU115	2.03	-5.63	0	0	HB2	H2
ARG117	2.14	-10.00	1	0	HH22	O3
TYR161	2.19	-3.32	0	0	HE1	H6
VAL116	2.30	-3.32	0	0	O	H12
TYR138	2.36	-7.52	0	0	HD1	H10
LEU182	2.39	-4.25	0	0	HD22	H7
ARG186	2.45	-0.85	1	0	HB3	H3
PHE134	2.47	-3.75	0	0	HE2	H20
PRO118	2.49	-4.17	0	0	HD2	H14
LYS137	2.52	-2.60	1	0	HB2	H24
GLU141	2.78	-1.53	-1	0	HG3	H23
PHE165	2.89	-2.21	0	0	HZ	H7
MET123	2.97	-3.15	0	0	SD	H9
ALA126	4.42	-0.26	0	0	HB2	H20
LEU185	4.48	-0.33	0	0	HB3	H5
LEU178	4.62	-0.33	0	0	HD12	H7
ASP183	4.68	0.03	-1	0	HA	O3
THR133	5.47	-0.34	0	0	O	H19
LEU135	5.50	-0.02	0	0	HA	H17
ILE142	5.99	-0.29	0	0	HD11	H3
LEU139	6.03	-0.23	0	0	H	H22
ARG114	6.10	-0.28	1	0	C	H2
PHE127	6.13	-0.13	0	0	HB3	H18
ASP187	6.16	-0.07	-1	0	H	H3
TYR140	6.23	-0.24	0	0	HD2	H23
VAL122	6.32	-0.15	0	0	HG12	H20
LYS136	6.44	-0.04	1	0	C	H24
LEU179	6.53	-0.14	0	0	O	O3
PHE157	6.55	-0.09	0	0	HZ	H3
ARG145	6.71	-0.03	1	0	HH11	H23
GLU184	6.81	0.04	-1	0	N	H3
LYS181	6.81	-0.11	1	0	O	H5
ALA164	7.27	-0.02	0	0	HB2	H7
VAL120	7.29	-0.16	0	0	HA	H9
CYS124	7.33	-0.07	0	0	N	H18
GLY189	7.37	-0.02	0	0	H	H3
LYS190	7.51	0.03	1	0	HG3	H3
ARG144	7.62	0.03	1	0	HH12	H23
GLU119	7.67	-0.14	-1	0	N	H16
PHE36	7.73	-0.13	0	0	HE2	H19
GLU131	7.87	0.04	-1	0	O	H17

ASN130	7.88	-0.12	0	0	O	H19
GLU188	7.97	0.09	-1	0	H	H3
GLU132	8.20	0.06	-1	0	O	H19
PRO113	8.31	-0.03	0	0	O	H13
THR125	8.38	-0.01	0	0	C	H20
PRO180	8.45	-0.01	0	0	O	O3
CYS168	8.85	-0.06	0	0	HB2	H7
HIS146	8.95	0.00	0	0	HE2	H3
LYS162	8.98	0.05	1	0	N	H6
ASP129	9.04	-0.05	-1	0	HB2	H20
ALA158	9.18	-0.01	0	0	HA	H6
CYS169	9.25	-0.02	0	0	SG	H9
ALA143	9.36	-0.01	0	0	H	H22
ARG160	9.41	0.09	1	0	O	H7
HIS128	9.43	-0.06	0	0	H	H20
ASP121	9.98	0.01	-1	0	C	H20

Supplementary Table 3 – Description of the quantum mechanics calculations of the conformation 2 of HSA-FA1::EST.

HSA Residue	Distance (Å)	Interaction Energy (kcal/mol)	Residue Charge	EST Charge	HSA Atom	EST Atom
GLU141	1.88	-3.35	-1	0	HG3	H12
LYS137	2.01	-9.53	1	0	HZ3	O17
TYR161	2.25	-5.47	0	0	HH	H6
TYR138	2.39	-6.40	0	0	HE1	H4
LEU182	2.39	-2.68	0	0	HD21	H5
ILE142	2.47	-1.14	0	0	HD12	H4
PHE134	2.57	-5.64	0	0	HE1	H17
LEU115	2.63	-4.84	0	0	HD12	H2
PRO118	2.70	-2.65	0	0	HG2	H18
VAL116	3.02	-1.15	0	0	O	H9
MET123	3.30	-2.81	0	0	HG2	H8
PHE165	3.52	-1.02	0	0	HZ	H6
ARG117	3.59	-1.28	1	0	HA	H9
ARG186	3.69	-0.66	1	0	HA	H3
ALA126	3.75	-0.51	0	0	HB3	H19
LEU185	4.59	-0.66	0	0	HB3	O3
TYR140	4.89	-0.43	0	0	HD2	H15
ARG145	5.15	-0.24	1	0	HH11	H12
LEU135	5.42	-0.04	0	0	N	H23
VAL122	5.49	-0.20	0	0	HG13	H20
THR133	5.74	-0.21	0	0	O	H24
LEU139	5.90	-0.36	0	0	H	H23
PHE127	6.01	-0.20	0	0	HB3	H17
LYS136	6.05	-0.12	1	0	C	H23
LEU178	6.08	-0.13	0	0	HD12	H6
GLY189	6.26	-0.03	0	0	HA3	H3
ASP183	6.30	0.08	-1	0	N	O3
PHE157	6.44	-0.05	0	0	HD2	H3
ARG144	6.58	-0.05	1	0	HD3	H12
ASP187	6.67	-0.04	-1	0	H	H3
LEU179	6.69	-0.07	0	0	HD22	H7
LYS181	6.83	-0.02	1	0	O	O3
PHE36	7.03	-0.20	0	0	CD2	O17
HIS146	7.15	-0.02	0	0	HD2	H4
CYS124	7.17	-0.08	0	0	N	H17
LYS190	7.18	0.03	1	0	HG3	H3
ASN130	7.31	-0.13	0	0	O	H24
LEU154	7.37	-0.06	0	0	HD12	H4
ARG114	7.45	-0.11	1	0	O	H11
GLU184	7.56	0.05	-1	0	C	H3
ALA143	7.59	-0.04	0	0	H	H2

GLU119	7.63	-0.08	-1	0	O	H8
GLU131	7.65	0.10	-1	0	O	H24
VAL120	7.85	-0.11	0	0	HA	H8
PHE149	7.93	-0.02	0	0	HE1	H3
THR125	7.95	-0.03	0	0	C	H19
ASP129	7.98	-0.04	-1	0	HB2	H19
ALA164	8.05	-0.04	0	0	HB2	H5
GLU188	8.24	0.09	-1	0	H	H3
ALA158	8.28	-0.02	0	0	HA	H4
GLU132	8.29	0.10	-1	0	O	H24
PRO113	8.65	-0.01	0	0	HB3	H14
HIS128	8.71	-0.06	0	0	H	H19
LEU24	8.84	-0.02	0	0	HD21	H15
LEU112	8.85	-0.02	0	0	HD12	H12
PRO180	9.13	-0.01	0	0	O	O3
LYS162	9.44	0.02	1	0	HA	H22
ASP121	9.53	0.01	-1	0	O	H17
CYS169	9.64	-0.02	0	0	SG	H6
ARG160	9.80	0.06	1	0	C	O3

Supplementary Table 4 – Description of the quantum mechanics calculations of the conformation 3 of HSA-FA1::EST.

HSA Residue	Distance (Å)	Interaction Energy (kcal/mol)	Residue Charge	EST Charge	HSA Atom	EST Atom
ARG117	2.11	-4.48	1	0	HA	H9
TYR161	2.30	-3.99	0	0	HE1	H6
LEU115	2.35	-4.73	0	0	HD11	H2
PRO118	2.37	-4.93	0	0	HD3	H8
GLU141	2.40	-2.91	-1	0	HG3	H15
PHE134	2.43	-3.70	0	0	HE1	H17
ARG186	2.47	-2.28	1	0	HB3	O3
LEU182	2.56	-3.85	0	0	O	H3
LYS137	2.65	-3.46	1	0	HD3	H21
TYR138	2.67	-6.45	0	0	HD1	H13
MET123	2.75	-2.98	0	0	HG2	H18
VAL116	2.75	-1.70	0	0	O	H11
ILE142	3.11	-1.39	0	0	HD11	H4
PHE165	3.33	-0.84	0	0	HZ	H6
LEU185	3.77	-0.99	0	0	HB3	H3
ASP183	4.45	-0.68	-1	0	HA	H3
ALA126	4.60	-0.26	0	0	HB3	H19
VAL122	5.17	-0.22	0	0	HG13	H20
TYR140	5.69	-0.39	0	0	HD2	H22
LEU139	5.72	-0.32	0	0	H	H22
LEU179	5.84	-0.16	0	0	HD21	H7
LYS181	5.85	0.08	1	0	O	H3
ASP187	5.87	-0.09	-1	0	H	H3
THR133	5.95	-0.15	0	0	O	H23
ARG145	5.95	-0.13	1	0	HH11	H12
GLU184	6.02	-0.01	-1	0	N	H3
LYS136	6.02	-0.30	1	0	C	H22
ARG114	6.07	-0.02	1	0	O	H14
LEU135	6.11	-0.14	0	0	N	H23
LEU178	6.30	-0.14	0	0	HD23	H7
GLY189	6.43	-0.06	0	0	H	O3
HIS146	6.69	-0.04	0	0	HE2	H4
PHE127	6.69	-0.09	0	0	HB3	H17
GLU119	6.79	-0.17	-1	0	O	H18
ARG144	6.90	-0.02	1	0	HH12	H15
VAL120	7.21	-0.14	0	0	HA	H8
CYS124	7.30	-0.06	0	0	N	H18
GLU188	7.30	0.05	-1	0	H	H3
PHE36	7.31	-0.16	0	0	HB3	H21
LYS190	7.42	-0.03	1	0	HG3	H4
ALA164	7.61	-0.02	0	0	HB1	H6

PRO113	7.82	-0.02	0	0	O	H12
PRO180	7.85	-0.03	0	0	O	H3
ALA143	7.94	-0.03	0	0	H	H13
PHE157	7.99	-0.05	0	0	HB2	H3
PHE149	8.24	-0.03	0	0	HE1	H4
GLU132	8.46	0.10	-1	0	O	H23
ALA158	8.62	-0.03	0	0	HA	H24
ASN130	8.69	-0.13	0	0	O	H19
GLU131	8.72	0.11	-1	0	O	H23
THR125	8.76	-0.02	0	0	H	H18
LYS162	8.79	0.03	1	0	N	H6
LEU154	8.80	-0.05	0	0	HD13	H4
LEU24	8.89	-0.01	0	0	HD22	H22
ASP129	8.97	-0.01	-1	0	HB2	H19
ASP121	9.00	-0.02	-1	0	C	H18
ARG160	9.47	0.08	1	0	O	H5
LEU112	9.73	-0.02	0	0	HB2	H12

Supplementary Table 5 – Description of the quantum mechanics calculations of the conformation 4 of HSA-FA1::EST.

HSA Residue	Distance (Å)	Interaction Energy (kcal/mol)	Residue Charge	EST Charge	HSA Atom	EST Atom
LEU182	1.91	-1.85	0	0	HD11	H5
PRO118	2.03	-3.60	0	0	HD3	H18
ARG117	2.14	-7.78	1	0	HA	H9
TYR138	2.20	-7.28	0	0	HD1	H2
LYS137	2.35	-6.24	1	0	HZ1	H21
TYR161	2.50	-6.92	0	0	HH	C5
GLU141	2.52	-2.96	-1	0	HG3	H14
PHE134	2.76	-5.25	0	0	HE2	H17
MET123	2.87	-3.13	0	0	SD	H8
LEU115	3.01	-4.00	0	0	HD21	H2
PHE165	3.11	-1.30	0	0	HZ	H6
VAL116	3.33	-1.40	0	0	O	H9
ILE142	3.39	-1.06	0	0	HD12	H4
ARG186	3.42	-0.75	1	0	HB3	H3
LEU185	4.49	-0.34	0	0	HD22	H3
ALA126	4.56	-0.29	0	0	HB1	H19
PHE157	4.73	-0.27	0	0	HZ	H3
LEU135	5.15	-0.15	0	0	HA	H22
THR133	5.36	-0.34	0	0	O	H22
LEU179	5.36	-0.15	0	0	HD22	H5
LEU178	5.40	-0.21	0	0	HD22	H6
VAL122	5.48	-0.33	0	0	HG12	H20
TYR140	5.73	-0.39	0	0	HD2	H15
LEU139	5.89	-0.35	0	0	N	H15
ARG145	5.94	-0.06	1	0	HH11	H14
LYS136	5.96	-0.22	1	0	C	H22
ASP183	6.00	0.10	-1	0	N	O3
HIS146	6.47	-0.03	0	0	HE2	H4
PHE127	6.51	-0.15	0	0	HB3	H17
GLU119	6.54	-0.16	-1	0	O	H18
VAL120	6.76	-0.17	0	0	HA	H8
CYS124	6.97	-0.08	0	0	N	H17
ARG144	6.99	0.02	1	0	HH12	H14
LYS181	7.06	-0.13	1	0	O	H3
ASP187	7.30	-0.02	-1	0	H	H3
GLY189	7.43	-0.01	0	0	HA3	H3
PHE36	7.55	-0.16	0	0	CD2	H21
ASN130	7.59	-0.15	0	0	O	H23
GLU131	7.75	0.08	-1	0	O	H22
GLU184	7.89	0.08	-1	0	N	H3
GLU132	8.14	0.08	-1	0	O	H22

LEU154	8.23	-0.05	0	0	HD12	H4
ALA143	8.27	-0.03	0	0	H	H12
PHE149	8.29	-0.02	0	0	HE1	H4
ALA158	8.30	-0.03	0	0	HA	H4
THR125	8.41	-0.02	0	0	H	H17
LYS190	8.43	0.05	1	0	H	H3
ARG114	8.44	-0.05	1	0	HH12	H9
ALA164	8.46	-0.04	0	0	HB3	O3
LEU24	8.66	-0.02	0	0	HD22	H15
ASP121	8.74	0.00	-1	0	C	H18
LYS162	8.88	0.02	1	0	HA	H24
GLU188	8.93	0.10	-1	0	H	H3
ARG160	8.94	0.04	1	0	HB3	H3
PRO180	8.95	0.00	0	0	N	H5
HIS128	9.38	-0.06	0	0	H	H17
ASP129	9.40	-0.04	-1	0	HB3	H19
CYS169	9.50	-0.02	0	0	SG	H6

Supplementary Table 6 – Description of the quantum mechanics calculations of the final conformation of HSA-FA1::EST.

HSA Residue	Distance (Å)	Interaction Energy (kcal/mol)	Residue Charge	EST Charge	HSA Atom	EST Atom
ARG117	2.08	-3.15	1	0	HG2	H7
TYR138	2.12	-8.69	0	0	HA	H13
VAL116	2.40	-2.01	0	0	O	H9
GLU141	2.43	-4.36	-1	0	HG1	H12
PHE134	2.44	-4.65	0	0	HE2	H19
LYS137	2.46	-4.38	1	0	HB1	H22
TYR161	2.55	-3.49	0	0	OH	H10
LEU115	2.73	-5.38	0	0	HD22	H11
LEU182	2.82	-2.00	0	0	HD23	H5
MET123	3.00	-3.47	0	0	HE1	H8
ILE142	3.05	-1.83	0	0	HG12	H4
PRO118	3.11	-1.70	0	0	HD1	H18
ARG186	3.35	-0.92	1	0	HB1	H3
ALA126	4.15	-0.38	0	0	HB2	H19
TYR140	4.79	-0.50	0	0	HD2	H15
LEU135	4.83	0.06	0	0	HA	H22
PHE165	4.99	-0.47	0	0	HZ	H6
THR133	5.09	-0.30	0	0	O	H22
LEU139	5.26	-0.51	0	0	N	H13
ASP183	5.50	-0.11	-1	0	HA	H3
ARG145	5.56	-0.35	1	0	HH11	H12
LYS136	5.66	-0.39	1	0	C	H22
LEU185	5.67	-0.32	0	0	HB1	O3
PHE127	6.03	-0.16	0	0	HB1	H17
VAL122	6.19	-0.13	0	0	HG11	H20
ARG144	6.93	-0.17	1	0	HH12	H12
LYS190	6.98	-0.12	1	0	HD2	H3
PHE36	7.01	-0.15	0	0	HE1	H15
LEU154	7.04	-0.03	0	0	HD12	H4
HIS146	7.07	-0.05	0	0	HD2	H4
LEU178	7.08	-0.07	0	0	HB2	H6
ASP187	7.14	-0.02	-1	0	HN	O3
LEU179	7.23	-0.07	0	0	HD23	H6
ALA143	7.40	-0.06	0	0	HN	H2
GLU131	7.48	0.03	-1	0	O	H22
GLY189	7.51	-0.03	0	0	HA1	H4
PHE149	7.54	-0.03	0	0	HE1	H4
ARG114	7.60	-0.08	1	0	C	H11
ASN130	7.72	-0.04	0	0	O	H23
GLU119	7.79	-0.12	-1	0	O	H18
LYS181	7.81	-0.06	1	0	C	H5

CYS124	7.81	-0.04	0	0	N	H18
PHE157	7.83	-0.06	0	0	HB2	H4
GLU184	7.85	0.02	-1	0	HN	H3
GLU132	7.86	0.04	-1	0	O	H22
ALA158	8.10	-0.02	0	0	HA	H24
THR125	8.20	-0.03	0	0	C	H19
VAL120	8.24	-0.04	0	0	HA	H8
ASP129	8.26	0.00	-1	0	HB2	H19
GLU188	8.77	0.07	-1	0	HN	O3
LEU112	8.81	-0.02	0	0	HD12	H12
LEU24	8.84	-0.02	0	0	HD21	H22
LYS162	8.88	-0.08	1	0	HA	H24
PRO113	8.89	-0.02	0	0	O	H11
HIS128	9.17	-0.02	0	0	HN	H19
ALA164	9.36	-0.03	0	0	HB1	H6
PRO180	9.40	-0.01	0	0	O	H5
ASP121	9.82	-0.01	-1	0	C	H18

Supplementary Table 7 – Description of the quantum mechanics calculations of the conformation 0 of HSA-FA6::EST.

HSA Residue	Distance (Å)	Interaction Energy (kcal/mol)	Residue Charge	EST Charge	HSA Atom	EST Atom
GLU354	1.96	-6.97	-1	0	OE2	H3
LEU327	2.19	-3.82	0	0	HB3	H7
LYS351	2.21	-1.55	1	0	HZ1	O3
ALA213	2.23	-4.45	0	0	HB3	H2
ARG209	2.29	-9.90	1	0	HG2	H13
ASP324	2.36	-5.38	-1	0	HA	H6
LYS212	2.42	-4.58	1	0	HB2	H14
PHE228	2.58	-2.19	0	0	HZ	H20
GLY328	2.69	-2.62	0	0	HA2	H8
VAL216	2.76	-2.92	0	0	HG23	H1
LEU331	3.10	-1.90	0	0	HD22	H9
ALA210	3.54	-0.98	0	0	HA	H2
LYS323	3.76	-1.76	1	0	HG2	H6
VAL235	3.96	-0.53	0	0	HG12	H1
VAL325	4.20	-0.55	0	0	HA	H17
SER232	4.34	-0.39	0	0	OG	H20
GLU208	4.42	-0.53	-1	0	O	H15
ALA350	5.04	-0.28	0	0	HB1	H7
MET329	5.39	-0.14	0	0	H	H8
TRP214	5.47	-0.27	0	0	H	H11
LEU347	5.55	-0.47	0	0	HD22	C2
PHE211	5.58	-0.33	0	0	C	H14
ALA215	5.66	-0.18	0	0	HB2	H1
PHE326	5.89	-0.27	0	0	C	H8
THR236	5.92	-0.13	0	0	OG1	H21
PHE330	6.06	-0.15	0	0	HB2	H7
TYR332	6.10	-0.21	0	0	HB3	H18
ALA217	6.33	-0.13	0	0	H	H16
THR355	6.50	-0.12	0	0	HG22	H3
TYR319	6.70	-0.08	0	0	HE1	H5
VAL231	6.83	-0.10	0	0	HG13	H1
GLY207	7.08	-0.07	0	0	O	H12
PHE206	7.18	-0.03	0	0	O	H2
ALA322	7.25	-0.08	0	0	O	H6
ALA229	7.34	-0.06	0	0	HA	H20
TYR353	7.45	-0.07	0	0	HB2	H5
THR352	7.55	-0.03	0	0	N	H3
LYS233	7.63	0.00	1	0	N	H21
THR239	7.76	-0.01	0	0	HG21	H15
LEU481	7.96	-0.03	0	0	HD22	H4
VAL482	8.00	-0.04	0	0	HG21	H4

SER480	8.07	-0.07	0	0	HG	H4
SER220	8.37	-0.02	0	0	HG	H18
ARG348	8.53	-0.04	1	0	HA	H3
LEU346	8.64	-0.05	0	0	O	H5
LEU357	8.65	-0.04	0	0	HD13	H5
LEU349	8.70	-0.04	0	0	C	H3
LEU203	8.71	-0.02	0	0	HD13	H15
GLU333	8.95	-0.06	-1	0	H	H18
LEU219	8.96	-0.03	0	0	HD11	H1
LEU234	9.10	-0.01	0	0	C	H21
SER202	9.13	-0.01	0	0	OG	H2
ARG218	9.19	-0.01	1	0	H	H16
ASP237	9.30	0.06	-1	0	H	H21
PHE309	9.43	-0.10	0	0	HE2	H7
ALA335	9.49	-0.01	0	0	HB3	H9
LEU238	9.53	-0.03	0	0	HD23	H1
THR356	9.54	0.00	0	0	H	H3
GLU227	9.61	0.00	-1	0	O	H20
GLU230	9.74	0.03	-1	0	N	H20
GLU358	9.95	-0.01	-1	0	HB3	H3

Supplementary Table 8 – Description of the quantum mechanics calculations of the conformation 1 of HSA-FA6::EST.

HSA Residue	Distance (Å)	Interaction Energy (kcal/mol)	Residue Charge	EST Charge	HSA Atom	EST Atom
ALA213	2.13	-5.85	0	0	HA	H9
ASP324	2.13	-5.02	-1	0	HB2	H13
ARG209	2.20	-6.36	1	0	HG3	H12
GLY328	2.24	-1.73	0	0	HA3	H8
LEU331	2.24	-3.10	0	0	HD21	H7
VAL216	2.25	-4.10	0	0	HG11	H18
LYS212	2.30	-5.40	1	0	HB3	H14
VAL325	2.43	-3.62	0	0	HA	H23
ALA350	2.85	-1.14	0	0	HB2	H5
PHE228	2.90	-2.07	0	0	HE1	H19
VAL235	3.06	-1.76	0	0	HG12	H1
LEU327	3.23	-2.75	0	0	C	H6
SER232	3.48	-1.00	0	0	HA	H20
LYS351	3.52	-0.86	1	0	HA	H3
ALA210	4.23	-0.47	0	0	HA	H2
GLU354	4.35	-0.36	-1	0	HB2	H3
THR236	4.66	-0.35	0	0	OG1	H21
MET329	4.70	-0.65	0	0	H	H17
LEU347	4.77	-0.95	0	0	O	H3
TYR332	4.81	-0.45	0	0	H	H8
ALA217	5.06	-0.30	0	0	H	H9
GLU208	5.14	-0.37	-1	0	O	H14
PHE326	5.21	-0.53	0	0	N	H23
VAL231	5.21	-0.33	0	0	HG11	H20
TRP214	5.24	-0.58	0	0	H	H11
ALA215	5.96	-0.22	0	0	HB3	H1
LYS323	6.01	-0.46	1	0	O	H6
PHE330	6.06	-0.16	0	0	H	H6
PHE211	6.13	-0.16	0	0	C	H14
ALA322	6.38	-0.08	0	0	O	H23
THR352	6.41	-0.01	0	0	H	H3
TYR353	6.44	-0.17	0	0	HB2	H3
SER220	6.52	-0.05	0	0	HG	H18
LYS233	6.70	-0.03	1	0	N	H20
LEU346	6.72	-0.13	0	0	O	H5
THR239	6.89	-0.04	0	0	HG1	H1
ALA229	6.91	-0.06	0	0	HA	H19
ARG348	6.95	-0.06	1	0	HA	H3
LEU349	6.98	0.02	0	0	C	H3
THR355	7.02	-0.07	0	0	H	H3
VAL482	7.26	-0.06	0	0	HG11	O3

LEU219	7.47	-0.05	0	0	HD13	H20
LEU234	7.61	0.00	0	0	C	H20
ARG218	7.68	-0.01	1	0	H	H9
GLU333	7.69	-0.06	-1	0	H	H8
GLY207	7.78	-0.04	0	0	O	H12
ASP237	7.93	0.02	-1	0	H	H21
VAL343	8.04	-0.03	0	0	HG13	H9
TYR319	8.04	-0.04	0	0	HE1	H3
ALA335	8.08	-0.01	0	0	HB1	H7
TYR334	8.20	-0.10	0	0	HB2	H7
PHE206	8.35	-0.01	0	0	O	H4
GLU230	8.50	0.01	-1	0	C	H20
LEU357	8.55	-0.04	0	0	HD13	H3
LEU238	8.96	-0.03	0	0	HB2	H1
ASN483	9.24	0.00	0	0	HD22	O3
VAL344	9.39	-0.01	0	0	O	O3
GLU227	9.46	0.00	-1	0	O	H20
THR356	9.50	0.00	0	0	H	H3
PHE377	9.58	-0.01	0	0	HE1	H3
PHE309	9.61	-0.11	0	0	HE2	H6
LEU481	9.70	-0.03	0	0	HD22	H4
LEU345	9.73	-0.01	0	0	O	H3
GLU358	9.81	-0.01	-1	0	HG3	H3
LYS240	9.85	0.03	1	0	H	H21

Supplementary Table 9 – Description of the quantum mechanics calculations of the conformation 2 of HSA-FA6::EST.

HSA Residue	Distance (Å)	Interaction Energy (kcal/mol)	Residue Charge	EST Charge	HSA Atom	EST Atom
ALA213	2.13	-4.32	0	0	HA	H9
SER232	2.15	-4.41	0	0	OG	H21
VAL216	2.17	-3.48	0	0	HG11	H8
LEU331	2.28	-2.08	0	0	HD23	H7
ARG209	2.40	-7.23	1	0	HG2	H12
LYS212	2.41	-6.09	1	0	HB2	H16
GLY328	2.43	-2.82	0	0	HA2	H6
LEU327	2.54	-3.05	0	0	HD11	H3
ASP324	2.62	-4.33	-1	0	HB2	H23
PHE228	2.69	-3.03	0	0	HZ	H17
GLU354	3.31	0.20	-1	0	OE1	H3
VAL235	3.65	-1.38	0	0	HG12	H1
ALA350	3.72	-0.54	0	0	HB1	H5
THR236	4.10	-0.43	0	0	HG1	H1
VAL231	4.14	-0.26	0	0	HG12	H20
LYS351	4.21	-0.20	1	0	HZ1	H3
VAL325	4.26	-0.64	0	0	HA	H22
LYS323	4.52	-0.86	1	0	HZ2	H4
LEU347	4.63	-0.34	0	0	HD23	H7
ALA210	5.22	-0.31	0	0	N	H11
TRP214	5.28	-0.42	0	0	N	H9
MET329	5.36	-0.44	0	0	N	H17
GLU208	5.40	-0.37	-1	0	O	H14
LYS233	5.67	-0.07	1	0	N	H21
ALA229	5.68	-0.15	0	0	HA	H19
TYR332	5.73	-0.33	0	0	HB3	H18
ALA217	5.81	-0.16	0	0	H	H8
ALA215	6.14	-0.21	0	0	H	H9
PHE211	6.23	-0.16	0	0	C	H9
PHE326	6.32	-0.29	0	0	N	H22
PHE330	6.41	-0.17	0	0	H	H6
THR239	6.46	-0.06	0	0	HG1	H1
SER220	6.85	-0.04	0	0	HG	H18
TYR353	6.88	-0.16	0	0	HB2	H3
THR355	7.12	-0.05	0	0	H	H3
LEU234	7.17	-0.04	0	0	H	H20
LEU346	7.20	-0.06	0	0	O	H7
ALA322	7.22	-0.07	0	0	HB3	H23
THR352	7.49	-0.01	0	0	N	H3
GLU230	7.56	-0.02	-1	0	O	H20
LEU357	7.57	-0.06	0	0	HD12	H3

TYR319	7.59	-0.03	0	0	HE1	O3
PHE206	8.02	-0.02	0	0	O	H2
LEU219	8.03	-0.04	0	0	HB3	H18
LEU349	8.07	-0.02	0	0	O	H3
ASP237	8.11	0.02	-1	0	H	H1
GLU358	8.16	0.03	-1	0	OE1	O3
VAL482	8.20	-0.02	0	0	HG13	H3
GLU227	8.38	-0.03	-1	0	O	H20
ARG218	8.51	0.01	1	0	H	H8
GLY207	8.58	-0.05	0	0	O	H11
GLU333	8.61	-0.06	-1	0	H	H6
ARG348	8.65	0.02	1	0	O	H3
LEU238	8.66	-0.04	0	0	HB2	H1
VAL343	8.73	-0.02	0	0	HG13	H7
ALA335	8.78	-0.01	0	0	HB1	H7
THR356	9.58	-0.01	0	0	H	H3
TYR334	9.65	-0.09	0	0	H	H6
LYS240	9.75	0.01	1	0	H	H1

Supplementary Table 10 – Description of the quantum mechanics calculations of the conformation 3 of HSA-FA6::EST.

HSA Residue	Distance (Å)	Interaction Energy (kcal/mol)	Residue Charge	EST Charge	HSA Atom	EST Atom
THR236	1.97	-5.23	0	0	OG1	H21
ASP324	2.02	-6.19	-1	0	O	H3
SER232	2.04	-4.77	0	0	HB3	H16
VAL216	2.21	-3.54	0	0	HG21	H12
LYS212	2.23	-5.00	1	0	HD3	H15
GLY328	2.25	-1.14	0	0	H	H3
VAL235	2.36	-3.58	0	0	HG12	H15
PHE228	2.61	-4.70	0	0	HZ	H9
VAL325	2.65	-4.35	0	0	HA	H5
ALA213	2.73	-1.93	0	0	HA	H4
LEU327	2.99	-2.67	0	0	HB3	H3
LEU331	3.60	-0.97	0	0	HD23	H4
LYS233	3.90	-0.84	1	0	N	H1
ARG209	4.59	-0.84	1	0	O	H4
ALA215	4.72	-0.34	0	0	HB2	H12
MET329	4.74	-0.48	0	0	H	H5
VAL231	4.95	-0.36	0	0	O	H14
PHE326	5.22	-0.45	0	0	N	H5
ASP237	5.28	-0.23	-1	0	H	H21
ALA229	5.29	-0.34	0	0	HA	H18
THR239	5.36	-0.18	0	0	HG1	H15
LYS323	5.43	0.01	1	0	O	H3
GLU208	5.46	-0.23	-1	0	HG2	H23
LEU347	5.80	-0.09	0	0	HD23	H4
LEU234	5.82	-0.23	0	0	H	H1
TRP214	5.91	-0.22	0	0	N	H4
ALA210	6.14	-0.07	0	0	HA	H4
ALA217	6.22	-0.14	0	0	H	H2
TYR332	6.33	-0.29	0	0	HB3	H11
PHE330	6.40	-0.14	0	0	H	H3
ALA350	6.41	-0.04	0	0	HB2	O3
ALA322	6.57	-0.08	0	0	O	H5
LEU219	6.66	-0.09	0	0	HD11	H14
PHE211	6.71	-0.21	0	0	C	H2
TYR319	7.13	-0.03	0	0	HE1	H3
GLU354	7.17	0.10	-1	0	OE2	H3
LEU238	7.18	-0.11	0	0	H	H21
GLU230	7.39	-0.11	-1	0	O	H1
LYS240	7.40	-0.03	1	0	H	H21
ARG218	8.37	-0.04	1	0	H	H12
SER220	8.41	-0.04	0	0	HB3	H12

LEU203	8.71	-0.02	0	0	HD13	H15
GLU227	8.79	-0.09	-1	0	O	H16
TYR353	8.84	-0.11	0	0	HD2	H3
LYS351	9.02	-0.03	1	0	HA	O3
LEU357	9.26	-0.04	0	0	HD11	H3
GLY207	9.35	-0.02	0	0	O	H23
GLU333	9.40	-0.06	-1	0	H	H3
LEU346	9.57	-0.01	0	0	O	H4
ALA335	9.81	-0.01	0	0	HB2	H4
VAL241	9.99	-0.01	0	0	H	H21
LEU260	9.99	-0.03	0	0	HD13	H21

Supplementary Table 11 – Description of the quantum mechanics calculations of the conformation 4 of HSA-FA6::EST.

HSA Residue	Distance (Å)	Interaction Energy (kcal/mol)	Residue Charge	EST Charge	HSA Atom	EST Atom
GLY328	2.00	0.41	0	0	HA2	H8
ARG209	2.02	-7.70	1	0	HG3	H13
ASP324	2.05	-3.28	-1	0	HB2	H23
ALA213	2.06	-3.74	0	0	HB3	H2
LYS212	2.27	-6.60	1	0	HE3	H15
LEU331	2.36	-4.18	0	0	HB3	H7
LEU327	2.39	-3.44	0	0	HB2	H6
VAL216	2.40	-3.63	0	0	HG22	H16
VAL325	2.72	-2.76	0	0	HA	H17
PHE228	2.74	-1.41	0	0	HZ	H18
ALA350	2.79	-0.93	0	0	HB3	H5
LYS351	3.31	-0.56	1	0	HE2	O3
GLU354	3.37	-0.37	-1	0	OE2	H3
LEU347	4.23	-1.28	0	0	HD22	C3
SER232	4.47	-0.39	0	0	OG	H20
ALA210	4.53	-0.35	0	0	HA	H2
GLU208	4.59	-0.41	-1	0	O	H15
MET329	4.60	-0.58	0	0	H	H8
VAL235	4.71	-0.32	0	0	HG13	H1
TYR332	5.05	-0.37	0	0	H	H7
PHE330	5.17	-0.31	0	0	HB2	H7
LYS323	5.27	-0.54	1	0	O	H6
THR236	5.43	-0.19	0	0	OG1	H21
PHE326	5.51	-0.30	0	0	C	H6
TRP214	5.55	-0.36	0	0	H	H12
ALA217	5.67	-0.25	0	0	H	H11
PHE211	6.01	-0.20	0	0	C	H12
VAL482	6.01	-0.09	0	0	HG23	H4
ALA215	6.32	-0.14	0	0	H	H14
LEU346	6.47	-0.16	0	0	O	H5
THR352	6.73	-0.02	0	0	H	H3
TYR353	6.87	-0.14	0	0	HD2	H5
ALA322	6.93	-0.08	0	0	O	H17
LEU349	6.94	0.01	0	0	C	H3
THR239	7.03	-0.04	0	0	HG21	H15
ARG348	7.07	-0.07	1	0	N	O3
THR355	7.10	-0.07	0	0	HG22	H3
TYR319	7.34	-0.06	0	0	HE1	H3
GLY207	7.53	-0.06	0	0	O	H12
SER220	7.56	-0.03	0	0	HG	H9
LEU481	7.74	-0.04	0	0	HD23	H4

GLU333	7.92	-0.07	-1	0	H	H7
ALA229	7.97	-0.05	0	0	HA	H20
TYR334	7.99	-0.11	0	0	HB3	H7
ALA335	7.99	-0.01	0	0	HB2	H7
LYS233	8.09	0.02	1	0	N	H21
VAL231	8.13	-0.05	0	0	O	H1
ARG218	8.54	-0.03	1	0	H	H11
PHE206	8.60	-0.01	0	0	O	H2
PHE309	8.62	-0.10	0	0	HZ	H7
SER202	8.86	-0.01	0	0	HB2	H2
LEU357	8.97	-0.03	0	0	HD11	H3
LEU203	9.02	-0.02	0	0	HD13	H15
VAL344	9.03	-0.01	0	0	O	O3
VAL343	9.40	-0.02	0	0	O	O3
ASP237	9.47	0.07	-1	0	H	H21
LEU219	9.49	-0.03	0	0	H	H9
LEU234	9.57	-0.01	0	0	C	H1
THR356	9.63	0.00	0	0	H	H3
ASN483	9.66	0.00	0	0	HD22	O3
LEU345	9.76	-0.01	0	0	O	H3
GLU358	9.78	-0.02	-1	0	HG2	H3
PHE377	9.86	-0.01	0	0	HE1	H3
LEU238	9.90	-0.03	0	0	HD23	H14

Supplementary Table 12 – Description of the quantum mechanics calculations of the conformation 5 of HSA-FA6::EST.

HSA Residue	Distance (Å)	Interaction Energy (kcal/mol)	Residue Charge	EST Charge	HSA Atom	EST Atom
GLU354	1.89	-8.06	-1	0	OE2	H3
VAL216	2.12	-2.27	0	0	HG22	H20
GLY328	2.29	-3.36	0	0	HA2	H8
ASP324	2.34	-3.86	-1	0	HB2	H22
ALA213	2.37	-5.01	0	0	HB2	H9
LYS351	2.37	-1.57	1	0	HA	O3
LEU331	2.39	-4.02	0	0	HD22	H7
LEU327	2.42	-6.39	0	0	HB2	H6
ARG209	2.48	-6.62	1	0	HH22	H12
ALA350	2.77	-1.59	0	0	HB2	H5
LYS212	2.96	-2.90	1	0	HB2	H1
LYS323	3.53	-0.70	1	0	HZ2	H4
LEU347	4.22	-0.80	0	0	HD22	H7
PHE228	4.51	-0.53	0	0	HZ	H19
VAL325	4.53	-0.37	0	0	N	H24
VAL235	4.64	-0.19	0	0	HG11	H20
ALA210	4.77	-0.28	0	0	N	H14
MET329	4.90	-0.50	0	0	N	H17
PHE330	4.98	-0.39	0	0	HD1	H6
THR355	5.31	-0.15	0	0	H	H3
TYR353	5.32	-0.36	0	0	HB2	H5
TYR332	5.38	-0.33	0	0	H	H8
ALA217	5.40	-0.16	0	0	H	H18
GLU208	5.43	-0.46	-1	0	O	H1
TRP214	5.44	-0.43	0	0	N	H9
THR352	5.52	-0.29	0	0	N	O3
ALA215	5.93	-0.11	0	0	HB1	H20
PHE326	5.94	-0.40	0	0	C	H6
PHE211	6.16	-0.11	0	0	C	H1
LEU346	6.31	-0.10	0	0	O	H7
SER232	6.33	-0.08	0	0	OG	H20
LEU349	6.44	-0.05	0	0	C	H5
TYR319	6.47	-0.09	0	0	HE1	H3
VAL231	6.70	-0.07	0	0	HG12	H20
LEU357	6.71	-0.09	0	0	HD13	H3
ARG348	7.08	-0.05	1	0	O	O3
SER220	7.44	-0.02	0	0	HG	H18
GLU358	7.63	-0.04	-1	0	HG2	H3
THR356	7.69	-0.03	0	0	H	H3
GLY207	7.75	-0.06	0	0	O	H14
ALA322	7.77	-0.04	0	0	O	H24

TYR334	7.84	-0.11	0	0	HD1	H7
ARG218	7.96	0.02	1	0	H	H18
VAL482	7.98	-0.05	0	0	HG23	H9
PHE206	8.05	-0.02	0	0	O	H14
VAL343	8.09	-0.02	0	0	HG13	H9
GLU333	8.12	-0.06	-1	0	H	H8
THR239	8.16	0.00	0	0	HG1	H21
ALA335	8.42	-0.01	0	0	HB1	H8
THR236	8.43	-0.07	0	0	OG1	H21
PHE377	8.43	-0.02	0	0	HZ	H5
LEU219	9.07	-0.03	0	0	H	H18
LEU238	9.58	-0.02	0	0	HD21	H20
ALA229	9.60	-0.03	0	0	HA	H19
LEU345	9.63	0.00	0	0	O	H7
LYS233	9.63	0.05	1	0	N	H20
LEU203	9.67	-0.01	0	0	HD11	H1
SER202	9.84	-0.01	0	0	HG	H14
ASN483	9.85	0.01	0	0	HD22	H3
SER480	9.89	-0.05	0	0	HG	H4
PHE309	9.92	-0.09	0	0	HZ	H6

Supplementary Table 13 – Description of the quantum mechanics calculations of the final conformation of HSA-FA6::EST.

HSA Residue	Distance (Å)	Interaction Energy (kcal/mol)	Residue Charge	EST Charge	HSA Atom	EST Atom
GLY328	2.06	-2.58	0	0	HA2	H8
ALA213	2.24	-5.48	0	0	HB3	H11
ARG209	2.27	-9.08	1	0	HG2	H15
LEU331	2.31	-3.22	0	0	HD23	H7
VAL216	2.40	-3.35	0	0	HG11	H9
ASP324	2.58	-5.42	-1	0	HB2	H23
LEU327	2.67	-3.36	0	0	HB1	H6
LYS212	2.71	-5.19	1	0	HB2	H1
LYS351	2.90	-1.44	1	0	HE2	H3
ALA350	2.92	-1.26	0	0	HB1	H5
SER232	3.36	-0.75	0	0	HB1	H20
GLU354	3.56	-0.97	-1	0	OE2	H3
PHE228	3.57	-0.96	0	0	HZ	H17
VAL325	3.68	-1.63	0	0	HA	H17
VAL235	3.76	-0.85	0	0	HG12	H1
ALA210	4.22	-0.57	0	0	HA	H2
LEU347	4.42	-0.93	0	0	HD23	H5
THR236	5.22	-0.32	0	0	OG1	H21
GLU208	5.24	-0.17	-1	0	O	H14
MET329	5.29	-0.37	0	0	N	H8
ALA217	5.32	-0.23	0	0	HN	H9
LYS323	5.39	-0.83	1	0	O	H6
TRP214	5.50	-0.43	0	0	HN	H11
TYR332	5.60	-0.21	0	0	HN	H8
PHE326	5.86	-0.27	0	0	C	H6
PHE330	5.88	-0.22	0	0	HD1	H7
VAL231	6.21	-0.10	0	0	HG12	H20
PHE211	6.35	-0.18	0	0	C	H11
ALA215	6.61	-0.12	0	0	HN	H11
LEU346	6.69	-0.11	0	0	O	H5
THR352	6.76	-0.03	0	0	HN	H3
THR355	6.92	-0.03	0	0	HN	H3
LYS233	6.99	-0.07	1	0	N	H20
VAL482	7.07	-0.07	0	0	HG23	O3
ALA229	7.14	-0.05	0	0	HA	H20
ARG348	7.16	-0.11	1	0	HA	H3
SER220	7.21	-0.03	0	0	HG1	H18
PHE206	7.24	-0.05	0	0	O	H2
TYR353	7.27	-0.03	0	0	HB2	H5
THR239	7.45	-0.03	0	0	OG1	H1
ALA322	7.46	-0.07	0	0	HB2	H22

LEU349	7.52	0.03	0	0	C	H5
TYR319	7.78	-0.03	0	0	HE1	H3
GLY207	7.84	-0.04	0	0	O	H2
ARG218	8.30	-0.04	1	0	HN	H9
ALA335	8.34	-0.01	0	0	HB2	H7
GLU333	8.45	-0.04	-1	0	HN	H8
TYR334	8.55	-0.02	0	0	HB1	H7
LEU357	8.77	-0.01	0	0	HD11	H3
LEU219	8.80	-0.02	0	0	HB1	H18
SER480	8.80	-0.02	0	0	HB1	H4
LEU234	8.93	0.00	0	0	HN	H20
ASP237	9.10	-0.01	-1	0	HN	H21
VAL343	9.12	-0.02	0	0	HG12	H7
GLU230	9.19	-0.03	-1	0	C	H20
VAL344	9.46	-0.02	0	0	HA	O3
LEU203	9.49	-0.02	0	0	HD12	H14
THR356	9.56	0.00	0	0	HN	H3
SER202	9.57	0.00	0	0	HB1	H2
LEU481	9.64	-0.04	0	0	HB2	H4
LEU238	9.88	-0.01	0	0	HD22	H1
LEU345	9.88	-0.01	0	0	O	H5
PHE309	9.90	-0.01	0	0	HE1	H6

Supplementary Table 14 – Description of the quantum mechanics calculations of the conformation 0 of HSA-FA1::DHT.

HSA Residue	Distance (Å)	Interaction Energy (kcal/mol)	Residue Charge	DHT Charge	HSA Atom	DHT Atom
PRO118	2.06	-4.48	0	0	HG2	H17
PHE134	2.10	-4.77	0	0	HE1	H23
LYS137	2.16	-4.68	1	0	HB2	H20
LEU182	2.16	-2.43	0	0	HD11	H4
LEU115	2.37	-4.89	0	0	HD12	H8
ARG117	2.40	-5.33	1	0	HG2	H3
TYR138	2.43	-6.71	0	0	HD1	H6
TYR161	2.57	-4.74	0	0	OH	H7
MET123	2.58	-4.44	0	0	HG2	H17
ILE142	2.61	-2.08	0	0	HD11	H6
GLU141	2.92	-3.03	-1	0	HG3	H10
VAL116	3.26	-1.08	0	0	O	H30
ALA126	3.39	-0.79	0	0	HB1	H24
PHE165	3.67	-1.16	0	0	HE2	H2
VAL122	4.59	-0.41	0	0	HG13	H17
ARG186	4.66	-0.58	1	0	HB3	O3
LEU185	4.79	-0.35	0	0	HB3	O3
ASN130	4.92	-0.35	0	0	HD21	H24
THR133	5.07	-0.28	0	0	O	H22
LEU139	5.70	-0.36	0	0	N	H9
LEU179	5.79	-0.11	0	0	HD22	H4
LEU178	5.90	-0.19	0	0	HD22	H2
LEU135	5.97	-0.28	0	0	HA	H11
ARG145	6.07	-0.01	1	0	HH11	H8
LYS136	6.12	-0.08	1	0	C	H20
PHE127	6.17	-0.21	0	0	N	H23
ASP183	6.35	0.02	-1	0	N	O3
TYR140	6.43	-0.21	0	0	H	H10
LYS181	6.52	-0.06	1	0	O	O3
GLU119	6.65	-0.22	-1	0	O	H17
CYS124	6.86	-0.12	0	0	N	H16
VAL120	7.01	-0.15	0	0	HA	H14
ARG114	7.09	0.01	1	0	C	H28
ALA143	7.34	-0.04	0	0	H	H9
PHE36	7.35	-0.16	0	0	HE2	H22
ARG144	7.44	0.09	1	0	HH12	H19
THR125	7.80	-0.03	0	0	C	H24
PHE157	7.96	-0.02	0	0	HB2	H6
GLU184	7.98	0.12	-1	0	H	O3
ASP187	8.06	0.00	-1	0	H	O3
ALA158	8.06	-0.02	0	0	HA	H6

LEU154	8.09	-0.04	0	0	HD12	H9
ALA164	8.12	-0.04	0	0	HB3	O3
GLY189	8.17	-0.03	0	0	HA3	O3
GLU132	8.30	0.02	-1	0	O	H22
GLU131	8.48	-0.02	-1	0	O	H22
ASP129	8.57	-0.09	-1	0	HB3	H24
PRO113	8.60	-0.02	0	0	O	H28
HIS146	8.62	-0.02	0	0	HD2	H9
LYS190	8.66	0.04	1	0	HE3	H5
HIS128	8.71	-0.06	0	0	H	H23
ASP121	8.72	-0.03	-1	0	C	H17
PRO180	9.09	0.00	0	0	N	H4
LYS162	9.22	0.07	1	0	N	H7
ARG160	9.35	0.04	1	0	C	O3
GLU37	9.43	0.00	-1	0	OE2	H21
LEU112	9.43	-0.02	0	0	HD13	H8
PHE149	9.45	-0.02	0	0	HE2	H6
GLU188	9.53	0.10	-1	0	H	O3
LEU24	9.83	-0.01	0	0	HD21	H20
ALA175	9.91	0.02	0	0	HA	H4
CYS169	9.99	-0.02	0	0	SG	H16

Supplementary Table 15 – Description of the quantum mechanics calculations of the conformation 1 of HSA-FA1::DHT.

HSA Residue	Distance (Å)	Interaction Energy (kcal/mol)	Residue Charge	DHT Charge	HSA Atom	DHT Atom
LEU182	1.91	-2.04	0	0	HD23	H4
TYR138	2.04	-6.67	0	0	HD1	H9
PRO118	2.21	-4.12	0	0	HD2	H15
LEU115	2.23	-5.39	0	0	HD21	H8
PHE134	2.25	-4.64	0	0	HE1	H16
GLU141	2.28	-4.53	-1	0	HB3	H10
LYS137	2.50	-4.13	1	0	HB2	H20
TYR161	2.55	-4.72	0	0	HH	H13
ARG117	2.66	-4.56	1	0	HG2	H3
VAL116	2.81	-2.29	0	0	O	H28
ILE142	3.05	-1.87	0	0	HD12	H6
MET123	3.70	-2.24	0	0	SD	H14
PHE165	4.18	-0.78	0	0	HZ	H2
ALA126	4.59	-0.33	0	0	HB1	H24
LEU185	4.65	-0.42	0	0	HB3	O3
ARG186	5.00	-0.53	1	0	HB3	O3
LEU139	5.37	-0.48	0	0	N	H11
ARG145	5.46	-0.15	1	0	HH11	H8
LEU178	5.63	-0.16	0	0	HD12	H4
LEU179	5.80	-0.12	0	0	HD21	H4
VAL122	5.80	-0.17	0	0	HG13	H17
TYR140	5.86	-0.31	0	0	HB2	H20
LEU135	5.97	-0.33	0	0	O	H11
ASN130	6.14	-0.29	0	0	HB3	H24
THR133	6.25	-0.15	0	0	O	H22
LYS136	6.41	0.02	1	0	C	H20
ARG114	6.42	-0.02	1	0	O	H29
ASP183	6.44	-0.01	-1	0	N	H4
PHE127	6.44	-0.15	0	0	HB3	H16
LYS181	6.50	-0.06	1	0	O	O3
ARG144	6.64	0.03	1	0	HD3	H10
ALA143	6.83	-0.05	0	0	H	H9
PHE36	7.14	-0.17	0	0	HD1	H20
PHE157	7.17	-0.03	0	0	HD1	O3
GLU119	7.44	-0.17	-1	0	N	H17
CYS124	7.83	-0.06	0	0	N	H17
ALA158	7.94	-0.03	0	0	HA	O3
HIS146	7.98	-0.02	0	0	HE2	H6
VAL120	7.99	-0.11	0	0	HA	H14
GLY189	8.00	-0.03	0	0	HA3	H6
LYS190	8.02	0.05	1	0	HE3	H6

GLU184	8.03	0.10	-1	0	C	O3
ASP187	8.07	-0.02	-1	0	H	O3
ALA164	8.20	-0.04	0	0	HB2	O3
LEU154	8.28	-0.03	0	0	HD11	H9
PRO113	8.32	-0.02	0	0	O	H29
GLU37	8.71	-0.02	-1	0	OE1	H22
ASP129	8.76	-0.08	-1	0	HB3	H24
GLU131	8.77	0.01	-1	0	O	H23
PRO180	9.04	-0.01	0	0	C	H4
THR125	9.05	-0.04	0	0	C	H17
LEU112	9.11	-0.03	0	0	HD13	H8
GLU188	9.15	0.11	-1	0	H	O3
LYS162	9.22	0.07	1	0	N	O3
GLU132	9.22	0.05	-1	0	O	H23
ARG160	9.30	0.04	1	0	C	O3
ASP121	9.59	-0.01	-1	0	C	H17
HIS128	9.79	-0.06	0	0	H	H16
PHE149	9.86	-0.02	0	0	HE2	H6
LEU24	9.92	-0.01	0	0	HD22	H20

Supplementary Table 16 – Description of the quantum mechanics calculations of the conformation 2 of HSA-FA1::DHT.

HSA Residue	Distance (Å)	Interaction Energy (kcal/mol)	Residue Charge	DHT Charge	HSA Atom	DHT Atom
ASN130	1.77	-5.68	0	0	OD1	H24
LEU115	1.96	-3.00	0	0	HD11	H5
PRO118	2.03	-3.70	0	0	HG2	H14
TYR138	2.03	-3.88	0	0	HD1	H6
LYS137	2.14	-4.43	1	0	HG2	H19
TYR161	2.21	-3.48	0	0	HH	O3
MET123	2.40	-3.27	0	0	HE2	H4
PHE134	2.40	-10.03	0	0	HD1	H23
ARG117	2.49	-2.14	1	0	HG2	H3
ALA126	2.66	-2.64	0	0	HB1	H23
LEU182	2.89	-0.89	0	0	HD21	O3
VAL116	3.14	-1.27	0	0	O	H3
PHE165	3.30	-1.16	0	0	HZ	H2
GLU141	3.48	-1.29	-1	0	HG3	H8
THR133	4.12	-0.63	0	0	O	H20
ASP129	4.72	-0.34	-1	0	HB2	H24
PHE127	4.95	-0.52	0	0	N	H23
VAL122	5.15	-0.26	0	0	HG12	H17
LEU135	5.26	-0.07	0	0	N	H11
ILE142	5.57	-0.20	0	0	HD12	H6
LEU178	5.78	-0.15	0	0	HD21	H4
PHE36	5.84	-0.28	0	0	HE2	H19
LEU139	5.86	-0.21	0	0	H	H9
LYS136	6.06	0.05	1	0	C	H10
LEU179	6.25	-0.08	0	0	HD23	H4
TYR140	6.30	-0.24	0	0	HD2	H8
GLU131	6.61	-0.23	-1	0	N	H22
LEU185	6.73	-0.08	0	0	HD12	O3
THR125	6.91	-0.10	0	0	O	H24
ARG186	6.97	-0.17	1	0	HB3	O3
ARG114	7.08	0.06	1	0	O	H28
ARG145	7.08	0.06	1	0	HH21	H5
CYS124	7.17	-0.14	0	0	N	H16
GLU119	7.23	-0.20	-1	0	N	H1
VAL120	7.36	-0.13	0	0	HG22	H4
HIS128	7.42	-0.10	0	0	N	H23
PHE157	7.51	-0.02	0	0	HE2	H6
ASP183	7.56	0.12	-1	0	N	O3
GLU132	7.78	-0.02	-1	0	C	H22
LYS181	8.23	-0.05	1	0	C	O3
ARG144	8.23	0.12	1	0	HH12	H8

GLU37	8.60	0.02	-1	0	HG3	H19
ALA164	8.65	-0.02	0	0	HB1	O3
ALA158	8.80	0.00	0	0	HA	H6
LEU24	8.85	-0.02	0	0	HD22	H10
PRO113	8.97	-0.01	0	0	O	H5
ASP121	9.02	-0.02	-1	0	O	H16
ALA143	9.05	-0.01	0	0	H	H9
LYS162	9.38	0.09	1	0	HA	H7
CYS169	9.51	-0.02	0	0	SG	H2
VAL40	9.70	-0.03	0	0	HG23	H20
GLU184	9.75	0.12	-1	0	H	O3
LEU154	9.98	-0.03	0	0	HD11	H9

Supplementary Table 17 – Description of the quantum mechanics calculations of the conformation 3 of HSA-FA1::DHT.

HSA Residue	Distance (Å)	Interaction Energy (kcal/mol)	Residue Charge	DHT Charge	HSA Atom	DHT Atom
TYR138	2.03	-6.91	0	0	HD1	H11
LEU115	2.08	-6.09	0	0	HD21	H8
ARG117	2.18	-3.93	1	0	HE	H3
LEU182	2.19	-2.37	0	0	HD11	H4
LYS137	2.19	-3.89	1	0	HB3	H22
TYR161	2.35	-5.12	0	0	HE1	H2
PHE134	2.53	-4.00	0	0	HD1	H23
GLU141	2.65	-3.97	-1	0	HG3	H10
MET123	2.69	-3.76	0	0	HG2	H17
PHE165	2.77	-1.86	0	0	HZ	H2
PRO118	3.10	-2.08	0	0	HD3	H27
ALA126	3.15	-0.76	0	0	HB2	H24
ILE142	3.23	-1.30	0	0	HD13	H6
VAL116	3.35	-1.41	0	0	O	H30
THR133	4.41	-0.31	0	0	O	H22
LEU185	4.71	-0.41	0	0	HB2	O3
ARG186	4.95	-0.42	1	0	HB3	O3
LEU178	5.50	-0.22	0	0	HD21	H2
ARG145	5.52	-0.07	1	0	HH11	H10
VAL122	5.63	-0.24	0	0	HG12	H17
PHE157	5.66	-0.15	0	0	HZ	O3
TYR140	5.66	-0.41	0	0	HD2	H20
LEU135	5.70	-0.27	0	0	N	H23
PHE127	5.74	-0.23	0	0	HB3	H16
LEU139	5.98	-0.37	0	0	N	H20
LEU179	6.07	-0.13	0	0	HD21	H4
LYS136	6.13	-0.03	1	0	C	H20
CYS124	6.18	-0.15	0	0	N	H17
VAL120	6.60	-0.20	0	0	HA	H14
GLU119	6.63	-0.11	-1	0	O	H17
ASP183	6.63	0.06	-1	0	N	O3
ARG114	6.78	0.00	1	0	C	H30
ASN130	6.87	-0.17	0	0	O	H24
ARG144	7.00	0.09	1	0	HH12	H19
LYS181	7.00	-0.02	1	0	O	O3
PHE36	7.06	-0.18	0	0	CE1	H22
GLU131	7.32	-0.01	-1	0	O	H23
THR125	7.39	-0.05	0	0	H	H17
HIS146	7.40	-0.03	0	0	HE2	H6
ALA143	7.50	-0.03	0	0	H	H9
ALA164	7.54	-0.03	0	0	HB3	O3

ASP129	7.64	-0.08	-1	0	HB2	H24
ALA158	7.81	-0.01	0	0	HA	H7
LEU154	7.81	-0.03	0	0	HD12	H9
GLU132	7.93	-0.02	-1	0	O	H22
GLY189	8.09	-0.03	0	0	HA3	H6
GLU184	8.18	0.08	-1	0	H	O3
PRO113	8.45	-0.02	0	0	O	H8
ASP121	8.50	-0.01	-1	0	C	H17
ASP187	8.52	0.01	-1	0	H	O3
LYS162	8.55	0.06	1	0	HA	H2
HIS128	8.62	-0.05	0	0	H	H24
LEU24	8.64	-0.02	0	0	HD21	H20
PRO180	8.87	0.00	0	0	N	H4
ARG160	9.03	0.04	1	0	C	O3
CYS169	9.21	-0.02	0	0	SG	H16
LYS190	9.41	0.06	1	0	H	H6
LEU112	9.52	-0.02	0	0	HD11	H8
PHE149	9.60	-0.02	0	0	HE2	H6
CYS168	9.97	-0.06	0	0	HB2	H4
GLU188	9.99	0.11	-1	0	H	O3

Supplementary Table 18 – Description of the quantum mechanics calculations of the final conformation of HSA-FA1::DHT.

HSA Residue	Distance (Å)	Interaction Energy (kcal/mol)	Residue Charge	DHT Charge	HSA Atom	DHT Atom
LYS137	2.11	-4.35	1	0	HB2	H20
LEU115	2.25	-6.36	0	0	HB2	H29
PHE134	2.29	-5.76	0	0	HE1	H16
PRO118	2.32	-4.87	0	0	HG2	H17
MET123	2.40	-4.70	0	0	HG2	H14
TYR138	2.41	-5.71	0	0	HB1	H11
TYR161	2.42	-4.17	0	0	HH	H13
ARG117	2.43	-5.86	1	0	HG1	H3
LEU182	2.45	-1.89	0	0	HD11	H4
VAL116	2.52	-2.03	0	0	O	H30
ALA126	2.78	-1.32	0	0	HB2	H24
GLU141	3.13	-3.04	-1	0	HG1	H10
ILE142	3.27	-1.26	0	0	HD2	H6
PHE165	3.92	-1.08	0	0	HZ	H2
THR133	4.24	-0.59	0	0	HG23	H22
ARG186	4.59	-1.03	1	0	HB1	O3
VAL122	5.00	-0.40	0	0	HG13	H17
LEU185	5.57	-0.17	0	0	HB1	O3
LEU135	5.63	-0.36	0	0	HA	H11
LEU139	5.76	-0.38	0	0	N	H10
PHE127	5.84	-0.24	0	0	HN	H23
LEU179	5.95	-0.10	0	0	HD22	H4
ARG145	5.98	-0.18	1	0	HH11	H8
LYS136	5.98	-0.12	1	0	C	H20
GLU119	6.12	-0.29	-1	0	O	H14
LEU178	6.14	-0.14	0	0	HD21	H2
ASP183	6.16	0.21	-1	0	N	O3
TYR140	6.25	-0.30	0	0	HB2	H10
CYS124	6.40	-0.17	0	0	N	H16
VAL120	6.66	-0.10	0	0	HA	H14
THR125	6.69	-0.06	0	0	HG22	H24
ARG114	6.90	0.05	1	0	C	H29
ASN130	7.28	-0.10	0	0	O	H23
ASP129	7.29	-0.09	-1	0	HB2	H24
PHE36	7.42	-0.12	0	0	CE1	H20
ALA143	7.46	-0.03	0	0	HN	H9
LYS181	7.47	-0.15	1	0	O	O3
ARG144	7.59	-0.02	1	0	HD1	H8
GLU132	7.80	-0.12	-1	0	O	H22
GLU131	7.92	-0.12	-1	0	O	H23
ASP121	8.14	-0.06	-1	0	O	H17

LEU154	8.17	-0.02	0	0	HD12	H9
HIS146	8.17	-0.04	0	0	HD2	H6
ASP187	8.24	0.11	-1	0	HN	O3
PRO113	8.30	-0.04	0	0	O	H28
ALA158	8.30	-0.02	0	0	HA	H11
GLU184	8.36	0.03	-1	0	N	O3
HIS128	8.49	-0.04	0	0	HN	H24
PHE157	8.63	-0.02	0	0	HB2	H6
GLY189	8.76	-0.04	0	0	HA1	O3
ALA164	8.83	-0.03	0	0	HB1	H2
LEU24	8.99	-0.02	0	0	HD21	H20
LYS190	9.14	-0.13	1	0	HE1	O3
CYS169	9.14	-0.02	0	0	SG	H16
PRO180	9.45	0.00	0	0	N	H4
LYS162	9.60	0.00	1	0	N	H11
LEU112	9.64	-0.01	0	0	HD12	H8
PHE149	9.92	-0.02	0	0	HE2	H6

Supplementary Table 19 – Description of the quantum mechanics calculations of the conformation 0 of HSA-FA6::DHT.

HSA Residue	Distance (Å)	Interaction Energy (kcal/mol)	Residue Charge	DHT Charge	HSA Atom	DHT Atom
LEU481	2.03	-7.30	0	0	H	O17
LYS351	2.24	-6.62	1	0	HD3	H15
VAL482	2.27	-4.74	0	0	HG21	H17
LEU327	2.29	-5.17	0	0	HB2	H3
ALA213	2.36	-3.55	0	0	HB2	H1
ALA350	2.48	-2.34	0	0	HB2	H4
LEU347	2.53	-4.40	0	0	HD21	H1
PHE206	2.60	-3.92	0	0	HB2	H20
ALA210	2.61	-3.12	0	0	HA	H11
ARG209	2.67	-6.05	1	0	HB3	H9
SER480	2.76	-3.04	0	0	HA	H24
ASP324	2.78	-1.84	-1	0	HB2	H5
LEU331	2.98	-1.48	0	0	HD22	H4
GLY328	3.08	-0.68	0	0	HA2	O3
GLU354	3.98	-1.39	-1	0	HB3	H28
GLU479	4.23	-0.71	-1	0	O	H24
ASN483	4.67	-0.53	0	0	H	O17
ARG348	4.93	-0.12	1	0	HA	H17
VAL325	5.30	0.04	0	0	HA	O3
PHE211	5.82	-0.33	0	0	N	H11
TRP214	5.83	-0.31	0	0	N	H1
TYR353	5.85	-0.26	0	0	HB2	H3
THR352	5.90	-0.28	0	0	H	H14
MET329	5.93	-0.11	0	0	H	O3
LEU346	6.01	-0.22	0	0	O	H2
LYS205	6.02	-0.04	1	0	O	H22
VAL216	6.11	-0.14	0	0	HB	O3
PHE326	6.16	-0.22	0	0	C	O3
LYS212	6.19	-0.11	1	0	HB3	H6
PHE330	6.22	-0.20	0	0	HB2	H4
TYR319	6.29	-0.10	0	0	HH	H30
GLY207	6.33	-0.19	0	0	N	H20
THR355	6.37	-0.13	0	0	H	H28
PHE228	6.48	-0.12	0	0	HE1	O3
SER202	6.56	-0.11	0	0	HA	H22
THR478	6.59	-0.11	0	0	O	H21
LEU349	6.60	-0.11	0	0	C	H2
ARG484	6.60	-0.05	1	0	HG3	O17
LYS323	6.65	-0.15	1	0	O	H5
VAL344	6.74	-0.11	0	0	HA	H16
GLU208	6.76	-0.20	-1	0	C	H9

ALA217	6.84	-0.04	0	0	HB2	H4
TYR332	7.11	-0.13	0	0	H	H4
VAL343	7.34	-0.09	0	0	HG13	H1
CYS477	7.46	-0.02	0	0	O	H22
CYS476	7.87	0.01	0	0	O	O17
ALA215	7.95	-0.06	0	0	H	H7
ALA201	8.13	-0.02	0	0	O	H22
LEU345	8.46	-0.04	0	0	O	H14
LEU203	8.46	-0.04	0	0	HA	H20
LEU357	8.49	-0.03	0	0	HD11	H3
TYR334	8.58	-0.10	0	0	HB2	H4
ALA322	8.60	-0.01	0	0	O	H5
ALA335	8.75	-0.01	0	0	HB2	H4
SER454	8.83	-0.01	0	0	HG	H23
THR356	8.83	-0.02	0	0	H	H28
VAL235	8.93	-0.02	0	0	HG11	O3
LYS475	9.05	0.10	1	0	O	H24
GLU333	9.11	-0.04	-1	0	H	H4
GLN204	9.15	-0.01	0	0	C	H22
PHE377	9.34	-0.02	0	0	HZ	H4
LEU380	9.61	-0.01	0	0	HD12	H17
CYS487	9.70	-0.02	0	0	HB2	O17
LEU198	9.72	-0.06	0	0	HD13	H23
ARG485	9.78	0.12	1	0	H	O17
ARG218	9.80	0.03	1	0	H	H1
SER220	9.90	-0.02	0	0	HG	H4

Supplementary Table 20 – Description of the quantum mechanics calculations of the conformation 1 of HSA-FA6::DHT.

HSA Residue	Distance (Å)	Interaction Energy (kcal/mol)	Residue Charge	DHT Charge	HSA Atom	DHT Atom
LEU481	1.93	-6.91	0	0	H	O17
ALA210	2.05	-4.34	0	0	HB2	H20
LYS351	2.14	-5.51	1	0	HB3	H15
VAL482	2.22	-6.07	0	0	HG22	H16
LEU347	2.36	-5.28	0	0	HD22	H16
ALA350	2.41	-2.36	0	0	HB2	H4
ASP324	2.49	-0.92	-1	0	HB2	H6
PHE206	2.68	-2.71	0	0	HD1	H22
LEU327	2.69	-3.18	0	0	HB2	H3
SER480	2.77	-9.55	0	0	HG	H24
ARG209	2.77	-4.70	1	0	NH2	H9
ALA213	3.26	-1.65	0	0	HB1	H1
LEU331	3.29	-1.49	0	0	HD21	H4
GLU354	3.52	-1.23	-1	0	OE1	H28
GLY328	3.66	-0.17	0	0	HA2	O3
ASN483	4.00	-0.54	0	0	H	H24
ARG348	4.56	-0.38	1	0	HA	H17
GLU479	4.63	-0.50	-1	0	O	H21
PHE330	5.02	-0.31	0	0	HD2	H4
VAL325	5.37	0.19	0	0	N	O3
LYS323	5.72	-0.57	1	0	O	H5
TYR353	5.92	-0.26	0	0	HB2	H3
LEU346	5.92	-0.12	0	0	O	H2
PHE211	5.95	-0.32	0	0	H	H20
THR352	5.98	-0.28	0	0	N	H29
ARG484	6.06	-0.12	1	0	H	H24
GLY207	6.13	-0.16	0	0	O	H20
PHE326	6.14	-0.27	0	0	C	O3
VAL344	6.19	-0.21	0	0	O	H17
MET329	6.21	-0.10	0	0	H	O3
TYR319	6.22	-0.11	0	0	HE1	H5
LEU349	6.23	-0.16	0	0	C	H2
TRP214	6.29	-0.23	0	0	H	H13
LYS205	6.53	0.00	1	0	O	H22
SER202	6.60	-0.10	0	0	O	H22
GLU208	6.61	-0.15	-1	0	O	H11
VAL216	6.84	-0.09	0	0	HG12	O3
THR355	6.89	-0.09	0	0	H	H29
TYR332	6.94	-0.14	0	0	H	H4
LYS212	6.99	-0.08	1	0	H	H11
THR478	7.04	-0.07	0	0	O	H22

ALA217	7.27	-0.02	0	0	HB2	H1
VAL343	7.32	-0.06	0	0	O	H16
PHE228	7.35	-0.09	0	0	HE1	O3
LEU345	7.69	-0.07	0	0	O	H14
ALA322	7.98	-0.02	0	0	O	H6
CYS477	8.05	0.00	0	0	O	H22
CYS476	8.24	0.02	0	0	O	O17
LEU357	8.26	-0.04	0	0	HD13	H3
GLU358	8.30	-0.11	-1	0	HG2	H28
TYR334	8.39	-0.12	0	0	HB2	H4
LEU203	8.46	-0.03	0	0	HA	H20
PRO486	8.63	0.00	0	0	HD3	H24
LEU198	8.79	-0.07	0	0	HD11	H23
ARG485	8.79	0.11	1	0	H	H24
SER454	8.84	-0.01	0	0	HG	H23
ALA215	8.95	-0.04	0	0	H	H1
CYS487	9.01	-0.02	0	0	HB2	H24
ALA201	9.08	-0.02	0	0	O	H22
GLU333	9.27	-0.02	-1	0	H	H4
ALA335	9.29	-0.01	0	0	HB3	H4
PHE309	9.41	-0.08	0	0	HE2	H4
GLN204	9.47	-0.01	0	0	C	H22
LEU457	9.55	-0.01	0	0	HD11	O17
PHE377	9.78	-0.02	0	0	HZ	H3
THR356	9.80	-0.01	0	0	H	H29
LYS475	9.85	0.09	1	0	O	H24
VAL235	9.95	-0.02	0	0	HG11	O3

Supplementary Table 21 – Description of the quantum mechanics calculations of the conformation 2 of HSA-FA6::DHT.

HSA Residue	Distance (Å)	Interaction Energy (kcal/mol)	Residue Charge	DHT Charge	HSA Atom	DHT Atom
LEU481	2.04	-5.88	0	0	H	O17
ALA213	2.11	-1.81	0	0	HB1	H4
ARG209	2.15	-3.89	1	0	HB2	H6
VAL482	2.25	-3.73	0	0	HG13	H17
ALA210	2.26	-5.43	0	0	HB3	H11
LYS351	2.26	-4.18	1	0	HE2	H29
PHE206	2.36	-4.52	0	0	HD1	H20
SER480	2.42	-2.23	0	0	HG	H24
LEU347	2.59	-3.12	0	0	HD12	H14
GLU479	3.94	-0.87	-1	0	O	H21
ASN483	4.56	-0.64	0	0	H	O17
GLU354	4.86	-0.54	-1	0	OE2	H28
ASP324	5.04	-0.54	-1	0	HB2	O3
TRP214	5.15	-0.35	0	0	H	H1
PHE211	5.28	-0.67	0	0	N	H1
LEU331	5.32	-0.16	0	0	HD22	H4
LYS212	5.43	-0.47	1	0	HB3	O3
THR478	5.46	-0.17	0	0	O	H22
ARG348	5.48	-0.25	1	0	HA	H17
LYS323	5.55	-0.11	1	0	HZ2	H28
LEU327	5.58	-0.23	0	0	HD12	H3
GLY207	5.85	-0.24	0	0	O	H11
GLU208	6.03	-0.23	-1	0	C	H7
ARG484	6.24	-0.08	1	0	HE	O17
LYS205	6.24	-0.04	1	0	O	H20
GLY328	6.37	-0.07	0	0	HA2	H4
ALA350	6.80	-0.08	0	0	HB2	H2
VAL344	6.83	-0.09	0	0	HG12	H17
SER202	6.99	-0.08	0	0	HB2	H23
CYS477	7.02	-0.03	0	0	O	H22
VAL216	7.09	-0.05	0	0	HB	H4
ALA215	7.71	-0.04	0	0	H	H1
CYS476	7.77	0.01	0	0	O	O17
THR352	7.87	-0.11	0	0	N	H29
ALA217	8.29	-0.01	0	0	HB2	H4
THR355	8.42	-0.06	0	0	H	H29
VAL325	8.44	-0.02	0	0	N	O3
LEU346	8.63	-0.03	0	0	O	H2
VAL343	8.73	-0.03	0	0	O	H14
LEU349	8.78	-0.06	0	0	N	H17
VAL235	8.87	-0.02	0	0	HG11	H4

LEU203	8.96	-0.04	0	0	HA	H11
TYR353	9.08	-0.08	0	0	H	H29
LYS475	9.09	0.10	1	0	O	H22
TYR319	9.23	-0.02	0	0	HE1	H3
MET329	9.34	-0.01	0	0	H	H4
PHE228	9.40	-0.07	0	0	HE1	H4
CYS487	9.41	-0.02	0	0	HB2	O17
TYR332	9.47	-0.08	0	0	H	H4
PRO486	9.49	0.00	0	0	HD3	H24
ALA201	9.54	-0.01	0	0	O	H23
PHE326	9.55	-0.02	0	0	C	H3
PHE330	9.58	-0.03	0	0	HD2	H3
LEU198	9.58	-0.07	0	0	HD12	H23
LEU345	9.63	-0.02	0	0	O	H17
ARG485	9.89	0.13	1	0	H	O17
GLN204	9.97	-0.02	0	0	C	H20

Supplementary Table 22 – Description of the quantum mechanics calculations of the conformation 3 of HSA-FA6::DHT.

HSA Residue	Distance (Å)	Interaction Energy (kcal/mol)	Residue Charge	DHT Charge	HSA Atom	DHT Atom
VAL482	2.01	-6.81	0	0	H	O17
LEU481	2.07	-10.68	0	0	H	O17
LYS351	2.12	-6.81	1	0	HB3	H15
ALA213	2.22	-3.00	0	0	HB3	H1
PHE206	2.22	-2.92	0	0	HD1	H22
LEU327	2.25	-3.46	0	0	HD12	H3
ALA210	2.32	-3.90	0	0	HB1	H20
SER480	2.49	-7.24	0	0	HG	H24
LEU347	2.51	-6.10	0	0	HB2	H16
LEU331	2.79	-1.71	0	0	HD23	H4
ALA350	2.81	-2.18	0	0	HB2	H4
ARG209	2.88	-4.82	1	0	HB2	H9
ASP324	3.38	-0.31	-1	0	O	O3
GLU354	3.48	-1.94	-1	0	HB3	H28
GLY328	3.58	-0.32	0	0	H	O3
ASN483	3.97	-0.54	0	0	H	O17
ARG348	4.23	-0.32	1	0	HA	H17
GLU479	5.08	-0.33	-1	0	O	H21
VAL325	5.45	0.12	0	0	N	O3
TRP214	5.50	-0.35	0	0	H	H1
PHE211	5.54	-0.34	0	0	N	H11
LEU346	5.58	-0.32	0	0	O	H2
PHE330	5.63	-0.21	0	0	HD2	H4
VAL344	5.84	-0.22	0	0	HG13	H16
LYS323	5.86	-0.43	1	0	O	O3
THR352	5.89	-0.41	0	0	N	H29
THR355	5.99	-0.17	0	0	H	H29
TYR353	6.12	-0.24	0	0	HB2	H3
TYR319	6.15	-0.11	0	0	HE1	H28
ARG484	6.17	-0.11	1	0	H	O17
VAL216	6.27	-0.15	0	0	HG21	O3
MET329	6.35	-0.08	0	0	H	O3
LYS212	6.37	-0.12	1	0	HB3	H7
PHE326	6.38	-0.21	0	0	C	O3
LEU349	6.40	-0.09	0	0	C	H2
THR478	6.44	-0.10	0	0	O	H21
SER202	6.48	-0.09	0	0	HB2	H22
GLU208	6.50	-0.21	-1	0	C	H11
GLY207	6.82	-0.14	0	0	N	H20
TYR332	6.85	-0.13	0	0	H	H4
LYS205	6.89	0.04	1	0	O	H22

VAL343	6.95	-0.10	0	0	O	H16
ALA217	7.06	-0.04	0	0	HB2	H1
PHE228	7.29	-0.10	0	0	HZ	O3
CYS477	7.49	-0.02	0	0	O	H22
ALA215	7.79	-0.05	0	0	H	H1
LEU345	7.83	-0.05	0	0	O	H14
SER454	7.87	-0.02	0	0	HG	H23
LEU357	7.97	-0.04	0	0	HD11	H28
CYS476	7.98	0.01	0	0	O	O17
ALA322	8.10	-0.02	0	0	O	O3
LEU198	8.43	-0.07	0	0	HD11	H23
THR356	8.57	-0.02	0	0	H	H29
GLU358	8.57	-0.11	-1	0	HG2	H28
ALA335	8.63	-0.02	0	0	HB3	H4
TYR334	8.64	-0.11	0	0	HB3	H4
VAL235	8.80	-0.03	0	0	HG11	O3
PRO486	8.96	-0.01	0	0	HD3	O17
ARG485	9.01	0.10	1	0	H	O17
LEU203	9.17	-0.03	0	0	HA	H20
ALA201	9.25	-0.01	0	0	O	H22
GLU333	9.42	-0.04	-1	0	H	H4
LEU380	9.45	-0.01	0	0	HD13	H17
CYS487	9.49	-0.02	0	0	HB3	H24
ARG218	9.60	0.03	1	0	H	H1
PHE377	9.64	-0.02	0	0	HE1	H2
ASN458	9.67	-0.01	0	0	OD1	H22
LYS475	9.78	0.09	1	0	O	H21
GLN204	9.81	0.00	0	0	O	H20

Supplementary Table 23 – Description of the quantum mechanics calculations of the conformation 4 of HSA-FA6::DHT.

HSA Residue	Distance (Å)	Interaction Energy (kcal/mol)	Residue Charge	DHT Charge	HSA Atom	DHT Atom
LEU481	1.79	-5.89	0	0	H	O17
LEU331	1.92	-1.81	0	0	HD21	H4
LEU327	1.98	-5.00	0	0	HB2	H3
LYS351	2.09	-4.84	1	0	HZ1	H26
ALA210	2.26	-5.29	0	0	HA	H11
LEU347	2.26	-6.82	0	0	HD23	H1
ALA213	2.32	-3.47	0	0	HB1	H1
ARG209	2.35	-7.62	1	0	HB3	H9
ALA350	2.47	-2.66	0	0	HB2	H2
VAL482	2.58	-5.06	0	0	HG23	H24
GLY328	2.62	-0.79	0	0	HA2	O3
SER480	2.74	-4.74	0	0	HA	H24
ASP324	2.83	-1.40	-1	0	HA	H5
PHE206	2.88	-3.45	0	0	HD1	H22
ARG348	3.96	-0.63	1	0	HA	H17
GLU479	4.23	-0.75	-1	0	O	H21
GLU354	4.26	-1.23	-1	0	HB3	H28
ASN483	4.69	-0.44	0	0	H	O17
LYS323	4.86	-0.62	1	0	HG2	H30
TRP214	5.29	-0.38	0	0	H	H1
LEU346	5.40	-0.26	0	0	O	H2
MET329	5.44	-0.17	0	0	H	O3
PHE211	5.45	-0.41	0	0	N	H11
LYS212	5.51	-0.28	1	0	HB3	H6
PHE330	5.60	-0.24	0	0	HD2	H4
VAL325	5.72	0.06	0	0	HA	O3
VAL344	5.79	-0.25	0	0	O	H16
VAL216	5.86	-0.16	0	0	HB	O3
THR352	6.04	-0.29	0	0	H	H15
THR478	6.19	-0.10	0	0	O	H21
GLU208	6.23	-0.20	-1	0	C	H9
LEU349	6.29	-0.11	0	0	N	H14
LYS205	6.31	-0.04	1	0	O	H22
ALA217	6.32	-0.05	0	0	HB2	H4
TYR319	6.34	-0.09	0	0	HE1	H30
ARG484	6.35	-0.09	1	0	HG3	O17
PHE326	6.39	-0.04	0	0	C	O3
TYR332	6.40	-0.17	0	0	H	H4
TYR353	6.41	-0.20	0	0	HB2	H3
GLY207	6.53	-0.15	0	0	N	H20
THR355	6.78	-0.11	0	0	H	H28

VAL343	6.88	-0.11	0	0	O	H16
SER202	7.02	-0.09	0	0	HB3	H22
PHE228	7.05	-0.11	0	0	HE1	O3
CYS477	7.39	-0.01	0	0	O	H22
LEU345	7.42	-0.08	0	0	O	H14
ALA215	7.71	-0.06	0	0	H	H1
CYS476	7.79	0.02	0	0	O	O17
ALA322	8.08	-0.02	0	0	O	H5
TYR334	8.31	-0.11	0	0	HB2	H4
VAL235	8.34	-0.03	0	0	HG11	O3
ALA335	8.38	-0.01	0	0	HB2	H4
LEU357	8.60	-0.04	0	0	HD13	H3
SER454	8.79	-0.01	0	0	HG	H23
LEU198	8.80	-0.07	0	0	HD13	H23
LEU203	8.82	-0.03	0	0	HA	H20
GLU333	9.01	-0.04	-1	0	H	H4
ARG485	9.14	0.11	1	0	H	O17
PRO486	9.18	0.00	0	0	HD3	O17
ARG218	9.20	0.02	1	0	H	H1
GLU358	9.23	-0.11	-1	0	HG2	H29
ALA201	9.26	-0.01	0	0	O	H22
LYS475	9.37	0.10	1	0	O	H24
PHE309	9.38	-0.08	0	0	HE2	H3
THR356	9.41	-0.01	0	0	H	H28
SER232	9.46	0.04	0	0	OG	O3
GLN204	9.61	-0.01	0	0	C	H22
CYS487	9.75	-0.01	0	0	HB2	H24
PHE377	9.81	-0.02	0	0	HZ	H28
SER220	9.85	-0.01	0	0	HG	H4
VAL231	9.95	-0.03	0	0	HG13	O3

Supplementary Table 24 – Description of the quantum mechanics calculations of the final conformation of HSA-FA6::DHT.

HSA Residue	Distance (Å)	Interaction Energy (kcal/mol)	Residue Charge	DHT Charge	HSA Atom	DHT Atom
ALA210	1.90	-5.01	0	0	HA	H11
LEU481	2.04	-7.88	0	0	HN	O17
LEU327	2.13	-3.79	0	0	HB2	H3
ALA350	2.18	-2.82	0	0	HB2	H2
ALA213	2.21	-3.35	0	0	HB1	H7
LEU347	2.33	-7.15	0	0	HD22	H16
VAL482	2.37	-5.92	0	0	HG21	H17
LEU331	2.48	-2.74	0	0	HD21	H4
LYS351	2.52	-5.39	1	0	HB1	H15
SER480	2.60	-2.47	0	0	HG1	H24
PHE206	2.62	-4.09	0	0	HD1	H21
ASP324	2.72	-2.56	-1	0	HA	H5
GLY328	2.77	-1.02	0	0	HA2	O3
ARG209	2.78	-6.24	1	0	HB1	H9
GLU354	3.48	-1.36	-1	0	OE1	H30
GLU479	3.81	-1.08	-1	0	O	H21
ASN483	4.36	-0.68	0	0	HN	O17
ARG348	4.54	-0.56	1	0	HA	H17
VAL216	5.08	-0.24	0	0	HG12	O3
PHE211	5.08	-0.57	0	0	N	H11
VAL325	5.15	0.05	0	0	HA	O3
TRP214	5.23	-0.45	0	0	HN	H7
LYS323	5.23	-0.38	1	0	O	H5
LYS212	5.52	-0.31	1	0	HB1	H6
MET329	5.57	-0.17	0	0	HN	O3
LEU346	5.63	-0.20	0	0	O	H2
PHE330	5.86	-0.23	0	0	HB2	H4
THR478	5.90	-0.13	0	0	O	H21
TYR332	5.96	-0.14	0	0	HN	H4
GLY207	6.05	-0.18	0	0	N	H20
THR352	6.06	-0.23	0	0	HN	H15
LEU349	6.09	-0.17	0	0	C	H2
TYR353	6.11	-0.27	0	0	HD2	H3
PHE228	6.13	-0.07	0	0	HZ	O3
SER202	6.16	-0.12	0	0	HB1	H22
TYR319	6.18	-0.07	0	0	HE1	H28
VAL344	6.33	-0.19	0	0	HG12	H16
GLU208	6.37	-0.12	-1	0	C	H11
THR355	6.39	-0.10	0	0	HN	H29
LYS205	6.44	-0.22	1	0	C	H22
PHE326	6.47	-0.08	0	0	C	O3

ARG484	6.49	-0.21	1	0	HN	O17
ALA217	6.78	-0.06	0	0	HB2	H4
VAL343	7.14	-0.09	0	0	O	H14
LEU345	7.33	-0.08	0	0	O	H14
CYS477	7.57	-0.02	0	0	O	H21
CYS476	7.70	-0.01	0	0	O	O17
ALA322	7.73	-0.02	0	0	O	H5
ALA215	7.91	-0.06	0	0	HN	H7
ALA335	8.07	-0.02	0	0	HB1	H4
TYR334	8.16	-0.05	0	0	HB2	H4
VAL235	8.24	-0.03	0	0	HG12	O3
LEU203	8.40	-0.05	0	0	HA	H22
ALA201	8.63	-0.02	0	0	O	H22
GLU333	8.67	0.01	-1	0	HN	H4
LYS475	8.72	0.02	1	0	O	H24
PHE309	8.75	-0.03	0	0	HE2	H3
SER454	8.84	-0.01	0	0	OG	H23
LEU198	8.91	-0.02	0	0	HD12	H23
LEU357	8.95	-0.01	0	0	HD23	H3
THR356	9.01	-0.01	0	0	HN	H29
PRO486	9.13	-0.02	0	0	HG2	H17
GLN204	9.19	-0.03	0	0	C	H22
PHE377	9.25	-0.03	0	0	HE1	H2
SER232	9.28	0.00	0	0	OG	O3
CYS487	9.45	-0.02	0	0	HB2	H24
ARG485	9.49	-0.02	1	0	HN	O17
ARG218	9.53	-0.04	1	0	HN	H1
LEU380	9.79	-0.01	0	0	HD13	H17
GLU358	9.94	-0.08	-1	0	HB1	H28

Supplementary Table 25 – Description of the quantum mechanics calculations of the conformation 0 of HSA-FA1::TES.

HSA Residue	Distance (Å)	Interaction Energy (kcal/mol)	Residue Charge	TES Charge	HSA Atom	TES Atom
TYR138	2.12	-5.38	0	0	HA	H8
GLU141	2.31	-7.65	-1	0	HG3	H16
ILE142	2.33	-3.10	0	0	HD12	H20
PHE134	2.38	-5.84	0	0	HE1	H28
ALA126	2.41	-1.60	0	0	HB1	O3
MET123	2.44	-4.88	0	0	HG2	H26
PRO118	2.70	-4.28	0	0	HG2	H12
TYR161	2.75	-3.14	0	0	HE2	H6
ARG117	2.76	-1.35	1	0	HA	H24
LYS137	2.82	-5.84	1	0	HG3	H9
PHE165	4.00	-0.72	0	0	HZ	H27
ARG145	4.23	-0.43	1	0	HB3	H20
LEU115	4.35	-0.54	0	0	O	H22
VAL116	4.55	-0.34	0	0	O	H24
LEU135	4.60	-0.41	0	0	HA	H7
VAL122	4.69	-0.42	0	0	O	H3
THR133	4.76	-0.59	0	0	HG23	O3
PHE127	4.89	-0.39	0	0	H	H3
ARG144	5.04	-0.16	1	0	HH22	H21
LEU139	5.33	-0.44	0	0	H	H7
TYR140	5.59	-0.57	0	0	HD2	H9
LEU182	5.67	-0.23	0	0	HD11	H23
CYS124	5.84	-0.14	0	0	N	H28
LYS136	5.94	-0.21	1	0	C	H7
ASN130	6.10	-0.14	0	0	O	O3
THR125	6.16	-0.34	0	0	C	H3
GLU119	6.16	-0.21	-1	0	O	H26
ASP129	6.24	0.04	-1	0	HB2	O3
HIS146	6.33	-0.14	0	0	HD2	H19
ALA143	6.47	-0.11	0	0	N	H20
ARG114	6.57	0.03	1	0	HD2	H22
GLU131	6.88	0.08	-1	0	O	H5
VAL120	6.91	-0.12	0	0	HA	H26
ARG186	7.10	-0.11	1	0	HG3	H19
LEU178	7.14	-0.06	0	0	HD23	H27
PHE36	7.47	-0.19	0	0	HE2	O3
LEU154	7.76	-0.05	0	0	HD12	H18
GLU132	7.79	0.05	-1	0	O	H5
HIS128	7.91	-0.11	0	0	H	H3
ASP121	8.06	-0.02	-1	0	O	H3
ALA158	8.18	-0.01	0	0	HA	H8

LEU179	8.27	-0.04	0	0	HD23	H23
PHE149	8.30	-0.03	0	0	HE2	H19
LEU185	8.39	-0.03	0	0	HD21	H17
LYS162	8.42	0.05	1	0	HA	H6
CYS169	8.65	-0.02	0	0	SG	H28
ASP183	8.78	0.05	-1	0	OD1	H25
LEU24	8.93	-0.02	0	0	HD22	H7
PRO113	8.93	-0.01	0	0	O	H22
ALA28	9.17	-0.01	0	0	HB1	H20
LEU112	9.61	-0.02	0	0	HD13	H22
PHE157	9.81	-0.02	0	0	HB2	H18
LYS190	9.87	0.12	1	0	HD3	H19
ALA164	9.91	-0.01	0	0	HB2	H6

Supplementary Table 26 – Description of the quantum mechanics calculations of the conformation 1 of HSA-FA1::TES.

HSA Residue	Distance (Å)	Interaction Energy (kcal/mol)	Residue Charge	TES Charge	HSA Atom	TES Atom
LYS137	1.82	-5.20	1	0	HZ3	O3
PRO118	2.09	-4.10	0	0	HG2	H2
ARG117	2.11	-3.23	1	0	HA	H14
TYR138	2.16	-6.23	0	0	HD1	H25
TYR161	2.32	-3.79	0	0	HH	H28
GLU141	2.37	-4.41	-1	0	HG3	H7
LEU115	2.42	-4.32	0	0	HB2	H20
PHE134	2.45	-3.71	0	0	HE1	H27
MET123	2.55	-4.10	0	0	HG2	H12
ALA126	2.77	-1.80	0	0	HB1	H3
ILE142	2.92	-2.35	0	0	HD12	H19
VAL116	2.92	-2.40	0	0	O	H14
LEU182	2.95	-2.23	0	0	HD12	H23
PHE165	3.81	-1.12	0	0	HZ	H13
VAL122	4.34	-0.58	0	0	HG11	H4
THR133	4.97	-0.41	0	0	HG23	O3
ARG186	5.04	-0.24	1	0	HB3	H22
TYR140	5.23	-0.35	0	0	HD2	H7
LEU139	5.48	-0.38	0	0	N	H6
PHE127	5.58	-0.24	0	0	H	H27
LYS136	5.73	-0.09	1	0	C	H6
LEU185	5.74	-0.19	0	0	HB3	H24
LEU135	5.76	-0.20	0	0	HA	H6
HIS146	5.80	-0.05	0	0	HE2	H20
LEU179	6.07	-0.13	0	0	HD22	H15
ARG145	6.12	0.06	1	0	HG2	H20
LEU178	6.24	-0.14	0	0	HD23	H23
CYS124	6.27	-0.13	0	0	N	H27
ASP183	6.28	-0.08	-1	0	HA	H22
GLU119	6.67	-0.22	-1	0	O	H2
ARG114	6.73	0.03	1	0	O	H21
THR125	6.75	-0.01	0	0	C	H3
PHE36	6.77	-0.21	0	0	CZ	H5
ARG144	6.84	0.05	1	0	HH12	H7
VAL120	6.92	-0.15	0	0	HA	H12
ASP129	6.97	0.07	-1	0	HB2	O3
ALA143	7.23	-0.05	0	0	H	H8
LEU154	7.38	-0.05	0	0	HD12	H17
ASN130	7.79	-0.10	0	0	O	H26
LYS181	7.81	0.02	1	0	O	H24
LYS190	7.92	0.15	1	0	HE3	H20

ALA158	8.07	-0.02	0	0	HA	H25
ALA164	8.17	-0.01	0	0	HB2	H23
GLU132	8.24	0.04	-1	0	O	H5
PHE149	8.29	-0.02	0	0	HE1	H19
ASP121	8.37	-0.01	-1	0	O	H3
PRO113	8.38	-0.02	0	0	O	H18
GLU131	8.40	0.08	-1	0	O	H26
HIS128	8.43	-0.08	0	0	H	H3
PHE157	8.45	-0.04	0	0	HB2	H19
LEU24	8.67	-0.02	0	0	HD22	H6
GLU184	8.70	0.06	-1	0	N	H22
LYS162	8.83	0.06	1	0	HA	H28
ASP187	8.93	-0.09	-1	0	H	H22
GLY189	9.07	0.00	0	0	HA3	H19
CYS169	9.22	-0.02	0	0	SG	H27
VAL40	9.87	-0.03	0	0	HG23	H5
PRO180	9.91	-0.01	0	0	O	H22

Supplementary Table 27 – Description of the quantum mechanics calculations of the conformation 2 of HSA-FA1::TES.

HSA Residue	Distance (Å)	Interaction Energy (kcal/mol)	Residue Charge	TES Charge	HSA Atom	TES Atom
PRO118	2.21	-4.47	0	0	HG2	H26
TYR138	2.23	-6.16	0	0	HD1	H18
ARG117	2.29	-2.70	1	0	HG2	H24
GLU141	2.37	-7.03	-1	0	HG3	H16
MET123	2.37	-3.96	0	0	HG2	H28
PHE134	2.43	-5.93	0	0	HA	H5
ILE142	2.54	-2.53	0	0	HD11	H20
TYR161	2.55	-3.68	0	0	OH	H6
ALA126	2.65	-1.38	0	0	HB1	O3
LYS137	2.68	-5.26	1	0	HB3	H5
VAL122	3.97	-0.57	0	0	HG13	H3
VAL116	4.49	-0.41	0	0	O	H15
THR133	4.50	-0.69	0	0	HG23	O3
ARG145	4.51	-0.17	1	0	HG2	H22
PHE165	4.52	-0.60	0	0	HZ	H27
LEU115	4.59	-0.55	0	0	HB3	H15
LEU135	4.72	-0.35	0	0	HA	H7
ARG144	4.82	-0.08	1	0	HH22	H14
LEU182	4.93	-0.37	0	0	HD12	H25
ARG114	5.06	-0.07	1	0	HB2	H22
LEU139	5.55	-0.47	0	0	H	H7
PHE127	5.87	-0.24	0	0	N	O3
LYS136	5.89	-0.19	1	0	C	H7
HIS146	5.96	-0.09	0	0	HD2	H20
TYR140	5.99	-0.51	0	0	HD2	H9
ASN130	6.13	-0.19	0	0	O	O3
GLU131	6.48	0.08	-1	0	O	H5
CYS124	6.54	-0.12	0	0	N	H28
GLU119	6.59	-0.21	-1	0	O	H26
LEU178	6.72	-0.08	0	0	HD22	H27
ARG186	6.82	-0.10	1	0	HB3	H19
PHE36	6.93	-0.21	0	0	HE2	O3
ALA143	7.06	-0.06	0	0	H	H18
ASP129	7.09	0.04	-1	0	HB2	O3
GLU132	7.25	0.04	-1	0	O	H5
VAL120	7.28	-0.13	0	0	HA	H28
THR125	7.31	-0.20	0	0	C	O3
LEU154	7.61	-0.05	0	0	HD13	H18
ALA158	7.73	-0.03	0	0	HA	H8
LEU185	8.20	-0.04	0	0	HD12	H17
HIS128	8.29	-0.09	0	0	H	O3

LYS162	8.45	0.05	1	0	HA	H6
ASP121	8.45	-0.01	-1	0	C	H28
PHE149	8.58	-0.03	0	0	HZ	H20
PRO113	8.67	-0.02	0	0	O	H22
LEU179	8.68	-0.03	0	0	HD12	H25
LEU112	8.91	-0.02	0	0	HD23	H22
LEU24	9.05	-0.02	0	0	HD22	H7
ASP183	9.19	0.04	-1	0	HA	H19
PHE157	9.28	-0.03	0	0	O	H8
ALA164	9.37	-0.01	0	0	HB1	H6
CYS169	9.69	-0.02	0	0	SG	H28
LYS190	9.84	0.12	1	0	HG3	H20

Supplementary Table 28 – Description of the quantum mechanics calculations of the conformation 3 of HSA-FA1::TES.

HSA Residue	Distance (Å)	Interaction Energy (kcal/mol)	Residue Charge	TES Charge	HSA Atom	TES Atom
PHE134	2.19	-4.84	0	0	HE1	H27
LYS137	2.20	-3.66	1	0	HB3	H7
LEU115	2.22	-3.29	0	0	HD13	H21
GLU141	2.23	-5.73	-1	0	HG3	H9
TYR138	2.35	-5.65	0	0	HB3	H8
ARG117	2.38	-2.91	1	0	HG2	H25
TYR161	2.38	-3.22	0	0	HH	H6
ALA126	2.42	-0.51	0	0	HB1	O3
MET123	2.55	-5.08	0	0	HG2	H28
ILE142	2.59	-2.96	0	0	HD11	H20
PRO118	2.67	-3.61	0	0	HD3	H12
VAL116	3.03	-0.43	0	0	O	H15
VAL122	3.61	-0.79	0	0	O	H3
PHE165	3.77	-1.19	0	0	HZ	H28
LEU182	3.85	-0.74	0	0	HD12	H25
ARG145	4.59	-0.27	1	0	HB3	H20
PHE127	5.14	-0.27	0	0	H	H27
THR133	5.24	-0.32	0	0	O	H5
LEU135	5.49	-0.11	0	0	HA	H6
LEU139	5.60	-0.35	0	0	N	H8
CYS124	5.61	-0.17	0	0	N	H27
TYR140	5.61	-0.52	0	0	HD2	H9
HIS146	5.66	-0.17	0	0	HD2	H20
LYS136	5.75	-0.08	1	0	C	H7
THR125	5.84	-0.47	0	0	HB	H3
ARG186	6.30	-0.17	1	0	HB3	H19
ASP129	6.33	0.05	-1	0	HB2	O3
VAL120	6.35	-0.17	0	0	HA	H28
ARG114	6.38	0.08	1	0	C	H22
LEU179	6.46	-0.08	0	0	HD21	H25
LEU178	6.48	-0.11	0	0	HD21	H28
GLU119	6.64	-0.19	-1	0	O	H28
ARG144	6.77	0.05	1	0	HH12	H21
ALA143	6.99	-0.07	0	0	H	H18
ASN130	7.01	-0.13	0	0	O	H5
ASP121	7.21	-0.05	-1	0	O	H3
LEU185	7.24	-0.05	0	0	HD22	H17
PHE36	7.67	-0.15	0	0	HE1	H7
LEU154	7.71	-0.05	0	0	HD11	H18
HIS128	7.85	-0.11	0	0	H	H27
GLU131	8.06	0.09	-1	0	O	H5

PHE149	8.18	-0.03	0	0	HE2	H19
PRO113	8.26	-0.02	0	0	O	H22
ASP183	8.28	0.05	-1	0	OD1	H25
ALA158	8.39	-0.02	0	0	HA	H8
CYS169	8.47	-0.03	0	0	SG	H27
GLU132	8.47	0.07	-1	0	O	H5
LEU24	8.57	-0.02	0	0	HD22	H7
LYS162	8.68	0.05	1	0	HA	H6
ALA164	9.05	-0.02	0	0	HB2	H24
PHE157	9.47	-0.02	0	0	HB2	H17
LYS181	9.55	0.05	1	0	O	H23
ALA28	9.57	-0.01	0	0	HB1	H18

Supplementary Table 29 – Description of the quantum mechanics calculations of the conformation 4 of HSA-FA1::TES.

HSA Residue	Distance (Å)	Interaction Energy (kcal/mol)	Residue Charge	TES Charge	HSA Atom	TES Atom
LYS137	2.00	-3.51	1	0	HB2	H5
LEU115	2.11	-6.02	0	0	HD22	H16
GLU141	2.20	-5.27	-1	0	HB3	H9
ILE142	2.21	-3.44	0	0	HG12	H18
TYR138	2.27	-6.21	0	0	HA	H8
PRO118	2.31	-3.27	0	0	HG3	H3
TYR161	2.32	-2.95	0	0	HH	H6
LEU182	2.52	-3.36	0	0	HD22	H24
ARG186	2.55	-2.33	1	0	HB3	H22
ARG117	2.59	-3.72	1	0	HA	H12
LEU185	2.82	-3.36	0	0	HD23	H23
MET123	2.99	-2.47	0	0	SD	H27
PHE134	3.35	-2.31	0	0	HE1	H26
PHE165	3.58	-0.84	0	0	HZ	H28
VAL116	3.71	-1.07	0	0	O	H12
ARG145	4.07	-0.57	1	0	HD3	H21
TYR140	5.05	-0.57	0	0	HD2	H7
VAL122	5.21	-0.14	0	0	HG11	H3
LEU139	5.33	-0.41	0	0	N	H7
GLY189	5.64	-0.08	0	0	HA3	H20
ALA126	5.67	-0.16	0	0	HB2	H3
LYS136	5.77	-0.04	1	0	C	H7
LEU135	5.85	-0.23	0	0	O	H7
LEU178	6.00	-0.15	0	0	HD22	H27
ASP183	6.07	-0.05	-1	0	N	H25
THR133	6.14	-0.11	0	0	O	H5
ARG144	6.37	-0.05	1	0	HD3	H9
LYS181	6.37	-0.05	1	0	O	H25
ALA143	6.38	-0.12	0	0	H	H18
HIS146	6.45	-0.11	0	0	HE1	H20
PHE149	6.48	-0.05	0	0	HE1	H20
LYS190	6.52	0.07	1	0	HE3	H22
ASP187	6.56	-0.16	-1	0	N	H22
LEU154	6.59	-0.09	0	0	HD11	H18
GLU119	6.66	-0.15	-1	0	O	H3
PHE127	7.02	-0.07	0	0	HB3	H26
PHE36	7.12	-0.21	0	0	CD2	H5
VAL120	7.13	-0.12	0	0	HA	H27
GLU184	7.23	0.01	-1	0	C	H25
LEU179	7.26	-0.10	0	0	HD23	H24
GLU188	7.44	0.02	-1	0	HB2	H19

PHE157	7.68	-0.06	0	0	HB2	H17
ALA158	7.74	-0.03	0	0	HA	H17
CYS124	7.76	-0.05	0	0	N	H26
ARG114	7.88	0.04	1	0	C	H14
PRO110	8.32	-0.02	0	0	HG2	H21
LEU24	8.33	-0.02	0	0	HD21	H7
ALA164	8.63	-0.01	0	0	HB3	H23
THR125	8.87	-0.04	0	0	H	H3
LEU112	9.15	-0.03	0	0	HD13	H9
PRO180	9.17	0.00	0	0	O	H25
GLU132	9.24	0.06	-1	0	O	H7
LYS162	9.27	0.07	1	0	HG2	H6
ASN130	9.37	-0.11	0	0	O	H5
ASP121	9.44	0.01	-1	0	C	H3
GLU131	9.45	0.09	-1	0	O	H5
SER193	9.53	0.00	0	0	HB2	H20
PRO113	9.56	-0.02	0	0	O	H14
SER192	9.61	0.00	0	0	HB3	H20
CYS169	9.70	-0.01	0	0	SG	H26
ALA191	9.79	0.01	0	0	H	H20
ALA28	9.91	-0.01	0	0	HB1	H9

Supplementary Table 30 – Description of the quantum mechanics calculations of the conformation 5 of HSA-FA1::TES.

HSA Residue	Distance (Å)	Interaction Energy (kcal/mol)	Residue Charge	TES Charge	HSA Atom	TES Atom
MET123	1.97	-4.87	0	0	HE3	H27
TYR161	2.11	-4.50	0	0	HE2	H6
PHE134	2.12	-6.73	0	0	HA	H5
TYR138	2.24	-5.29	0	0	HD1	H8
GLU141	2.31	-4.60	-1	0	HG3	H16
ALA126	2.38	-1.59	0	0	HB2	H3
ARG117	2.39	-7.56	1	0	HE	O17
LYS137	2.47	-7.81	1	0	HB2	H5
PRO118	2.61	-4.26	0	0	HD3	H15
PHE165	3.96	-1.03	0	0	HZ	H27
ARG144	4.29	-0.15	1	0	HH22	H21
LEU135	4.30	-0.71	0	0	N	H5
THR133	4.31	-0.44	0	0	O	O3
ILE142	4.36	-0.70	0	0	HD13	H20
PHE36	5.01	-0.41	0	0	HE2	O3
PHE127	5.02	-0.45	0	0	H	H3
VAL122	5.16	-0.32	0	0	O	H3
VAL116	5.23	-0.20	0	0	O	H15
LEU182	5.42	-0.24	0	0	HD11	H17
CYS124	5.53	-0.19	0	0	N	H28
ARG114	5.57	-0.02	1	0	HH22	H22
ARG145	5.64	0.02	1	0	HG3	H22
LEU139	5.72	-0.40	0	0	N	H9
LYS136	5.84	-0.33	1	0	N	H5
LEU115	5.84	-0.21	0	0	HB3	H22
HIS146	5.88	-0.06	0	0	HE2	H20
ASN130	6.07	-0.19	0	0	O	O3
LEU178	6.27	-0.10	0	0	HD23	H27
GLU119	6.32	-0.22	-1	0	O	H26
GLU131	6.44	0.02	-1	0	O	H5
VAL120	6.48	-0.22	0	0	HA	H26
THR125	6.48	-0.02	0	0	C	H3
TYR140	6.66	-0.30	0	0	HD2	H9
LYS162	7.07	0.01	1	0	HA	H6
ARG186	7.16	-0.08	1	0	HH22	H19
ASP129	7.26	0.01	-1	0	HB2	H3
GLU132	7.31	0.03	-1	0	O	H5
ALA158	7.37	-0.06	0	0	HA	H8
HIS128	7.59	-0.09	0	0	H	H3
ASP121	8.11	-0.03	-1	0	C	H28
LEU112	8.24	-0.02	0	0	HD13	H22

ALA143	8.36	-0.03	0	0	H	H18
LEU154	8.38	-0.05	0	0	HD13	H18
LEU179	8.45	-0.03	0	0	HD21	H23
LEU185	8.50	-0.03	0	0	HD11	H8
CYS169	8.53	-0.03	0	0	SG	H28
ALA164	8.65	-0.02	0	0	HB1	H6
PHE157	8.85	-0.03	0	0	O	H8
PHE149	9.01	-0.02	0	0	HE1	H20
PRO113	9.10	-0.01	0	0	O	H22
GLU37	9.37	0.12	-1	0	HG3	O3
LEU24	9.55	-0.02	0	0	HD22	H5
LYS159	9.58	0.09	1	0	N	H7
VAL40	9.64	-0.03	0	0	HG23	O3
THR166	9.86	-0.03	0	0	H	H6
ARG160	9.93	0.13	1	0	C	H6

Supplementary Table 31 – Description of the quantum mechanics calculations of the conformation 0 of HSA-FA6::TES.

HSA Residue	Distance (Å)	Interaction Energy (kcal/mol)	Residue Charge	TES Charge	HSA Atom	TES Atom
LEU327	1.80	-1.67	0	0	HB2	H22
ALA213	2.20	-3.51	0	0	HB1	H28
ALA210	2.22	-5.22	0	0	HA	H6
LYS351	2.25	-5.58	1	0	HD3	H14
VAL482	2.45	-3.51	0	0	HG21	H2
ALA350	2.64	-2.16	0	0	HB3	H23
LEU347	2.65	-5.05	0	0	HD22	H27
ARG209	2.67	-3.21	1	0	HB3	H7
LEU331	2.80	-2.44	0	0	HD13	H24
LEU481	2.88	-1.65	0	0	HD22	H3
GLU354	2.90	-2.52	-1	0	HB3	H22
PHE206	2.96	-1.15	0	0	HB2	O3
ASP324	3.20	-1.66	-1	0	HA	H20
GLY328	3.97	-0.67	0	0	N	H19
TRP214	4.53	-0.57	0	0	H	H28
TYR353	4.90	-0.31	0	0	HB2	H22
ARG348	4.93	-0.25	1	0	HA	H15
PHE330	5.10	-0.24	0	0	HD2	H22
THR352	5.12	-0.31	0	0	H	H15
PHE211	5.15	-0.50	0	0	N	H6
SER480	5.28	-0.16	0	0	HA	H4
LEU346	5.30	-0.19	0	0	O	H23
LYS323	5.31	-0.22	1	0	O	H20
TYR319	5.47	-0.14	0	0	HE1	H22
LEU357	5.65	-0.11	0	0	HD13	H22
LYS212	5.71	-0.10	1	0	H	H6
LEU349	5.78	-0.52	0	0	C	H15
GLU208	6.03	-0.27	-1	0	O	H6
VAL325	6.03	-0.08	0	0	N	H20
GLY207	6.07	-0.02	0	0	HA3	H5
ASN483	6.17	-0.24	0	0	HD22	H2
GLU479	6.36	0.00	-1	0	O	H4
PHE326	6.38	0.25	0	0	C	H20
VAL216	6.44	-0.11	0	0	HB	H25
VAL344	6.47	-0.13	0	0	HA	H27
MET329	6.65	-0.03	0	0	H	H19
ALA217	6.80	-0.04	0	0	HB3	H23
THR355	6.81	-0.16	0	0	H	H22
SER202	6.88	-0.05	0	0	HB2	H3
TYR332	6.89	-0.13	0	0	H	H24
LYS205	7.03	-0.09	1	0	O	O3

ALA215	7.04	-0.05	0	0	H	H28
VAL343	7.47	-0.06	0	0	O	H23
LEU345	7.77	-0.05	0	0	O	H23
PHE228	8.00	-0.10	0	0	HZ	H19
GLU358	8.11	-0.10	-1	0	HG2	H21
ALA322	8.12	-0.02	0	0	O	H20
THR478	8.32	-0.03	0	0	O	O3
THR356	8.38	-0.02	0	0	H	H22
ARG484	8.48	0.00	1	0	HG3	H4
TYR334	8.60	-0.10	0	0	HB2	H24
PHE377	8.78	-0.02	0	0	HZ	H22
ALA335	8.87	-0.01	0	0	HB1	H24
LEU203	9.11	-0.01	0	0	HA	O3
VAL235	9.18	-0.02	0	0	HG12	H6
LEU198	9.34	-0.06	0	0	HD13	H3
GLU333	9.44	-0.06	-1	0	H	H24
CYS477	9.52	0.00	0	0	O	H4
ARG218	9.70	0.04	1	0	H	H25
PHE309	9.74	-0.09	0	0	HE2	H22
PHE374	9.90	-0.06	0	0	HE2	H22
ALA201	9.96	0.00	0	0	O	O3

Supplementary Table 32 – Description of the quantum mechanics calculations of the conformation 1 of HSA-FA6::TES.

HSA Residue	Distance (Å)	Interaction Energy (kcal/mol)	Residue Charge	TES Charge	HSA Atom	TES Atom
ALA213	2.05	-2.98	0	0	HB1	H27
ARG209	2.09	-5.66	1	0	HA	H6
VAL325	2.12	-3.53	0	0	HG23	H21
ASP324	2.17	-6.67	-1	0	HB2	H16
GLY328	2.24	-2.57	0	0	HA3	H12
LEU327	2.28	-2.77	0	0	HB3	H4
PHE228	2.51	-2.40	0	0	HZ	H15
SER232	2.55	-1.69	0	0	HB3	H24
LYS212	2.86	-3.67	1	0	HB3	H6
LEU331	3.12	-1.37	0	0	HD21	H3
VAL216	3.30	-1.90	0	0	HG11	H28
LYS351	3.96	-0.44	1	0	HZ3	O3
GLU208	4.21	-1.13	-1	0	HG2	H8
GLU354	4.43	0.23	-1	0	OE2	O3
VAL235	4.45	-0.53	0	0	HG11	H26
THR236	4.49	-0.44	0	0	HG1	H25
LYS323	4.77	-0.78	1	0	O	H1
MET329	4.85	-0.37	0	0	H	H12
ALA210	4.98	-0.23	0	0	N	H6
ALA350	5.18	-0.16	0	0	HB1	H4
LEU347	5.19	-0.22	0	0	HD23	H3
TRP214	5.26	-0.36	0	0	N	H27
PHE326	5.50	-0.35	0	0	N	H14
ALA229	5.61	-0.16	0	0	HA	H24
ALA217	5.71	-0.13	0	0	H	H28
ALA322	5.92	-0.14	0	0	HB3	H21
LYS233	6.06	-0.03	1	0	N	H24
PHE330	6.08	-0.17	0	0	HB2	H4
TYR332	6.11	-0.23	0	0	H	H2
PHE211	6.17	-0.08	0	0	H	H6
ALA215	6.38	-0.07	0	0	HB3	H26
TYR319	6.99	-0.06	0	0	HE1	H4
VAL231	7.12	-0.13	0	0	C	H24
GLY207	7.26	-0.05	0	0	O	H6
TYR353	7.65	-0.13	0	0	HD2	H4
THR239	7.80	-0.03	0	0	HG22	H6
SER220	7.84	-0.02	0	0	HG	H28
VAL482	8.25	-0.03	0	0	HG22	O3
GLU230	8.26	-0.03	-1	0	N	H24
LEU346	8.36	-0.02	0	0	O	H3
LEU234	8.45	-0.05	0	0	H	H24

ARG218	8.53	0.02	1	0	H	H28
ASP237	8.59	-0.03	-1	0	H	H25
PHE206	8.70	0.00	0	0	O	H6
GLU333	8.99	-0.07	-1	0	H	H2
THR352	9.30	-0.02	0	0	N	O3
LEU219	9.33	-0.04	0	0	H	H28
THR355	9.34	-0.06	0	0	H	O3
ALA335	9.35	-0.01	0	0	HB2	H3
GLU227	9.38	-0.06	-1	0	O	H24
ARG348	9.49	-0.01	1	0	HA	O3
LEU238	9.52	-0.03	0	0	HD21	H26
LEU357	9.56	-0.04	0	0	HD23	H4
TYR334	9.65	-0.08	0	0	HB2	H3
LEU203	9.66	-0.01	0	0	HD11	H6
GLU321	9.74	-0.05	-1	0	O	H21
LYS240	9.77	0.09	1	0	HG3	H17
LEU349	9.77	-0.03	0	0	C	H4

Supplementary Table 33 – Description of the quantum mechanics calculations of the conformation 2 of HSA-FA6::TES.

HSA Residue	Distance (Å)	Interaction Energy (kcal/mol)	Residue Charge	TES Charge	HSA Atom	TES Atom
ASP324	2.18	-2.82	-1	0	HB2	H6
ALA213	2.19	-2.12	0	0	HA	H4
ARG209	2.25	-4.92	1	0	HD3	H28
VAL216	2.27	-2.54	0	0	HG21	H1
VAL325	2.28	-2.56	0	0	HA	H7
LYS212	2.35	-4.32	1	0	HD3	H13
GLY328	2.43	-1.99	0	0	H	H5
SER232	2.72	-2.41	0	0	HB3	H21
LEU331	2.84	-1.51	0	0	HD21	H4
PHE228	3.09	-4.08	0	0	HZ	H1
LEU327	3.53	-1.36	0	0	HB3	H5
VAL235	3.64	-1.04	0	0	HG13	H12
THR236	3.95	-0.74	0	0	OG1	H15
MET329	4.53	-0.52	0	0	H	H7
PHE326	5.19	-0.35	0	0	N	H7
ALA217	5.37	-0.14	0	0	H	H4
GLU208	5.45	-0.33	-1	0	O	H26
TRP214	5.50	-0.26	0	0	N	H4
ALA229	5.53	-0.26	0	0	HA	H21
LYS323	5.55	-0.21	1	0	O	H5
ALA215	5.65	-0.13	0	0	HB3	H2
LEU347	5.88	-0.12	0	0	HD21	H4
LYS233	5.94	-0.07	1	0	N	H14
TYR332	5.95	-0.28	0	0	HB3	H1
ALA210	5.96	-0.20	0	0	HA	H3
ALA350	6.04	-0.05	0	0	HB3	O3
VAL231	6.26	-0.15	0	0	HG11	H1
PHE211	6.37	-0.02	0	0	C	H2
ALA322	6.42	-0.07	0	0	O	H7
PHE330	6.55	-0.19	0	0	H	H5
THR239	6.82	-0.07	0	0	HG1	H15
GLU354	6.91	0.12	-1	0	OE2	O3
SER220	7.59	-0.04	0	0	HG	H1
ASP237	7.72	-0.03	-1	0	H	H15
LEU234	8.04	-0.06	0	0	N	H14
ARG218	8.20	0.02	1	0	H	H4
GLU230	8.27	-0.09	-1	0	N	H21
LYS240	8.49	0.06	1	0	HD2	H22
TYR353	8.50	-0.11	0	0	HD2	O3
LEU219	8.52	-0.05	0	0	HB3	H1
GLY207	8.65	-0.03	0	0	O	H26

LEU238	8.89	-0.04	0	0	HB2	H15
LYS351	9.10	-0.01	1	0	N	O3
LEU346	9.13	-0.01	0	0	O	O3
VAL343	9.13	0.00	0	0	HG12	H4
GLU333	9.18	-0.08	-1	0	H	O3
ALA335	9.21	-0.02	0	0	HB3	H4
TYR319	9.26	-0.03	0	0	HD1	H5
GLU227	9.56	-0.08	-1	0	O	H14

Supplementary Table 34 – Description of the quantum mechanics calculations of the conformation 3 of HSA-FA6::TES.

HSA Residue	Distance (Å)	Interaction Energy (kcal/mol)	Residue Charge	TES Charge	HSA Atom	TES Atom
ARG209	2.02	-6.42	1	0	HG3	H7
GLY328	2.05	-2.37	0	0	HA3	H12
SER232	2.19	-3.08	0	0	HB3	H22
LYS212	2.23	-5.53	1	0	HD3	H18
ASP324	2.42	-4.37	-1	0	HB2	H1
ALA213	2.48	-4.33	0	0	HA	H28
VAL216	2.61	-3.71	0	0	HG22	H25
PHE228	2.66	-3.10	0	0	HZ	H15
VAL235	2.76	-2.83	0	0	HG11	H17
LEU327	2.93	-2.10	0	0	HB3	H4
LEU331	3.47	-1.44	0	0	HD22	H26
VAL325	3.54	-1.31	0	0	HA	H12
THR236	3.61	-0.81	0	0	HG1	H19
LYS351	3.99	-0.47	1	0	HZ1	O3
MET329	4.59	-0.56	0	0	H	H12
ALA210	4.64	-0.35	0	0	N	H7
VAL231	4.77	-0.30	0	0	HG11	H23
GLU354	4.86	0.13	-1	0	OE2	O3
LYS323	4.87	-0.56	1	0	O	H4
ALA350	5.00	-0.16	0	0	HB1	H3
GLU208	5.00	-0.60	-1	0	O	H7
ALA229	5.34	-0.19	0	0	HA	H22
LYS233	5.41	-0.15	1	0	N	H24
TRP214	5.44	-0.28	0	0	H	H6
LEU347	5.49	-0.20	0	0	HD22	H26
TYR332	5.49	-0.32	0	0	H	H27
PHE211	5.71	-0.05	0	0	C	H6
PHE326	5.85	-0.36	0	0	N	H2
THR239	5.93	-0.13	0	0	HG1	H17
ALA217	5.97	-0.15	0	0	H	H28
ALA215	6.03	-0.12	0	0	H	H6
TYR319	6.54	-0.05	0	0	HE1	H4
PHE330	6.65	-0.17	0	0	HD2	H3
LEU234	7.08	-0.08	0	0	H	H24
ALA322	7.19	-0.07	0	0	HB3	H14
ASP237	7.32	-0.04	-1	0	H	H19
GLY207	7.32	-0.04	0	0	O	H7
LEU219	7.48	-0.05	0	0	HD13	H25
GLU230	7.70	-0.08	-1	0	N	H22
SER220	7.75	-0.02	0	0	HG	H27
GLU333	8.07	-0.11	-1	0	H	H27

PHE206	8.28	0.01	0	0	O	H7
TYR353	8.32	-0.11	0	0	HD2	H4
GLU227	8.44	-0.08	-1	0	O	H23
LEU238	8.59	-0.03	0	0	HB2	H17
LYS240	8.67	0.07	1	0	HG2	H19
ARG218	8.74	0.05	1	0	H	H28
LEU346	8.82	-0.02	0	0	O	H26
VAL482	8.84	-0.03	0	0	HG22	O3
LEU357	8.96	-0.05	0	0	HD11	H4
ALA335	9.12	0.00	0	0	HB1	H27
LEU203	9.26	-0.02	0	0	HD11	H7
TYR334	9.55	-0.07	0	0	H	H27
SER202	9.57	-0.01	0	0	O	H7
LEU481	9.60	-0.03	0	0	HD23	H5
LEU349	9.64	-0.03	0	0	C	H3
THR355	9.66	-0.06	0	0	H	O3
THR352	9.76	-0.01	0	0	N	O3
VAL343	9.86	-0.01	0	0	HG11	H28
ARG348	9.90	0.01	1	0	N	O3

Supplementary Table 35 – Description of the quantum mechanics calculations of the final conformation of HSA-FA6::TES.

HSA Residue	Distance (Å)	Interaction Energy (kcal/mol)	Residue Charge	TES Charge	HSA Atom	TES Atom
SER232	1.94	-4.63	0	0	OG	H22
GLY328	2.16	-2.87	0	0	HA2	H28
LYS212	2.34	-4.91	1	0	HB2	H8
ASP324	2.35	-6.07	-1	0	HA	H1
ARG209	2.40	-7.41	1	0	HB2	H7
LEU331	2.54	-2.00	0	0	HD23	H27
VAL216	2.61	-3.58	0	0	HG22	H24
ALA213	2.67	-3.54	0	0	HN	H6
PHE228	2.68	-4.11	0	0	HZ	H15
VAL235	3.35	-1.40	0	0	HG13	H17
LEU327	3.49	-1.34	0	0	HB1	H4
VAL325	3.83	-1.49	0	0	HA	H12
THR236	4.05	-0.61	0	0	HG1	H19
LYS351	4.18	-2.27	1	0	HZ3	O3
LYS323	4.34	-0.69	1	0	O	H4
ALA229	4.64	-0.26	0	0	HA	H22
ALA210	4.69	-0.23	0	0	N	H7
VAL231	4.78	-0.28	0	0	HG12	H23
MET329	4.81	-0.59	0	0	HN	H12
LYS233	5.11	-0.31	1	0	N	H19
GLU354	5.11	0.32	-1	0	OE2	O3
ALA350	5.21	-0.29	0	0	HB3	H3
TYR332	5.37	-0.29	0	0	HN	H28
GLU208	5.39	-0.48	-1	0	O	H8
TRP214	5.50	-0.34	0	0	HN	H6
PHE211	5.69	-0.17	0	0	C	H6
LEU347	5.73	-0.19	0	0	HD22	H27
PHE326	5.86	-0.29	0	0	N	H12
ALA217	6.00	-0.16	0	0	HN	H26
ALA215	6.33	-0.12	0	0	HN	H6
THR239	6.42	-0.09	0	0	HG1	H17
PHE330	6.47	-0.17	0	0	HN	H28
LEU234	7.16	-0.08	0	0	N	H19
GLY207	7.25	-0.03	0	0	O	H7
ALA322	7.30	-0.12	0	0	HB1	H12
GLU230	7.31	-0.12	-1	0	N	H22
SER220	7.55	-0.04	0	0	HG1	H24
GLU227	7.59	-0.10	-1	0	O	H25
LEU219	7.72	-0.04	0	0	HD13	H23
ASP237	7.85	-0.09	-1	0	HN	H19
PHE206	8.07	-0.02	0	0	O	H5

VAL482	8.22	-0.04	0	0	HG22	O3
GLU333	8.26	-0.08	-1	0	HN	H28
TYR353	8.44	-0.09	0	0	HD2	H3
ARG218	8.67	0.01	1	0	HN	H26
LEU346	8.70	-0.03	0	0	HB2	H27
LEU238	8.74	-0.02	0	0	HB2	H17
TYR319	8.78	-0.03	0	0	HA	H4
LEU481	8.82	-0.03	0	0	HD22	H5
ALA335	9.06	-0.01	0	0	HB3	H27
LEU203	9.14	-0.02	0	0	HD23	H8
ARG348	9.14	-0.06	1	0	N	O3
THR355	9.41	-0.04	0	0	HN	O3
THR352	9.53	-0.02	0	0	HN	O3
TYR334	9.54	-0.02	0	0	HN	H28
SER202	9.59	0.00	0	0	HB1	H6
LYS240	9.60	0.04	1	0	HN	H17
LEU349	9.64	-0.03	0	0	C	O3
VAL343	9.81	0.00	0	0	HG13	H27
LEU357	9.90	-0.02	0	0	HD22	H4
